# Supplementary material for: Evidence for the major role of PH4αEFB in the prolyl 4-hydroxylation of Drosophila collagen IV
Source: bioRxiv. 2025 Aug 7:2025.08.05.668786. Preprint. [Version 2] doi: 10.1101/2025.08.05.668786 (PMC12340787; doi:10.1101/2025.08.05.668786)
Supplement: Supplement 6 [file media-6.pdf]

Mascot Search Results

PFHR9

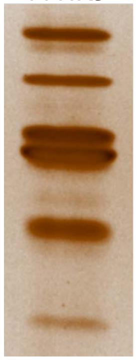

1

User :
Email :
Search title :
MS data file : ZIEN-29Nov2017_1m.mgf
Database : NCBInr 20120419 (17893860 sequences; 6141683785 residues)
Taxonomy : Mus musculus (house mouse) (138831 sequences)
Timestamp : 30 Nov 2017 at 20:13:50 GMT
Enzyme : Trypsin
Variable modifications : Carbamidomethyl (C),Oxidation (M),Oxidation (P)
Mass values : Monoisotopic
Protein Mass : Unrestricted
Peptide Mass Tolerance : ± 1.25 Da
Fragment Mass Tolerance: ± 1.001 Da
Max Missed Cleavages : 1
Instrument type : Default
Number of queries : 16088
Protein hits : gi|23271416 Leprecan 1 [Mus musculus]
gi|5852295 lysyl hydroxylase isoform 2 [Mus musculus]
gi|387397 epidermal keratin subunit I, partial [Mus musculus]
gi|6755106 procollagen-lysine,2-oxoglutarate 5-dioxygenase 1 precursor [Mus musculus]
gi|26345382 unnamed protein product [Mus musculus]
gi|4159806 type II keratin subunit protein [Mus musculus]
gi|1389682 plakoglobin, partial [Mus musculus]
gi|387398 epidermal keratin type I, partial [Mus musculus]
gi|148708988 mCG20427 [Mus musculus]
gi|12859782 unnamed protein product [Mus musculus]
gi|16303309 type II keratin 5 [Mus musculus]
gi|9790161 plakophilin-1 [Mus musculus]
gi|47523977 keratin, type II cytoskeletal 72 [Mus musculus]
gi|47059013 keratin, type II cytoskeletal 73 [Mus musculus]
gi|836898 prolyl 4-hydroxylase alpha(I)-subunit, partial [Mus musculus]
gi|220474 lamin A [Mus musculus]
gi|52785 unnamed protein product [Mus musculus]
gi|6677775 60S ribosomal protein L22 [Mus musculus]
gi|293686 epidermal keratin subunit II [Mus musculus]
gi|14917005 RecName: Full=Stress-70 protein, mitochondrial; AltName: Full=75 kDa glucose-regulated protein; Short=GRP-75; AltName: Full=Heat shock 70 kDa protein 9; AltName: Full=
gi|387207 heat shock protein [Mus musculus]
gi|50797 unnamed protein product [Mus musculus]
gi|6755110 procollagen-lysine,2-oxoglutarate 5-dioxygenase 3 precursor [Mus musculus]
gi|817996 class I (Qa) Q2k antigen [Mus musculus]
gi|19923857 thymidine phosphorylase [Mus musculus]
gi|51452 unnamed protein product [Mus musculus]
gi|398168 keratin 2 epidermis [Mus musculus]
gi|15277319 leucine zipper transcription factor-like protein 1 [Mus musculus]
gi|200765 ribonucleotide reductase subunit M1 [Mus musculus]
gi|191765 alpha-fetoprotein, partial [Mus musculus]
gi|18480346 olfactory receptor MOR175-2 [Mus musculus]
gi|226005 protein 40kD
gi|193645 glucose-regulated protein 78, partial [Mus musculus]
gi|6996913 annexin A2 [Mus musculus]
gi|116283284 Por protein [Mus musculus]
gi|12805465 Dnajc10 protein, partial [Mus musculus]
gi|148678782 mCG1027198, isoform CRA_a [Mus musculus]
gi|16716569 protease, serine, 1 precursor [Mus musculus]
gi|19924302 ankyrin repeat domain-containing protein 6 [Mus musculus]
gi|148680049 mCG147414 [Mus musculus]
gi|26337475 unnamed protein product [Mus musculus]
gi|199938 tumor-specific myb protein [Mus musculus]
gi|22094097 ganglioside-induced differentiation-associated protein 2 [Mus musculus]
gi|198683 unnamed protein product [Mus musculus]
gi|26327761 unnamed protein product [Mus musculus]
gi|51092301 type II keratin Kb14 [Mus musculus]
gi|74208129 unnamed protein product [Mus musculus]
gi|148685252 mCG142044 [Mus musculus]
gi|148671989 mCG1027581 [Mus musculus]
gi|26349347 unnamed protein product [Mus musculus]
gi|6755893 trypsin 4 precursor [Mus musculus]
gi|6754976 peroxiredoxin-1 [Mus musculus]

Select Summary Report

Format As | Select Summary (protein hits) | Help

Significance threshold p< 0.05 Max. number of hits AUTO

Standard scoring ○ MudPIT scoring ◉ Ions score or expect cut-off 43 Show sub-sets 0

Show pop-ups ◉ Suppress pop-ups ○ Require bold red ☐

Re-Search ◉ All queries ○ Unassigned ○ Below homology threshold ○ Below identity threshold

1. gi|23271416 **Mass:** 83540 **Score:** 243 **Matches:** 11(10) **Sequences:** 8(8) **emPAI:** 0.47

Leprecan 1 [Mus musculus]

Query	Observed	Mr(expt)	Mr(calc)	Delta	Miss	Score	Expect	Rank	Unique	Peptide
11282	1089.6395	1088.6322	1088.5601	0.0721	0	44	0.056	1	U	K.EIETLVEEK.T
11786	567.4809	1132.9471	1132.5360	0.4112	0	50	0.0094	1	U	R.TAIEESQAER.K
13314	644.9944	1287.9741	1287.6241	0.3500	0	60	0.00092	1	U	K.TVTAEVQPQCGR.A
14077	686.7367	1371.4588	1371.6605	-0.2017	0	61	0.0017	1	U	R.GDWPGVVLNMER.A 14085
14212	692.1389	1382.2633	1381.7677	0.4955	1	49	0.02	1	U	K.LGQEGKVPLQSAR.M
15268	743.7113	1485.4080	1485.7212	-0.3132	0	89	2.6e-006	1	U	R.AVGFSSGTENPHGVK.A
15660	773.9469	1545.8792	1545.7133	0.1659	0	61	0.0018	1	U	R.QNLDYYQTMSGVK.E
15748	781.8881	1561.7616	1561.7083	0.0533	0	(55)	0.0066	1	U	R.QNLDYYQTMSGVK.E
14531	705.4221	2113.2444	2113.9826	-0.7382	0	68	0.00038	1	U	K.AVAAAHTFFVGNPEHMEMR.Q
14649	710.9669	2129.8789	2129.9775	-0.0986	0	(45)	0.068	1	U	K.AVAAAHTFFVGNPEHMEMR.Q

Proteins matching the same set of peptides:

gi|26326437 **Mass:** 83476 **Score:** 243 **Matches:** 11(10) **Sequences:** 8(8)
unnamed protein product [Mus musculus]
gi|74205527 **Mass:** 84439 **Score:** 243 **Matches:** 11(10) **Sequences:** 8(8)
unnamed protein product [Mus musculus]
gi|109150433 **Mass:** 83598 **Score:** 243 **Matches:** 11(10) **Sequences:** 8(8)
prolyl 3-hydroxylase 1 isoform 2 precursor [Mus musculus]
gi|109150437 **Mass:** 84426 **Score:** 243 **Matches:** 11(10) **Sequences:** 8(8)
prolyl 3-hydroxylase 1 isoform 1 precursor [Mus musculus]
gi|148698509 **Mass:** 84378 **Score:** 243 **Matches:** 11(10) **Sequences:** 8(8)
leprecan 1, isoform CRA_a [Mus musculus]
gi|148698510 **Mass:** 83550 **Score:** 243 **Matches:** 11(10) **Sequences:** 8(8)
leprecan 1, isoform CRA_b [Mus musculus]
gi|12846125 **Mass:** 84033 **Score:** 243 **Matches:** 11(10) **Sequences:** 8(8)
unnamed protein product [Mus musculus]

---

2. gi|5852295 **Mass:** 84473 **Score:** 198 **Matches:** 9(9) **Sequences:** 7(7) **emPAI:** 0.31

lysyl hydroxylase isoform 2 [Mus musculus]

Query	Observed	Mr(expt)	Mr(calc)	Delta	Miss	Score	Expect	Rank	Unique	Peptide
10580	1017.5937	1016.5864	1016.5906	-0.0042	0	46	0.035	1	U	R.EFIAPVTLK.V
14359	697.9323	1393.8501	1393.6474	0.2027	0	77	2.2e-005	1	U	R.SEDYVDIVQGNR.V 14365
14882	723.4815	1444.9483	1443.7762	1.1721	0	52	0.012	1	U	K.IVFAADGLLWPDK.R
15863	800.6402	1599.2658	1599.8773	-0.6115	1	81	1.5e-005	1	U	K.IVFAADGLLWPDKR.L 15868
15907	809.6948	1617.3751	1616.8774	0.4977	0	55	0.0063	1	U	K.IFQALNGATDEVVLK.F
15920	813.1936	1624.3726	1623.8885	0.4841	0	46	0.039	1	U	K.QIGLENVWLHFIR.E
15034	731.9530	2192.8372	2192.1477	0.6895	1	58	0.0041	1	U	K.IFQALNGATDEVVLKFENGK.S

Proteins matching the same set of peptides:

gi|18204027 **Mass:** 84389 **Score:** 198 **Matches:** 9(9) **Sequences:** 7(7)
Procollagen lysine, 2-oxoglutarate 5-dioxygenase 2 [Mus musculus]
gi|148688975 **Mass:** 84401 **Score:** 198 **Matches:** 9(9) **Sequences:** 7(7)
procollagen lysine, 2-oxoglutarate 5-dioxygenase 2, isoform CRA_a [Mus musculus]
gi|148688976 **Mass:** 86812 **Score:** 198 **Matches:** 9(9) **Sequences:** 7(7)

procollagen lysine, 2-oxoglutarate 5-dioxygenase 2, isoform CRA_b [Mus musculus]
gi|218931165 **Mass:** 84435 **Score:** 198 **Matches:** 9(9) **Sequences:** 7(7)
procollagen-lysine,2-oxoglutarate 5-dioxygenase 2 isoform 2 precursor [Mus musculus]
gi|218931167 **Mass:** 86846 **Score:** 198 **Matches:** 9(9) **Sequences:** 7(7)
procollagen-lysine,2-oxoglutarate 5-dioxygenase 2 isoform 1 precursor [Mus musculus]

---

3. gi|387397 **Mass:** 57807 **Score:** 143 **Matches:** 3(3) **Sequences:** 2(2) **emPAI:** 0.12
epidermal keratin subunit I, partial [Mus musculus]

Query	Observed	Mr(expt)	Mr(calc)	Delta	Miss	Score	Expect	Rank	Unique	Peptide
14204	691.6060	1381.1974	1380.6408	0.5566	0	74	3e-005	1	U	R.ALEESNYELEGK.I 14193
14318	695.9275	1389.8404	1389.6736	0.1669	0	78	4.2e-005	1	U	K.QSLEASLAETEGR.Y

Proteins matching the same set of peptides:
gi|12852157 **Mass:** 58587 **Score:** 143 **Matches:** 3(3) **Sequences:** 2(2)
unnamed protein product [Mus musculus]
gi|26349141 **Mass:** 52653 **Score:** 143 **Matches:** 3(3) **Sequences:** 2(2)
unnamed protein product [Mus musculus]
gi|26349459 **Mass:** 49471 **Score:** 143 **Matches:** 3(3) **Sequences:** 2(2)
unnamed protein product [Mus musculus]
gi|112983636 **Mass:** 57007 **Score:** 143 **Matches:** 3(3) **Sequences:** 2(2)
keratin, type I cytoskeletal 10 [Mus musculus]
gi|116242600 **Mass:** 57735 **Score:** 143 **Matches:** 3(3) **Sequences:** 2(2)
RecName: Full=Keratin, type I cytoskeletal 10; AltName: Full=56 kDa cytokeratin; AltName: Full=Cytokeratin-10; Short=CK-10; AltName: Full=Keratin, type I cytoskeletal 59 kDa; AltName: Full=Keratin-1[illegible]

---

4. gi|6755106 **Mass:** 83542 **Score:** 105 **Matches:** 2(1) **Sequences:** 2(1) **emPAI:** 0.08
procollagen-lysine,2-oxoglutarate 5-dioxygenase 1 precursor [Mus musculus]

Query	Observed	Mr(expt)	Mr(calc)	Delta	Miss	Score	Expect	Rank	Unique	Peptide
14836	721.0568	1440.0991	1439.7370	0.3621	0	45	0.068	1	U	K.FLVEYIAPMTEK.L
15866	801.0905	1600.1665	1599.8297	0.3368	0	101	1.6e-007	1	U	R.FLGSGGFIGYAPSLSK.L

Proteins matching the same set of peptides:
gi|74207958 **Mass:** 83514 **Score:** 105 **Matches:** 2(1) **Sequences:** 2(1)
unnamed protein product [Mus musculus]

---

5. gi|26345382 **Mass:** 46296 **Score:** 89 **Matches:** 2(1) **Sequences:** 2(1) **emPAI:** 0.15
unnamed protein product [Mus musculus]

Query	Observed	Mr(expt)	Mr(calc)	Delta	Miss	Score	Expect	Rank	Unique	Peptide
15662	774.1068	1546.1991	1545.7246	0.4745	0	87	4.4e-006	1	U	R.MISFSSGGENPHGVK.A
15901	807.7070	2420.0993	2420.1437	-0.0444	0	45	0.066	1	U	K.LAAEGLGFSYAEPNYWISYGGR.Q

Proteins matching the same set of peptides:
gi|148665269 **Mass:** 61664 **Score:** 89 **Matches:** 2(1) **Sequences:** 2(1)
leprecan-like 1 [Mus musculus]
gi|27734076 **Mass:** 80104 **Score:** 89 **Matches:** 2(1) **Sequences:** 2(1)
prolyl 3-hydroxylase 2 precursor [Mus musculus]

---

6. gi|4159806 **Mass:** 65183 **Score:** 87 **Matches:** 5(5) **Sequences:** 3(3) **emPAI:** 0.16
type II keratin subunit protein [Mus musculus]

Query	Observed	Mr(expt)	Mr(calc)	Delta	Miss	Score	Expect	Rank	Unique	Peptide
11211	542.0804	1082.1462	1081.5920	0.5541	1	49	0.011	1		K.FASFIDKVR.F
14216	692.2543	1382.4940	1383.7034	-1.2094	1	47	0.042	1		K.SLNDKFASFIDK.V 14237 14244
15155	738.6367	1475.2588	1474.8144	0.4444	1	61	0.0015	2	U	R.FLEQQNKVLQTK.W

---

7. gi|1389682 **Mass:** 68068 **Score:** 87 **Matches:** 2(2) **Sequences:** 2(2) **emPAI:** 0.10
plakoglobin, partial [Mus musculus]

Query	Observed	Mr(expt)	Mr(calc)	Delta	Miss	Score	Expect	Rank	Unique	Peptide
14556	706.2423	1410.4699	1410.8017	-0.3317	0	71	0.00016	1	U	R.ALMGSPQLVAAVVR.T
14926	725.5305	1449.0465	1448.8140	0.2325	0	61	0.0013	1	U	K.LLNQPNQWPLVK.A

Proteins matching the same set of peptides:
gi|28395018 **Mass:** 81749 **Score:** 87 **Matches:** 2(2) **Sequences:** 2(2)
junction plakoglobin [Mus musculus]

---

8. gi|387398 **Mass:** 41454 **Score:** 87 **Matches:** 2(2) **Sequences:** 2(2) **emPAI:** 0.17
epidermal keratin type I, partial [Mus musculus]

Query	Observed	Mr(expt)	Mr(calc)	Delta	Miss	Score	Expect	Rank	Unique	Peptide
10673	514.9652	1027.9159	1028.5866	-0.6707	0	54	0.0037	1	U	R.VLDELTLAR.A
13972	681.9645	1361.9144	1360.6834	1.2310	0	69	0.00028	1	U	R.EVATNSELVQSGK.S

Proteins matching the same set of peptides:
gi|7106335 **Mass:** 48132 **Score:** 87 **Matches:** 2(2) **Sequences:** 2(2)
keratin, type I cytoskeletal 17 [Mus musculus]
gi|13097093 **Mass:** 30629 **Score:** 87 **Matches:** 2(2) **Sequences:** 2(2)
Krt14 protein [Mus musculus]
gi|21489935 **Mass:** 52834 **Score:** 87 **Matches:** 2(2) **Sequences:** 2(2)
keratin, type I cytoskeletal 14 [Mus musculus]
gi|148670626 **Mass:** 54668 **Score:** 87 **Matches:** 2(2) **Sequences:** 2(2)
mCG144006 [Mus musculus]

---

9. gi|148708988 **Mass:** 320241 **Score:** 85 **Matches:** 2(2) **Sequences:** 2(2) **emPAI:** 0.02
mCG20427 [Mus musculus]

Query	Observed	Mr(expt)	Mr(calc)	Delta	Miss	Score	Expect	Rank	Unique	Peptide
11741	565.5758	1129.1370	1128.6026	0.5344	0	59	0.001	1	U	K.IEVLEEELR.L
14281	694.6082	1387.2019	1386.7103	0.4916	0	68	0.00033	1	U	R.LNDSILQATEQR.R

Proteins matching the same set of peptides:
gi|190194418 **Mass:** 332706 **Score:** 85 **Matches:** 2(2) **Sequences:** 2(2)
desmoplakin [Mus musculus]

---

10. gi|12859782 **Mass:** 65586 **Score:** 84 **Matches:** 5(5) **Sequences:** 3(3) **emPAI:** 0.16
unnamed protein product [Mus musculus]

Query	Observed	Mr(expt)	Mr(calc)	Delta	Miss	Score	Expect	Rank	Unique	Peptide
11211	542.0804	1082.1462	1081.5920	0.5541	1	49	0.011	1		K.FASFIDKVR.F
14216	692.2543	1382.4940	1383.7034	-1.2094	1	47	0.042	1		K.SLNDKFASFIDK.V 14237 14244
15155	738.6367	1475.2588	1474.7780	0.4808	0	58	0.0034	3		R.FLEQQNQVLQTK.W

Proteins matching the same set of peptides:
gi|126116585 **Mass:** 65565 **Score:** 84 **Matches:** 5(5) **Sequences:** 3(3)
keratin, type II cytoskeletal 1 [Mus musculus]

---

11. gi|16303309 **Mass:** 61743 **Score:** 84 **Matches:** 3(3) **Sequences:** 2(2) **emPAI:** 0.17
type II keratin 5 [Mus musculus]

Query	Observed	Mr(expt)	Mr(calc)	Delta	Miss	Score	Expect	Rank	Unique	Peptide
11211	542.0804	1082.1462	1081.5920	0.5541	1	49	0.011	1		K.FASFIDKVR.F
14704	713.6212	1425.2279	1424.6680	0.5599	0	69	0.00024	1	U	R.VDALMDEINFMK.M
14999	729.5875	1457.1605	1456.6578	0.5027	0	(49)	0.024	1	U	R.VDALMDEINFMK.M

Proteins matching the same set of peptides:
gi|20911031 **Mass:** 61729 **Score:** 84 **Matches:** 3(3) **Sequences:** 2(2)
keratin, type II cytoskeletal 5 [Mus musculus]

---

12. gi|9790161 **Mass:** 80844 **Score:** 80 **Matches:** 1(1) **Sequences:** 1(1) **emPAI:** 0.04
plakophilin-1 [Mus musculus]

Query	Observed	Mr(expt)	Mr(calc)	Delta	Miss	Score	Expect	Rank	Unique	Peptide

15543	762.9679	1523.9212	1523.7804	0.1408	0	80	1.4e-005	1	U	R.SPNQNVQQAAAGALR.N

Proteins matching the same set of peptides:
gi|148707614 **Mass:** 78853 **Score:** 80 **Matches:** 1(1) **Sequences:** 1(1)
plakophilin 1 [Mus musculus]

---

13. gi|47523977 **Mass:** 56715 **Score:** 71 **Matches:** 2(2) **Sequences:** 2(2) **emPAI:** 0.12
keratin, type II cytoskeletal 72 [Mus musculus]

Query	Observed	Mr(expt)	Mr(calc)	Delta	Miss	Score	Expect	Rank	Unique	Peptide
11211	542.0804	1082.1462	1081.5920	0.5541	1	49	0.011	1		K.FASFIDKVR.F
15155	738.6367	1475.2588	1475.7620	-0.5032	0	66	0.0005	1	U	R.FLEQQNQVLETK.W

---

14. gi|47059013 **Mass:** 58875 **Score:** 65 **Matches:** 3(2) **Sequences:** 3(2) **emPAI:** 0.18
keratin, type II cytoskeletal 73 [Mus musculus]

Query	Observed	Mr(expt)	Mr(calc)	Delta	Miss	Score	Expect	Rank	Unique	Peptide
11211	542.0804	1082.1462	1081.5920	0.5541	1	49	0.011	1		K.FASFIDKVR.F
13933	679.6329	1357.2512	1356.7249	0.5263	0	43	0.1	1	U	R.NLDLDSIIAEVR.A
15155	738.6367	1475.2588	1474.7780	0.4808	0	58	0.0034	3		R.FLEQQNQVLQTK.W

---

15. gi|836898 **Mass:** 59960 **Score:** 64 **Matches:** 1(1) **Sequences:** 1(1) **emPAI:** 0.05
prolyl 4-hydroxylase alpha(I)-subunit, partial [Mus musculus]

Query	Observed	Mr(expt)	Mr(calc)	Delta	Miss	Score	Expect	Rank	Unique	Peptide
15762	783.5424	1565.0702	1564.7522	0.3180	0	64	0.00069	1	U	K.SAWLSGYEDPVVSR.I

Proteins matching the same set of peptides:
gi|26336999 **Mass:** 60848 **Score:** 64 **Matches:** 1(1) **Sequences:** 1(1)
unnamed protein product [Mus musculus]
gi|33859596 **Mass:** 60872 **Score:** 64 **Matches:** 1(1) **Sequences:** 1(1)
prolyl 4-hydroxylase subunit alpha-1 precursor [Mus musculus]
gi|74148153 **Mass:** 51722 **Score:** 64 **Matches:** 1(1) **Sequences:** 1(1)
unnamed protein product [Mus musculus]
gi|74224984 **Mass:** 60886 **Score:** 64 **Matches:** 1(1) **Sequences:** 1(1)
unnamed protein product [Mus musculus]
gi|74225936 **Mass:** 63769 **Score:** 64 **Matches:** 1(1) **Sequences:** 1(1)
unnamed protein product [Mus musculus]

---

16. gi|220474 **Mass:** 47506 **Score:** 62 **Matches:** 1(1) **Sequences:** 1(1) **emPAI:** 0.07
lamin A [Mus musculus]

Query	Observed	Mr(expt)	Mr(calc)	Delta	Miss	Score	Expect	Rank	Unique	Peptide
10676	515.0694	1028.1242	1027.5662	0.5581	0	62	0.00055	1	U	R.LADALQELR.A

Proteins matching the same set of peptides:
gi|52865 **Mass:** 65367 **Score:** 62 **Matches:** 1(1) **Sequences:** 1(1)
unnamed protein product [Mus musculus]
gi|9506843 **Mass:** 52620 **Score:** 62 **Matches:** 1(1) **Sequences:** 1(1)
prelamin-A/C isoform C2 [Mus musculus]
gi|12835914 **Mass:** 74255 **Score:** 62 **Matches:** 1(1) **Sequences:** 1(1)
unnamed protein product [Mus musculus]
gi|74148166 **Mass:** 74150 **Score:** 62 **Matches:** 1(1) **Sequences:** 1(1)
unnamed protein product [Mus musculus]
gi|74204231 **Mass:** 65451 **Score:** 62 **Matches:** 1(1) **Sequences:** 1(1)
unnamed protein product [Mus musculus]
gi|74219492 **Mass:** 65397 **Score:** 62 **Matches:** 1(1) **Sequences:** 1(1)
unnamed protein product [Mus musculus]
gi|74219697 **Mass:** 65335 **Score:** 62 **Matches:** 1(1) **Sequences:** 1(1)
unnamed protein product [Mus musculus]

gi|74220666 **Mass:** 72427 **Score:** 62 **Matches:** 1(1) **Sequences:** 1(1)
unnamed protein product [Mus musculus]
gi|74220835 **Mass:** 65398 **Score:** 62 **Matches:** 1(1) **Sequences:** 1(1)
unnamed protein product [Mus musculus]
gi|161760667 **Mass:** 65407 **Score:** 62 **Matches:** 1(1) **Sequences:** 1(1)
prelamin-A/C isoform C [Mus musculus]
gi|162287370 **Mass:** 74193 **Score:** 62 **Matches:** 1(1) **Sequences:** 1(1)
prelamin-A/C isoform A [Mus musculus]

---

17. gi|52785 **Mass:** 56409 **Score:** 60 **Matches:** 2(2) **Sequences:** 2(2) **emPAI:** 0.12
unnamed protein product [Mus musculus]

Query	Observed	Mr(expt)	Mr(calc)	Delta	Miss	Score	Expect	Rank	Unique	Peptide
11211	542.0804	1082.1462	1081.5920	0.5541	1	49	0.011	1		K.FASFIDKVR.F
15155	738.6367	1475.2588	1475.7984	-0.5396	1	55	0.0058	4	U	R.FLEQQNKVLETK.W

Proteins matching the same set of peptides:
gi|22164776 **Mass:** 57517 **Score:** 60 **Matches:** 2(2) **Sequences:** 2(2)
keratin, type II cytoskeletal 79 [Mus musculus]
gi|26324736 **Mass:** 58230 **Score:** 60 **Matches:** 2(2) **Sequences:** 2(2)
unnamed protein product [Mus musculus]
gi|29789317 **Mass:** 59704 **Score:** 60 **Matches:** 2(2) **Sequences:** 2(2)
keratin, type II cytoskeletal 75 [Mus musculus]
gi|39850082 **Mass:** 57817 **Score:** 60 **Matches:** 2(2) **Sequences:** 2(2)
Krt4 protein [Mus musculus]
gi|85701680 **Mass:** 62806 **Score:** 60 **Matches:** 2(2) **Sequences:** 2(2)
keratin, type II cytoskeletal 2 oral [Mus musculus]
gi|109735018 **Mass:** 58204 **Score:** 60 **Matches:** 2(2) **Sequences:** 2(2)
RIKEN cDNA 4732456N10 gene [Mus musculus]
gi|133778953 **Mass:** 56248 **Score:** 60 **Matches:** 2(2) **Sequences:** 2(2)
keratin, type II cytoskeletal 4 [Mus musculus]
gi|148672069 **Mass:** 59231 **Score:** 60 **Matches:** 2(2) **Sequences:** 2(2)
cDNA sequence BC031593 [Mus musculus]
gi|148672071 **Mass:** 57541 **Score:** 60 **Matches:** 2(2) **Sequences:** 2(2)
keratin 4, isoform CRA_b [Mus musculus]
gi|148672072 **Mass:** 63519 **Score:** 60 **Matches:** 2(2) **Sequences:** 2(2)
mCG144546 [Mus musculus]
gi|148672089 **Mass:** 62241 **Score:** 60 **Matches:** 2(2) **Sequences:** 2(2)
keratin 75 [Mus musculus]
gi|148672090 **Mass:** 58890 **Score:** 60 **Matches:** 2(2) **Sequences:** 2(2)
RIKEN cDNA 4732456N10 [Mus musculus]
gi|269914154 **Mass:** 58188 **Score:** 60 **Matches:** 2(2) **Sequences:** 2(2)
uncharacterized protein LOC239673 [Mus musculus]

---

18. gi|6677775 **Mass:** 14750 **Score:** 59 **Matches:** 1(1) **Sequences:** 1(1) **emPAI:** 0.23
60S ribosomal protein L22 [Mus musculus]

Query	Observed	Mr(expt)	Mr(calc)	Delta	Miss	Score	Expect	Rank	Unique	Peptide
12933	**622.0134**	**1242.0122**	**1241.6728**	**0.3394**	**0**	**59**	**0.001**	**1**	**U**	**K.AGNLGGGVVTIER.S**

---

19. gi|293686 **Mass:** 59414 **Score:** 58 **Matches:** 2(2) **Sequences:** 2(2) **emPAI:** 0.11
epidermal keratin subunit II [Mus musculus]

Query	Observed	Mr(expt)	Mr(calc)	Delta	Miss	Score	Expect	Rank	Unique	Peptide
11211	542.0804	1082.1462	1081.5920	0.5541	1	49	0.011	1		K.FASFIDKVR.F
13133	**634.0348**	**1266.0550**	**1265.6139**	**0.4411**	**0**	**50**	**0.021**	**1**	**U**	**R.TDAENEFVTLK.K**

---

20. gi|14917005 **Mass:** 73483 **Score:** 58 **Matches:** 2(2) **Sequences:** 2(2) **emPAI:** 0.09
RecName: Full=Stress-70 protein, mitochondrial; AltName: Full=75 kDa glucose-regulated protein; Short=GRP-75; AltName: Full=Heat shock 70 kDa protein 9; AltName: Full=Mortalin; AltName: Full=Peptide

Query	Observed	Mr(expt)	Mr(calc)	Delta	Miss	Score	Expect	Rank	Unique	Peptide
12939	**622.4032**	**1242.7918**	**1241.6728**	**1.1191**	**0**	**49**	**0.03**	**1**	**U**	**K.DAGQISGLNVLR.V**

14049	685.8440	2054.5101	2054.9545	-0.4444	0	55	0.0067	1	U	K.STNGDTFLGGEDFDQALLR.H

Proteins matching the same set of peptides:
gi|74204605 **Mass:** 73446 **Score:** 58 **Matches:** 2(2) **Sequences:** 2(2)
unnamed protein product [Mus musculus]
gi|74205924 **Mass:** 63924 **Score:** 58 **Matches:** 2(2) **Sequences:** 2(2)
unnamed protein product [Mus musculus]
gi|74225724 **Mass:** 73431 **Score:** 58 **Matches:** 2(2) **Sequences:** 2(2)
unnamed protein product [Mus musculus]
gi|162461907 **Mass:** 73416 **Score:** 58 **Matches:** 2(2) **Sequences:** 2(2)
stress-70 protein, mitochondrial [Mus musculus]

---

21. gi|387207 **Mass:** 69698 **Score:** 57 **Matches:** 1(1) **Sequences:** 1(1) **emPAI:** 0.05
heat shock protein [Mus musculus]

Query	Observed	Mr(expt)	Mr(calc)	Delta	Miss	Score	Expect	Rank	Unique	Peptide
13034	627.9152	1253.8158	1252.6088	1.2070	0	57	0.0041	1	U	R.FEELNADLFR.G

Proteins matching the same set of peptides:
gi|31560686 **Mass:** 69599 **Score:** 57 **Matches:** 1(1) **Sequences:** 1(1)
heat shock-related 70 kDa protein 2 [Mus musculus]
gi|74142040 **Mass:** 70841 **Score:** 57 **Matches:** 1(1) **Sequences:** 1(1)
unnamed protein product [Mus musculus]
gi|74179642 **Mass:** 43517 **Score:** 57 **Matches:** 1(1) **Sequences:** 1(1)
unnamed protein product [Mus musculus]
gi|74214304 **Mass:** 56662 **Score:** 57 **Matches:** 1(1) **Sequences:** 1(1)
unnamed protein product [Mus musculus]
gi|77415383 **Mass:** 62117 **Score:** 57 **Matches:** 1(1) **Sequences:** 1(1)
Hspa8 protein, partial [Mus musculus]
gi|215260057 **Mass:** 69681 **Score:** 57 **Matches:** 1(1) **Sequences:** 1(1)
heat shock protein 70-2 [Mus musculus]
gi|309319 **Mass:** 70793 **Score:** 57 **Matches:** 1(1) **Sequences:** 1(1)
heat shock protein 70 cognate [Mus musculus]
gi|13242237 **Mass:** 70827 **Score:** 57 **Matches:** 1(1) **Sequences:** 1(1)
heat shock cognate 71 kDa protein [Rattus norvegicus]
gi|42542422 **Mass:** 70828 **Score:** 57 **Matches:** 1(1) **Sequences:** 1(1)
Heat shock protein 8 [Mus musculus]
gi|63101351 **Mass:** 68736 **Score:** 57 **Matches:** 1(1) **Sequences:** 1(1)
Hspa8 protein [Mus musculus]
gi|74142813 **Mass:** 50433 **Score:** 57 **Matches:** 1(1) **Sequences:** 1(1)
unnamed protein product [Mus musculus]
gi|74143862 **Mass:** 70813 **Score:** 57 **Matches:** 1(1) **Sequences:** 1(1)
unnamed protein product [Mus musculus]
gi|74181586 **Mass:** 70855 **Score:** 57 **Matches:** 1(1) **Sequences:** 1(1)
unnamed protein product [Mus musculus]
gi|74181633 **Mass:** 70841 **Score:** 57 **Matches:** 1(1) **Sequences:** 1(1)
unnamed protein product [Mus musculus]
gi|74184057 **Mass:** 70857 **Score:** 57 **Matches:** 1(1) **Sequences:** 1(1)
unnamed protein product [Mus musculus]
gi|74186087 **Mass:** 70739 **Score:** 57 **Matches:** 1(1) **Sequences:** 1(1)
unnamed protein product [Mus musculus]
gi|74190799 **Mass:** 70813 **Score:** 57 **Matches:** 1(1) **Sequences:** 1(1)
unnamed protein product [Mus musculus]
gi|74191381 **Mass:** 70859 **Score:** 57 **Matches:** 1(1) **Sequences:** 1(1)
unnamed protein product [Mus musculus]
gi|74198858 **Mass:** 70852 **Score:** 57 **Matches:** 1(1) **Sequences:** 1(1)
unnamed protein product [Mus musculus]
gi|74198978 **Mass:** 70903 **Score:** 57 **Matches:** 1(1) **Sequences:** 1(1)
unnamed protein product [Mus musculus]

gi|74208631 **Mass:** 70827 **Score:** 57 **Matches:** 1(1) **Sequences:** 1(1)
unnamed protein product [Mus musculus]
gi|74211333 **Mass:** 68074 **Score:** 57 **Matches:** 1(1) **Sequences:** 1(1)
unnamed protein product [Mus musculus]
gi|74211667 **Mass:** 60764 **Score:** 57 **Matches:** 1(1) **Sequences:** 1(1)
unnamed protein product [Mus musculus]
gi|74214176 **Mass:** 70828 **Score:** 57 **Matches:** 1(1) **Sequences:** 1(1)
unnamed protein product [Mus musculus]
gi|74220416 **Mass:** 70755 **Score:** 57 **Matches:** 1(1) **Sequences:** 1(1)
unnamed protein product [Mus musculus]
gi|74220592 **Mass:** 70648 **Score:** 57 **Matches:** 1(1) **Sequences:** 1(1)
unnamed protein product [Mus musculus]
gi|74225511 **Mass:** 70783 **Score:** 57 **Matches:** 1(1) **Sequences:** 1(1)
unnamed protein product [Mus musculus]
gi|148693577 **Mass:** 35174 **Score:** 57 **Matches:** 1(1) **Sequences:** 1(1)
mCG5074, isoform CRA_a [Mus musculus]
gi|215261181 **Mass:** 42317 **Score:** 57 **Matches:** 1(1) **Sequences:** 1(1)
Chain A, Chaperone Complex

---

22. gi|50797 **Mass:** 50018 **Score:** 55 **Matches:** 2(1) **Sequences:** 1(1) **emPAI:** 0.07
unnamed protein product [Mus musculus]

Query	Observed	Mr(expt)	Mr(calc)	Delta	Miss	Score	Expect	Rank	Unique	Peptide
10649	513.1775	1024.3404	1024.6030	-0.2625	0	52	0.013	1	U	K.IGGIGTVPVGR.V 10648

Proteins matching the same set of peptides:
gi|556301 **Mass:** 50132 **Score:** 55 **Matches:** 2(1) **Sequences:** 1(1)
elongation factor Tu [Mus musculus]
gi|6681273 **Mass:** 50422 **Score:** 55 **Matches:** 2(1) **Sequences:** 1(1)
elongation factor 1-alpha 2 [Mus musculus]
gi|13278382 **Mass:** 50140 **Score:** 55 **Matches:** 2(1) **Sequences:** 1(1)
Eukaryotic translation elongation factor 1 alpha 1 [Mus musculus]
gi|26328693 **Mass:** 50024 **Score:** 55 **Matches:** 2(1) **Sequences:** 1(1)
unnamed protein product [Mus musculus]
gi|26345590 **Mass:** 50086 **Score:** 55 **Matches:** 2(1) **Sequences:** 1(1)
unnamed protein product [Mus musculus]
gi|28460696 **Mass:** 50082 **Score:** 55 **Matches:** 2(1) **Sequences:** 1(1)
elongation factor 1-alpha 1 [Rattus norvegicus]
gi|74195737 **Mass:** 50081 **Score:** 55 **Matches:** 2(1) **Sequences:** 1(1)
unnamed protein product [Mus musculus]
gi|74204203 **Mass:** 50072 **Score:** 55 **Matches:** 2(1) **Sequences:** 1(1)
unnamed protein product [Mus musculus]
gi|74227478 **Mass:** 50034 **Score:** 55 **Matches:** 2(1) **Sequences:** 1(1)
unnamed protein product [Mus musculus]
gi|148694454 **Mass:** 38144 **Score:** 55 **Matches:** 2(1) **Sequences:** 1(1)
mCG15232, isoform CRA_b [Mus musculus]
gi|148694456 **Mass:** 32313 **Score:** 55 **Matches:** 2(1) **Sequences:** 1(1)
mCG15232, isoform CRA_d [Mus musculus]

---

23. gi|6755110 **Mass:** 84869 **Score:** 54 **Matches:** 1(1) **Sequences:** 1(1) **emPAI:** 0.04
procollagen-lysine,2-oxoglutarate 5-dioxygenase 3 precursor [Mus musculus]

Query	Observed	Mr(expt)	Mr(calc)	Delta	Miss	Score	Expect	Rank	Unique	Peptide
14416	700.0134	1398.0123	1397.7150	0.2973	0	54	0.0092	1	U	K.LVGPEEALSAGEAR.D

Proteins matching the same set of peptides:
gi|12850403 **Mass:** 84909 **Score:** 54 **Matches:** 1(1) **Sequences:** 1(1)
unnamed protein product [Mus musculus]

---

24. gi|817996 **Mass:** 40730 **Score:** 53 **Matches:** 1(1) **Sequences:** 1(1) **emPAI:** 0.08
class I (Qa) Q2k antigen [Mus musculus]

Query	Observed	Mr(expt)	Mr(calc)	Delta	Miss	Score	Expect	Rank	Unique	Peptide
11186	1081.2339	1080.2266	1079.4883	0.7383	0	53	0.0038	1	U	R.FDSDAEKPR.Y

Proteins matching the same set of peptides:
gi|148674754 **Mass:** 40746 **Score:** 53 **Matches:** 1(1) **Sequences:** 1(1)
mCG4996 [Mus musculus]

---

25. gi|19923857 **Mass:** 49305 **Score:** 52 **Matches:** 1(1) **Sequences:** 1(1) **emPAI:** 0.07
thymidine phosphorylase [Mus musculus]

Query	Observed	Mr(expt)	Mr(calc)	Delta	Miss	Score	Expect	Rank	Unique	Peptide
15416	754.5466	1507.0786	1507.7419	-0.6634	1	52	0.0098	1	U	K.FGGAAVFPDQEKAR.E

---

26. gi|51452 **Mass:** 58833 **Score:** 52 **Matches:** 1(1) **Sequences:** 1(1) **emPAI:** 0.06
unnamed protein product [Mus musculus]

Query	Observed	Mr(expt)	Mr(calc)	Delta	Miss	Score	Expect	Rank	Unique	Peptide
15865	801.0338	1600.0531	1599.9018	0.1513	1	52	0.015	1	U	R.RGVMLAVDAVIAELK.K

Proteins matching the same set of peptides:
gi|148677951 **Mass:** 17541 **Score:** 52 **Matches:** 1(1) **Sequences:** 1(1)
mCG1033306 [Mus musculus]
gi|148700389 **Mass:** 49482 **Score:** 52 **Matches:** 1(1) **Sequences:** 1(1)
mCG116284 [Mus musculus]
gi|51455 **Mass:** 60903 **Score:** 52 **Matches:** 1(1) **Sequences:** 1(1)
heat shock protein 65 [Mus musculus]
gi|26353954 **Mass:** 60918 **Score:** 52 **Matches:** 1(1) **Sequences:** 1(1)
unnamed protein product [Mus musculus]
gi|76779273 **Mass:** 59388 **Score:** 52 **Matches:** 1(1) **Sequences:** 1(1)
Hspd1 protein, partial [Mus musculus]
gi|183396771 **Mass:** 60917 **Score:** 52 **Matches:** 1(1) **Sequences:** 1(1)
60 kDa heat shock protein, mitochondrial [Mus musculus]

---

27. gi|398168 **Mass:** 70934 **Score:** 52 **Matches:** 2(1) **Sequences:** 2(1) **emPAI:** 0.09
keratin 2 epidermis [Mus musculus]

Query	Observed	Mr(expt)	Mr(calc)	Delta	Miss	Score	Expect	Rank	Unique	Peptide
11211	542.0804	1082.1462	1081.5920	0.5541	1	49	0.011	1		K.FASFIDKVR.F
13037	628.1775	1254.3404	1253.6001	0.7404	0	44	0.077	1	U	R.GFSSGSAVVSGGSR.R

Proteins matching the same set of peptides:
gi|111308159 **Mass:** 70880 **Score:** 52 **Matches:** 2(1) **Sequences:** 2(1)
Keratin 2 [Mus musculus]
gi|124487419 **Mass:** 70880 **Score:** 52 **Matches:** 2(1) **Sequences:** 2(1)
keratin, type II cytoskeletal 2 epidermal [Mus musculus]
gi|148672075 **Mass:** 71783 **Score:** 52 **Matches:** 2(1) **Sequences:** 2(1)
mCG17605, isoform CRA_a [Mus musculus]
gi|148672076 **Mass:** 72140 **Score:** 52 **Matches:** 2(1) **Sequences:** 2(1)
mCG17605, isoform CRA_b [Mus musculus]

---

28. gi|15277319 **Mass:** 34752 **Score:** 51 **Matches:** 1(1) **Sequences:** 1(1) **emPAI:** 0.10
leucine zipper transcription factor-like protein 1 [Mus musculus]

Query	Observed	Mr(expt)	Mr(calc)	Delta	Miss	Score	Expect	Rank	Unique	Peptide
12441	595.5423	1189.0701	1189.5939	-0.5238	1	51	0.0072	1	U	K.TLNDKTENQK.S

Proteins matching the same set of peptides:
gi|26325604 **Mass:** 31688 **Score:** 51 **Matches:** 1(1) **Sequences:** 1(1)
unnamed protein product [Mus musculus]
gi|26326047 **Mass:** 34639 **Score:** 51 **Matches:** 1(1) **Sequences:** 1(1)
unnamed protein product [Mus musculus]

gi|26334199 **Mass:** 34694 **Score:** 51 **Matches:** 1(1) **Sequences:** 1(1)
unnamed protein product [Mus musculus]
gi|74181149 **Mass:** 33628 **Score:** 51 **Matches:** 1(1) **Sequences:** 1(1)
unnamed protein product [Mus musculus]

---

29. gi|200765 **Mass:** 90163 **Score:** 51 **Matches:** 1(1) **Sequences:** 1(1) **emPAI:** 0.04
ribonucleotide reductase subunit M1 [Mus musculus]

Query	Observed	Mr(expt)	Mr(calc)	Delta	Miss	Score	Expect	Rank	Unique	Peptide
15601	768.6637	1535.3128	1534.7667	0.5461	0	51	0.016	1	U	R.YPFESPEAQLLNK.Q

Proteins matching the same set of peptides:
gi|31982026 **Mass:** 90153 **Score:** 51 **Matches:** 1(1) **Sequences:** 1(1)
ribonucleoside-diphosphate reductase large subunit [Mus musculus]
gi|42490975 **Mass:** 90093 **Score:** 51 **Matches:** 1(1) **Sequences:** 1(1)
Ribonucleotide reductase M1 [Mus musculus]
gi|116283230 **Mass:** 86782 **Score:** 51 **Matches:** 1(1) **Sequences:** 1(1)
Rrm1 protein [Mus musculus]

---

30. gi|191765 **Mass:** 47195 **Score:** 51 **Matches:** 1(1) **Sequences:** 1(1) **emPAI:** 0.07
alpha-fetoprotein, partial [Mus musculus]

Query	Observed	Mr(expt)	Mr(calc)	Delta	Miss	Score	Expect	Rank	Unique	Peptide
15188	740.1958	1478.3770	1478.7881	-0.4111	0	51	0.015	1	U	K.LGEYGFQNAILVR.Y

Proteins matching the same set of peptides:
gi|74137565 **Mass:** 68688 **Score:** 51 **Matches:** 1(1) **Sequences:** 1(1)
unnamed protein product [Mus musculus]
gi|26340966 **Mass:** 68678 **Score:** 51 **Matches:** 1(1) **Sequences:** 1(1)
unnamed protein product [Mus musculus]
gi|26341396 **Mass:** 64961 **Score:** 51 **Matches:** 1(1) **Sequences:** 1(1)
unnamed protein product [Mus musculus]
gi|163310765 **Mass:** 68648 **Score:** 51 **Matches:** 1(1) **Sequences:** 1(1)
serum albumin precursor [Mus musculus]

---

31. gi|18480346 **Score:** 50 **Matches:** 1(1) **Sequences:** 1(1) **emPAI:** 0.09
olfactory receptor MOR175-2 [Mus musculus]

Query	Observed	Mr(expt)	Mr(calc)	Delta	Miss	Score	Expect	Rank	Unique	Peptide
6979	366.0798	730.1451	731.3562	-1.2111	0	50	0.013	1	U	K.INSADGR.R

Proteins matching the same set of peptides:
gi|121583707 **Score:** 50 **Matches:** 1(1) **Sequences:** 1(1)

---

32. gi|226005 **Mass:** 32732 **Score:** 50 **Matches:** 1(1) **Sequences:** 1(1) **emPAI:** 0.10
protein 40kD

Query	Observed	Mr(expt)	Mr(calc)	Delta	Miss	Score	Expect	Rank	Unique	Peptide
12564	602.6827	1203.3509	1202.6408	0.7102	0	50	0.025	1	U	K.FAAATGATPIAGR.F

Proteins matching the same set of peptides:
gi|293694 **Mass:** 32698 **Score:** 50 **Matches:** 1(1) **Sequences:** 1(1)
laminin receptor [Mus musculus]
gi|12846904 **Mass:** 32840 **Score:** 50 **Matches:** 1(1) **Sequences:** 1(1)
unnamed protein product [Mus musculus]
gi|26344606 **Mass:** 10928 **Score:** 50 **Matches:** 1(1) **Sequences:** 1(1)
unnamed protein product [Mus musculus]
gi|26350123 **Mass:** 32893 **Score:** 50 **Matches:** 1(1) **Sequences:** 1(1)
unnamed protein product [Mus musculus]
gi|62024907 **Mass:** 32821 **Score:** 50 **Matches:** 1(1) **Sequences:** 1(1)
Ribosomal protein SA [Mus musculus]

gi|82905443 **Mass:** 32936 **Score:** 50 **Matches:** 1(1) **Sequences:** 1(1)
PREDICTED: 40S ribosomal protein SA-like isoform 1 [Mus musculus]
gi|148237408 **Mass:** 32817 **Score:** 50 **Matches:** 1(1) **Sequences:** 1(1)
uncharacterized protein LOC100037080 [Xenopus laevis]
gi|148680000 **Mass:** 32817 **Score:** 50 **Matches:** 1(1) **Sequences:** 1(1)
mCG2650 [Mus musculus]
gi|148691799 **Mass:** 32640 **Score:** 50 **Matches:** 1(1) **Sequences:** 1(1)
mCG68071 [Mus musculus]
gi|171948782 **Mass:** 32829 **Score:** 50 **Matches:** 1(1) **Sequences:** 1(1)
laminin receptor [Mus musculus]

---

33. gi|193645 **Mass:** 18932 **Score:** 50 **Matches:** 1(1) **Sequences:** 1(1) **emPAI:** 0.18
glucose-regulated protein 78, partial [Mus musculus]

Query	Observed	Mr(expt)	Mr(calc)	Delta	Miss	Score	Expect	Rank	Unique	Peptide
14399	699.2704	1396.5262	1396.7813	-0.2551	0	50	0.025	1	U	K.ELEEIVQPIISK.L

Proteins matching the same set of peptides:
gi|387113 **Mass:** 16164 **Score:** 50 **Matches:** 1(1) **Sequences:** 1(1)
immunoglobulin heavy chain binding protein, partial [Mus musculus]
gi|1304157 **Mass:** 72412 **Score:** 50 **Matches:** 1(1) **Sequences:** 1(1)
78 kDa glucose-regulated protein [Mus musculus]
gi|2598562 **Mass:** 72433 **Score:** 50 **Matches:** 1(1) **Sequences:** 1(1)
BiP [Mus musculus]
gi|12835845 **Mass:** 72378 **Score:** 50 **Matches:** 1(1) **Sequences:** 1(1)
unnamed protein product [Mus musculus]
gi|74188814 **Mass:** 72305 **Score:** 50 **Matches:** 1(1) **Sequences:** 1(1)
unnamed protein product [Mus musculus]
gi|74198293 **Mass:** 72419 **Score:** 50 **Matches:** 1(1) **Sequences:** 1(1)
unnamed protein product [Mus musculus]
gi|74198974 **Mass:** 72361 **Score:** 50 **Matches:** 1(1) **Sequences:** 1(1)
unnamed protein product [Mus musculus]
gi|74207492 **Mass:** 72301 **Score:** 50 **Matches:** 1(1) **Sequences:** 1(1)
unnamed protein product [Mus musculus]
gi|74225394 **Mass:** 72337 **Score:** 50 **Matches:** 1(1) **Sequences:** 1(1)
unnamed protein product [Mus musculus]
gi|148676670 **Mass:** 56257 **Score:** 50 **Matches:** 1(1) **Sequences:** 1(1)
heat shock 70kD protein 5 (glucose-regulated protein), isoform CRA_b [Mus musculus]
gi|254540166 **Mass:** 72377 **Score:** 50 **Matches:** 1(1) **Sequences:** 1(1)
78 kDa glucose-regulated protein precursor [Mus musculus]

---

34. gi|6996913 **Mass:** 38652 **Score:** 50 **Matches:** 1(1) **Sequences:** 1(1) **emPAI:** 0.09
annexin A2 [Mus musculus]

Query	Observed	Mr(expt)	Mr(calc)	Delta	Miss	Score	Expect	Rank	Unique	Peptide
12728	611.6130	1221.2114	1221.5877	-0.3763	0	50	0.023	1	U	K.TPAQYDASELK.A

Proteins matching the same set of peptides:
gi|12849385 **Mass:** 38585 **Score:** 50 **Matches:** 1(1) **Sequences:** 1(1)
unnamed protein product [Mus musculus]
gi|74151782 **Mass:** 38636 **Score:** 50 **Matches:** 1(1) **Sequences:** 1(1)
unnamed protein product [Mus musculus]

---

35. gi|116283284 **Mass:** 72885 **Score:** 49 **Matches:** 1(1) **Sequences:** 1(1) **emPAI:** 0.05
Por protein [Mus musculus]

Query	Observed	Mr(expt)	Mr(calc)	Delta	Miss	Score	Expect	Rank	Unique	Peptide
13383	649.1569	1296.2993	1296.6363	-0.3370	0	49	0.023	1	U	R.WAAAPAGWAAPGR.R

---

36. gi|12805465 **Mass:** 54890 **Score:** 48 **Matches:** 1(1) **Sequences:** 1(1) **emPAI:** 0.06
Dnajc10 protein, partial [Mus musculus]

Query	Observed	Mr(expt)	Mr(calc)	Delta	Miss	Score	Expect	Rank	Unique	Peptide
13357	647.5936	1293.1727	1292.6976	0.4751	0	48	0.033	1	U	K.SSVLFLNSLDAK.E

Proteins matching the same set of peptides:
gi|12835910 **Mass:** 90547 **Score:** 48 **Matches:** 1(1) **Sequences:** 1(1)
unnamed protein product [Mus musculus]
gi|23270977 **Mass:** 90441 **Score:** 48 **Matches:** 1(1) **Sequences:** 1(1)
DnaJ (Hsp40) homolog, subfamily C, member 10 [Mus musculus]
gi|25140581 **Mass:** 90768 **Score:** 48 **Matches:** 1(1) **Sequences:** 1(1)
ER-resident protein ERdj5 [Mus musculus]
gi|33340135 **Mass:** 90521 **Score:** 48 **Matches:** 1(1) **Sequences:** 1(1)
endoplasmic reticulum DnaJ-PDI fusion protein 1 precursor [Mus musculus]
gi|119508443 **Mass:** 90525 **Score:** 48 **Matches:** 1(1) **Sequences:** 1(1)
dnaJ homolog subfamily C member 10 precursor [Mus musculus]
gi|148695313 **Mass:** 80660 **Score:** 48 **Matches:** 1(1) **Sequences:** 1(1)
mCG12166 [Mus musculus]
gi|329665912 **Mass:** 88825 **Score:** 48 **Matches:** 1(1) **Sequences:** 1(1)
Chain A, Crystal Structure Of Full-Length Erdj5

---

37. gi|148678782 **Mass:** 9063 **Score:** 48 **Matches:** 1(1) **Sequences:** 1(1) **emPAI:** 0.38
mCG1027198, isoform CRA_a [Mus musculus]

Query	Observed	Mr(expt)	Mr(calc)	Delta	Miss	Score	Expect	Rank	Unique	Peptide
11795	568.0061	1133.9976	1134.5451	-0.5475	1	48	0.015	1	U	K.ERMESSGAIR.I

---

38. gi|16716569 **Mass:** 26118 **Score:** 48 **Matches:** 1(1) **Sequences:** 1(1) **emPAI:** 0.13
protease, serine, 1 precursor [Mus musculus]

Query	Observed	Mr(expt)	Mr(calc)	Delta	Miss	Score	Expect	Rank	Unique	Peptide
15127	737.7551	2210.2436	2210.0967	0.1468	0	48	0.04	1	U	R.LGEHNINVLEGNEQFIDAAK.I

---

39. gi|19924302 **Mass:** 77934 **Score:** 47 **Matches:** 1(1) **Sequences:** 1(1) **emPAI:** 0.04
ankyrin repeat domain-containing protein 6 [Mus musculus]

Query	Observed	Mr(expt)	Mr(calc)	Delta	Miss	Score	Expect	Rank	Unique	Peptide
12288	588.4644	1174.9142	1174.5951	0.3192	1	47	0.02	1	U	K.VMQAPIKGCR.C

Proteins matching the same set of peptides:
gi|50510725 **Mass:** 78081 **Score:** 47 **Matches:** 1(1) **Sequences:** 1(1)
mKIAA0957 protein [Mus musculus]

---

40. gi|148680049 **Mass:** 5496 **Score:** 47 **Matches:** 1(1) **Sequences:** 1(1) **emPAI:** 0.65
mCG147414 [Mus musculus]

Query	Observed	Mr(expt)	Mr(calc)	Delta	Miss	Score	Expect	Rank	Unique	Peptide
15915	811.5035	1620.9925	1619.8770	1.1155	0	47	0.046	1	U	M.YPLAVGATSGLVTLDK.Q

---

41. gi|26337475 **Mass:** 33168 **Score:** 47 **Matches:** 1(1) **Sequences:** 1(1) **emPAI:** 0.10
unnamed protein product [Mus musculus]

Query	Observed	Mr(expt)	Mr(calc)	Delta	Miss	Score	Expect	Rank	Unique	Peptide
7984	789.6132	788.6059	789.3617	-0.7558	0	47	0.033	1	U	R.SGSSPSPR.A

Proteins matching the same set of peptides:
gi|309095 **Mass:** 135080 **Score:** 47 **Matches:** 1(1) **Sequences:** 1(1)
AE3 protein [Mus musculus]
gi|51328345 **Mass:** 136564 **Score:** 47 **Matches:** 1(1) **Sequences:** 1(1)
Slc4a3 protein [Mus musculus]
gi|165377246 **Mass:** 135288 **Score:** 47 **Matches:** 1(1) **Sequences:** 1(1)
anion exchange protein 3 [Mus musculus]

---

42. gi|199938 **Mass:** 65935 **Score:** 46 **Matches:** 1(1) **Sequences:** 1(1) **emPAI:** 0.05
tumor-specific myb protein [Mus musculus]

Query	Observed	Mr(expt)	Mr(calc)	Delta	Miss	Score	Expect	Rank	Unique	Peptide
11505	555.3855	1108.7564	1108.4715	0.2849	0	46	0.025	1	U	-.MGAPLNCPMK.S

Proteins matching the same set of peptides:
gi|199929 **Mass:** 79128 **Score:** 46 **Matches:** 1(1) **Sequences:** 1(1)
myb protein [Mus musculus]

---

43. gi|22094097 **Mass:** 56233 **Score:** 46 **Matches:** 1(1) **Sequences:** 1(1) **emPAI:** 0.06
ganglioside-induced differentiation-associated protein 2 [Mus musculus]

Query	Observed	Mr(expt)	Mr(calc)	Delta	Miss	Score	Expect	Rank	Unique	Peptide
7268	747.2545	746.2473	747.4028	-1.1555	0	46	0.04	1	U	K.GFNLAAR.F

Proteins matching the same set of peptides:
gi|26350139 **Mass:** 60978 **Score:** 46 **Matches:** 1(1) **Sequences:** 1(1)
unnamed protein product [Mus musculus]
gi|148675693 **Mass:** 60979 **Score:** 46 **Matches:** 1(1) **Sequences:** 1(1)
ganglioside-induced differentiation-associated-protein 2, isoform CRA_a [Mus musculus]

---

44. gi|198683 **Mass:** 35858 **Score:** 45 **Matches:** 1(1) **Sequences:** 1(1) **emPAI:** 0.09
unnamed protein product [Mus musculus]

Query	Observed	Mr(expt)	Mr(calc)	Delta	Miss	Score	Expect	Rank	Unique	Peptide
9577	457.5262	913.0378	912.5756	0.4622	0	45	0.024	1	U	K.LVIITAGAR.M

Proteins matching the same set of peptides:
gi|6754524 **Mass:** 36475 **Score:** 45 **Matches:** 1(1) **Sequences:** 1(1)
L-lactate dehydrogenase A chain isoform 1 [Mus musculus]
gi|7305229 **Mass:** 35889 **Score:** 45 **Matches:** 1(1) **Sequences:** 1(1)
L-lactate dehydrogenase C chain [Mus musculus]
gi|13529599 **Mass:** 34481 **Score:** 45 **Matches:** 1(1) **Sequences:** 1(1)
Ldha protein, partial [Mus musculus]
gi|44890285 **Mass:** 11164 **Score:** 45 **Matches:** 1(1) **Sequences:** 1(1)
Ldha protein [Mus musculus]
gi|74204388 **Mass:** 36461 **Score:** 45 **Matches:** 1(1) **Sequences:** 1(1)
unnamed protein product [Mus musculus]
gi|74208131 **Mass:** 36242 **Score:** 45 **Matches:** 1(1) **Sequences:** 1(1)
unnamed protein product [Mus musculus]
gi|74212250 **Mass:** 36476 **Score:** 45 **Matches:** 1(1) **Sequences:** 1(1)
unnamed protein product [Mus musculus]
gi|74217959 **Mass:** 36476 **Score:** 45 **Matches:** 1(1) **Sequences:** 1(1)
unnamed protein product [Mus musculus]
gi|157835307 **Mass:** 35801 **Score:** 45 **Matches:** 1(1) **Sequences:** 1(1)
Chain A, Characterization Of The Antigenic Sites On The Refined 3- Angstroms Resolution Structure Of Mouse Testicular Lactate Dehydrogenase C4
gi|257743039 **Mass:** 39733 **Score:** 45 **Matches:** 1(1) **Sequences:** 1(1)
L-lactate dehydrogenase A chain isoform 2 [Mus musculus]

---

45. gi|26327761 **Mass:** 90833 **Score:** 45 **Matches:** 1(1) **Sequences:** 1(1) **emPAI:** 0.04
unnamed protein product [Mus musculus]

Query	Observed	Mr(expt)	Mr(calc)	Delta	Miss	Score	Expect	Rank	Unique	Peptide
11852	571.0167	1140.0188	1139.6550	0.3638	0	45	0.026	1	U	K.LALPADSVNIK.I

Proteins matching the same set of peptides:
gi|26332879 **Mass:** 40136 **Score:** 45 **Matches:** 1(1) **Sequences:** 1(1)
unnamed protein product [Mus musculus]
gi|38257756 **Mass:** 90916 **Score:** 45 **Matches:** 1(1) **Sequences:** 1(1)
RecName: Full=Cullin-5; Short=CUL-5

gi|239051067 **Mass:** 98979 **Score:** 45 **Matches:** 1(1) **Sequences:** 1(1)
cullin-5 isoform 1 [Mus musculus]
gi|239051082 **Mass:** 95790 **Score:** 45 **Matches:** 1(1) **Sequences:** 1(1)
cullin-5 isoform 2 [Mus musculus]

---

46. gi|51092301 **Mass:** 60191 **Score:** 44 **Matches:** 1(0) **Sequences:** 1(0) **emPAI:** 0.05
type II keratin Kb14 [Mus musculus]

Query	Observed	Mr(expt)	Mr(calc)	Delta	Miss	Score	Expect	Rank	Unique	Peptide
13359	647.7845	1940.3318	1940.9996	-0.6678	1	44	0.072	1	U	R.FASFIDKVQFLEQQNK.V

Proteins matching the same set of peptides:
gi|148672085 **Mass:** 38119 **Score:** 44 **Matches:** 1(0) **Sequences:** 1(0)
mCG144996 [Mus musculus]

---

47. gi|74208129 **Mass:** 95473 **Score:** 44 **Matches:** 1(1) **Sequences:** 1(1) **emPAI:** 0.03
unnamed protein product [Mus musculus]

Query	Observed	Mr(expt)	Mr(calc)	Delta	Miss	Score	Expect	Rank	Unique	Peptide
11982	577.0237	1152.0328	1152.4758	-0.4430	0	44	0.028	1	U	R.SPSYPPPGCGK.S

Proteins matching the same set of peptides:
gi|74211952 **Mass:** 35351 **Score:** 44 **Matches:** 1(1) **Sequences:** 1(1)
unnamed protein product [Mus musculus]
gi|116248568 **Mass:** 95499 **Score:** 44 **Matches:** 1(1) **Sequences:** 1(1)
RecName: Full=Poly [ADP-ribose] polymerase 8; Short=PARP-8
gi|124486598 **Mass:** 99423 **Score:** 44 **Matches:** 1(1) **Sequences:** 1(1)
poly [ADP-ribose] polymerase 8 [Mus musculus]
gi|148686417 **Mass:** 92421 **Score:** 44 **Matches:** 1(1) **Sequences:** 1(1)
mCG119138 [Mus musculus]

---

48. gi|148685252 **Mass:** 396271 **Score:** 44 **Matches:** 1(1) **Sequences:** 1(1) **emPAI:** 0.01
mCG142044 [Mus musculus]

Query	Observed	Mr(expt)	Mr(calc)	Delta	Miss	Score	Expect	Rank	Unique	Peptide
11130	537.3717	1072.7289	1071.5924	1.1365	1	44	0.051	1	U	R.AELKLVEDR.L

Proteins matching the same set of peptides:
gi|182637561 **Mass:** 467476 **Score:** 44 **Matches:** 1(1) **Sequences:** 1(1)
RecName: Full=Dynein heavy chain 3, axonemal; AltName: Full=Axonemal beta dynein heavy chain 3; AltName: Full=Ciliary dynein heavy chain 3

---

49. gi|148671989 **Score:** 44 **Matches:** 1(1) **Sequences:** 1(1) **emPAI:** 0.24
mCG1027581 [Mus musculus]

Query	Observed	Mr(expt)	Mr(calc)	Delta	Miss	Score	Expect	Rank	Unique	Peptide
7739	387.8668	773.7190	774.4236	-0.7045	0	44	0.041	1	U	K.ISSPSLR.I

---

50. gi|26349347 **Mass:** 135092 **Score:** 44 **Matches:** 1(1) **Sequences:** 1(1) **emPAI:** 0.02
unnamed protein product [Mus musculus]

Query	Observed	Mr(expt)	Mr(calc)	Delta	Miss	Score	Expect	Rank	Unique	Peptide
13447	652.5267	1303.0388	1302.6238	0.4150	0	44	0.039	1	U	-.MVEAAPAGSGPLR.R

Proteins matching the same set of peptides:
gi|148689974 **Mass:** 136815 **Score:** 44 **Matches:** 1(1) **Sequences:** 1(1)
RIKEN cDNA 2310057J16, isoform CRA_a [Mus musculus]
gi|255069774 **Mass:** 135191 **Score:** 44 **Matches:** 1(1) **Sequences:** 1(1)
calmodulin-regulated spectrin-associated protein 3 isoform 1 [Mus musculus]
gi|255069776 **Mass:** 135091 **Score:** 44 **Matches:** 1(1) **Sequences:** 1(1)
calmodulin-regulated spectrin-associated protein 3 isoform 2 [Mus musculus]

---

51. gi|6755893 **Mass:** 26257 **Score:** 43 **Matches:** 1(0) **Sequences:** 1(0) **emPAI:** 0.13

trypsin 4 precursor [Mus musculus]

Query	Observed	Mr(expt)	Mr(calc)	Delta	Miss	Score	Expect	Rank	Unique	Peptide
15139	738.1550	2211.4433	2211.0920	0.3513	0	43	0.079	1	U	R.LGEHNINVLEGNEQFVNSAK.I

Proteins matching the same set of peptides:

gi|51010909 **Mass:** 26260 **Score:** 43 **Matches:** 1(0) **Sequences:** 1(0)
trypsin 5 precursor [Mus musculus]

gi|74203392 **Mass:** 27146 **Score:** 43 **Matches:** 1(0) **Sequences:** 1(0)
unnamed protein product [Mus musculus]

---

52. gi|6754976 **Mass:** 22162 **Score:** 43 **Matches:** 1(1) **Sequences:** 1(1) **emPAI:** 0.15
peroxiredoxin-1 [Mus musculus]

Query	Observed	Mr(expt)	Mr(calc)	Delta	Miss	Score	Expect	Rank	Unique	Peptide
11476	554.4471	1106.8797	1106.5972	0.2826	0	43	0.039	1	U	R.TIAQDYGVLK.A

Proteins matching the same set of peptides:

gi|12846314 **Mass:** 22222 **Score:** 43 **Matches:** 1(1) **Sequences:** 1(1)
unnamed protein product [Mus musculus]

gi|74198890 **Mass:** 22130 **Score:** 43 **Matches:** 1(1) **Sequences:** 1(1)
unnamed protein product [Mus musculus]

gi|148665674 **Mass:** 22248 **Score:** 43 **Matches:** 1(1) **Sequences:** 1(1)
mCG127770 [Mus musculus]

Mascot: http://www.matrixscience.com/

PFHR9

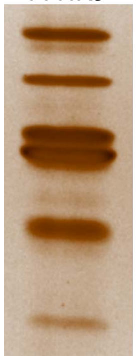

2

Mascot Search Results

```
User                    :
Email                   :
Search title            :
MS data file            : ZIEN-29Nov2017_2m.mgf
Database                : NCBInr 20120419 (17893860 sequences; 6141683785 residues)
Taxonomy                : Mus musculus (house mouse) (138831 sequences)
Timestamp               : 30 Nov 2017 at 20:24:37 GMT
Enzyme                  : Trypsin
Variable modifications  : Carbamidomethyl (C),Oxidation (P),Oxidation (M)
Mass values             : Monoisotopic
Protein Mass            : Unrestricted
Peptide Mass Tolerance  : ± 1.25 Da
Fragment Mass Tolerance : ± 1.001 Da
Max Missed Cleavages    : 1
Instrument type         : Default
Number of queries       : 16468
Protein hits            : gi|2598562   BiP [Mus musculus]
                          gi|14917005  RecName: Full=Stress-70 protein, mitochondrial; AltName: Full=75 kDa glucose-regulated protein; Short=GRP-75; AltName: Full=Heat shock 70 kDa protein 9; AltName: Full=
                          gi|74211667  unnamed protein product [Mus musculus]
                          gi|2498741   RecName: Full=Prolyl 4-hydroxylase subunit alpha-2; Short=4-PH alpha-2; AltName: Full=Procollagen-proline,2-oxoglutarate-4-dioxygenase subunit alpha-2; Flags: Precursc
                          gi|387397    epidermal keratin subunit I, partial [Mus musculus]
                          gi|46485130  TPA_exp: keratin Kb40 [Mus musculus]
                          gi|85701680  keratin, type II cytoskeletal 2 oral [Mus musculus]
                          gi|387399    epidermal keratin type I, partial [Mus musculus]
                          gi|16303309  type II keratin 5 [Mus musculus]
                          gi|26349293  unnamed protein product [Mus musculus]
                          gi|6753620   ATP-dependent RNA helicase DDX3X [Mus musculus]
                          gi|47523977  keratin, type II cytoskeletal 72 [Mus musculus]
                          gi|16716569  protease, serine, 1 precursor [Mus musculus]
                          gi|1389682   plakoglobin, partial [Mus musculus]
                          gi|19354359  Ranbp10 protein [Mus musculus]
                          gi|52789     unnamed protein product [Mus musculus]
                          gi|54777     unnamed protein product [Mus musculus]
                          gi|20070408  glycine dehydrogenase [decarboxylating], mitochondrial precursor [Mus musculus]
                          gi|3642689   immunoglobulin heavy chain-binding protein, partial [Mus musculus]
                          gi|26344548  unnamed protein product [Mus musculus]
                          gi|836898    prolyl 4-hydroxylase alpha(I)-subunit, partial [Mus musculus]
                          gi|4159806   type II keratin subunit protein [Mus musculus]
                          gi|37935731  ATP-binding cassette transporter sub-family A member 16 [Mus musculus]
                          gi|21595163  Glycosyltransferase 25 domain containing 1 [Mus musculus]
                          gi|12847276  unnamed protein product [Mus musculus]
                          gi|74191500  unnamed protein product [Mus musculus]
                          gi|81875970  RecName: Full=Alkyldihydroxyacetonephosphate synthase, peroxisomal; Short=Alkyl-DHAP synthase; AltName: Full=Alkylglycerone-phosphate synthase; Flags: Precursor
                          gi|7513694   IgG Fc binding protein - mouse (fragment)
                          gi|148664969 RIKEN cDNA 2900011O08, isoform CRA_a [Mus musculus]
                          gi|12855410  unnamed protein product [Mus musculus]
```

Select Summary Report

Format As | Select Summary (protein hits) ▾ | Help

Significance threshold p< 0.05 | Max. number of hits AUTO

Standard scoring ○ MudPIT scoring ◉ | Ions score or expect cut-off 43 | Show sub-sets 0

Show pop-ups ◉ Suppress pop-ups ○ | Require bold red ☐

Re-Search | ◉ All queries ○ Unassigned ○ Below homology threshold ○ Below identity threshold

1. gi|2598562 **Mass:** 72433 **Score:** 183 **Matches:** 10(8) **Sequences:** 6(6) **emPAI:** 0.30
BiP [Mus musculus]

Query	Observed	Mr(expt)	Mr(calc)	Delta	Miss	Score	Expect	Rank	Unique	Peptide
7641	758.4543	757.4470	757.4334	0.0136	0	51	0.011	1	U	R.NTVVPTK.K
10764	499.1691	996.3237	996.5101	-0.1863	0	49	0.012	1	U	R.ALSSQHQAR.I
13124	609.0809	1216.1473	1216.6234	-0.4761	0	52	0.011	1	U	K.DAGTIAGLNVMR.I 13134

13280	615.1929	1228.3712	1227.6207	0.7504	0	66	0.00052	1		R.VEIIANDQGNR.I
14833	699.2899	1396.5653	1396.7813	-0.2160	0	60	0.0025	1	U	K.ELEEIVQPIISK.L 14827
15459	731.0733	1460.1320	1459.7518	0.3802	0	79	2.8e-005	1	U	K.SDIDEIVLVGGSTR.I 15458 15465

Proteins matching the same set of peptides:
gi|12835845 **Mass:** 72378 **Score:** 183 **Matches:** 10(8) **Sequences:** 6(6)
unnamed protein product [Mus musculus]
gi|74188814 **Mass:** 72305 **Score:** 183 **Matches:** 10(8) **Sequences:** 6(6)
unnamed protein product [Mus musculus]
gi|74198293 **Mass:** 72419 **Score:** 183 **Matches:** 10(8) **Sequences:** 6(6)
unnamed protein product [Mus musculus]
gi|74198974 **Mass:** 72361 **Score:** 183 **Matches:** 10(8) **Sequences:** 6(6)
unnamed protein product [Mus musculus]
gi|74207492 **Mass:** 72301 **Score:** 183 **Matches:** 10(8) **Sequences:** 6(6)
unnamed protein product [Mus musculus]
gi|74225394 **Mass:** 72337 **Score:** 183 **Matches:** 10(8) **Sequences:** 6(6)
unnamed protein product [Mus musculus]
gi|254540166 **Mass:** 72377 **Score:** 183 **Matches:** 10(8) **Sequences:** 6(6)
78 kDa glucose-regulated protein precursor [Mus musculus]

---

2. gi|14917005 **Mass:** 73483 **Score:** 174 **Matches:** 10(10) **Sequences:** 7(7) **emPAI:** 0.36
RecName: Full=Stress-70 protein, mitochondrial; AltName: Full=75 kDa glucose-regulated protein; Short=GRP-75; AltName: Full=Heat shock 70 kDa protein 9; AltName: Full=Mortalin; AltName: Full=Peptide

Query	Observed	Mr(expt)	Mr(calc)	Delta	Miss	Score	Expect	Rank	Unique	Peptide
10606	978.6167	977.6095	977.4488	0.1607	0	54	0.0056	1	U	K.AMQDAEVSK.S
13304	616.1958	1230.3770	1230.6568	-0.2797	0	48	0.034	1	U	R.QAASSLQQASLK.L
13836	645.6029	1289.1913	1289.6728	-0.4816	0	56	0.0048	1	U	K.VQQTVQDLFGR.A 13846 13847
14194	667.1742	1332.3338	1332.7289	-0.3951	0	56	0.0025	1	U	R.AQFEGIVTDLIK.R
15362	726.0390	1450.0635	1449.7100	0.3535	0	60	0.00088	1	U	R.TTPSVVAFTADGER.L 15364
16185	786.0322	1570.0499	1568.8272	1.2227	0	46	0.053	1	U	K.LYSPSQIGAFVLMK.M
15239	720.0499	2157.1280	2157.0372	0.0908	1	61	0.0018	1	U	K.ERVEAVNMAEGIIHDTETK.M

Proteins matching the same set of peptides:
gi|74204605 **Mass:** 73446 **Score:** 174 **Matches:** 10(10) **Sequences:** 7(7)
unnamed protein product [Mus musculus]
gi|74225724 **Mass:** 73431 **Score:** 174 **Matches:** 10(10) **Sequences:** 7(7)
unnamed protein product [Mus musculus]
gi|162461907 **Mass:** 73416 **Score:** 174 **Matches:** 10(10) **Sequences:** 7(7)
stress-70 protein, mitochondrial [Mus musculus]

---

3. gi|74211667 **Mass:** 60764 **Score:** 169 **Matches:** 9(8) **Sequences:** 9(8) **emPAI:** 0.61
unnamed protein product [Mus musculus]

Query	Observed	Mr(expt)	Mr(calc)	Delta	Miss	Score	Expect	Rank	Unique	Peptide
7734	765.3373	764.3300	764.4068	-0.0768	0	48	0.016	1	U	K.VQVEYK.G
13280	615.1929	1228.3712	1227.6207	0.7504	0	66	0.00052	1		K.VEIIANDQGNR.T
13478	627.0481	1252.0816	1252.6088	-0.5271	0	47	0.039	1	U	R.FEELNADLFR.G
13632	634.9739	1267.9332	1267.6482	0.2850	1	54	0.0091	1	U	K.MKEIAEAYLGK.T
13950	652.5933	1303.1720	1302.5914	0.5806	0	43	0.094	1	U	K.NSLESYAFNMK.A
15105	713.9242	1425.8338	1425.6558	0.1780	1	66	0.00051	1	U	R.RFDDAVVQSDMK.H
15642	741.6528	1481.2910	1480.7998	0.4912	0	70	0.00019	1	U	K.SQIHDIVLVGGSTR.I
16318	809.0371	1616.0595	1615.7804	0.2792	0	63	0.0011	1	U	K.SFYPEEVSSMVLTK.M
15925	759.1199	2274.3378	2275.1332	-0.7954	0	49	0.023	1	U	K.SINPDEAVAYGAAVQAAILSGDK.S

Proteins matching the same set of peptides:
gi|309319 **Mass:** 70793 **Score:** 169 **Matches:** 9(8) **Sequences:** 9(8)
heat shock protein 70 cognate [Mus musculus]
gi|13242237 **Mass:** 70827 **Score:** 169 **Matches:** 9(8) **Sequences:** 9(8)
heat shock cognate 71 kDa protein [Rattus norvegicus]
gi|42542422 **Mass:** 70828 **Score:** 169 **Matches:** 9(8) **Sequences:** 9(8)

Heat shock protein 8 [Mus musculus]
gi|63101351 **Mass:** 68736 **Score:** 169 **Matches:** 9(8) **Sequences:** 9(8)
Hspa8 protein [Mus musculus]
gi|74143862 **Mass:** 70813 **Score:** 169 **Matches:** 9(8) **Sequences:** 9(8)
unnamed protein product [Mus musculus]
gi|74181586 **Mass:** 70855 **Score:** 169 **Matches:** 9(8) **Sequences:** 9(8)
unnamed protein product [Mus musculus]
gi|74184057 **Mass:** 70857 **Score:** 169 **Matches:** 9(8) **Sequences:** 9(8)
unnamed protein product [Mus musculus]
gi|74186087 **Mass:** 70739 **Score:** 169 **Matches:** 9(8) **Sequences:** 9(8)
unnamed protein product [Mus musculus]
gi|74190799 **Mass:** 70813 **Score:** 169 **Matches:** 9(8) **Sequences:** 9(8)
unnamed protein product [Mus musculus]
gi|74191381 **Mass:** 70859 **Score:** 169 **Matches:** 9(8) **Sequences:** 9(8)
unnamed protein product [Mus musculus]
gi|74198858 **Mass:** 70852 **Score:** 169 **Matches:** 9(8) **Sequences:** 9(8)
unnamed protein product [Mus musculus]
gi|74198978 **Mass:** 70903 **Score:** 169 **Matches:** 9(8) **Sequences:** 9(8)
unnamed protein product [Mus musculus]
gi|74208631 **Mass:** 70827 **Score:** 169 **Matches:** 9(8) **Sequences:** 9(8)
unnamed protein product [Mus musculus]
gi|74214176 **Mass:** 70828 **Score:** 169 **Matches:** 9(8) **Sequences:** 9(8)
unnamed protein product [Mus musculus]
gi|74220416 **Mass:** 70755 **Score:** 169 **Matches:** 9(8) **Sequences:** 9(8)
unnamed protein product [Mus musculus]
gi|74220592 **Mass:** 70648 **Score:** 169 **Matches:** 9(8) **Sequences:** 9(8)
unnamed protein product [Mus musculus]
gi|74225511 **Mass:** 70783 **Score:** 169 **Matches:** 9(8) **Sequences:** 9(8)
unnamed protein product [Mus musculus]

---

4. gi|2498741 **Mass:** 60964 **Score:** 120 **Matches:** 2(2) **Sequences:** 2(2) **emPAI:** 0.11
RecName: Full=Prolyl 4-hydroxylase subunit alpha-2; Short=4-PH alpha-2; AltName: Full=Procollagen-proline,2-oxoglutarate-4-dioxygenase subunit alpha-2; Flags: Precursor

Query	Observed	Mr(expt)	Mr(calc)	Delta	Miss	Score	Expect	Rank	Unique	Peptide
11452	534.0728	1066.1309	1065.5819	0.5491	0	74	6.3e-005	1	U	K.TGVLTVASYR.V
16319	809.4653	1616.9160	1616.7318	0.1842	0	91	1.9e-006	1	U	K.SSWLEEDDDPVVAR.V

Proteins matching the same set of peptides:
gi|74216495 **Mass:** 21055 **Score:** 120 **Matches:** 2(2) **Sequences:** 2(2)
unnamed protein product [Mus musculus]
gi|148701597 **Mass:** 57417 **Score:** 120 **Matches:** 2(2) **Sequences:** 2(2)
procollagen-proline, 2-oxoglutarate 4-dioxygenase (proline 4-hydroxylase), alpha II polypeptide, isoform CRA_b [Mus musculus]
gi|148701598 **Mass:** 21273 **Score:** 120 **Matches:** 2(2) **Sequences:** 2(2)
procollagen-proline, 2-oxoglutarate 4-dioxygenase (proline 4-hydroxylase), alpha II polypeptide, isoform CRA_c [Mus musculus]
gi|148701599 **Mass:** 61292 **Score:** 120 **Matches:** 2(2) **Sequences:** 2(2)
procollagen-proline, 2-oxoglutarate 4-dioxygenase (proline 4-hydroxylase), alpha II polypeptide, isoform CRA_d [Mus musculus]
gi|148701600 **Mass:** 66870 **Score:** 120 **Matches:** 2(2) **Sequences:** 2(2)
procollagen-proline, 2-oxoglutarate 4-dioxygenase (proline 4-hydroxylase), alpha II polypeptide, isoform CRA_e [Mus musculus]
gi|209862961 **Mass:** 60774 **Score:** 120 **Matches:** 2(2) **Sequences:** 2(2)
prolyl 4-hydroxylase subunit alpha-2 isoform 1 precursor [Mus musculus]
gi|226874876 **Mass:** 60977 **Score:** 120 **Matches:** 2(2) **Sequences:** 2(2)
prolyl 4-hydroxylase subunit alpha-2 isoform 2 precursor [Mus musculus]

---

5. gi|387397 **Mass:** 57807 **Score:** 100 **Matches:** 2(2) **Sequences:** 2(2) **emPAI:** 0.12
epidermal keratin subunit I, partial [Mus musculus]

Query	Observed	Mr(expt)	Mr(calc)	Delta	Miss	Score	Expect	Rank	Unique	Peptide
14668	691.2913	1380.5680	1380.6408	-0.0729	0	68	0.00035	1	U	R.ALEESNYELEGK.I
14765	696.1694	1390.3243	1389.6736	0.6508	0	71	0.00013	1	U	K.QSLEASLAETEGR.Y

Proteins matching the same set of peptides:
gi|12852157 **Mass:** 58587 **Score:** 100 **Matches:** 2(2) **Sequences:** 2(2)
unnamed protein product [Mus musculus]
gi|26349141 **Mass:** 52653 **Score:** 100 **Matches:** 2(2) **Sequences:** 2(2)
unnamed protein product [Mus musculus]
gi|26349459 **Mass:** 49471 **Score:** 100 **Matches:** 2(2) **Sequences:** 2(2)
unnamed protein product [Mus musculus]
gi|112983636 **Mass:** 57007 **Score:** 100 **Matches:** 2(2) **Sequences:** 2(2)
keratin, type I cytoskeletal 10 [Mus musculus]
gi|116242600 **Mass:** 57735 **Score:** 100 **Matches:** 2(2) **Sequences:** 2(2)
RecName: Full=Keratin, type I cytoskeletal 10; AltName: Full=56 kDa cytokeratin; AltName: Full=Cytokeratin-10; Short=CK-10; AltName: Full=Keratin, type I cytoskeletal 59 kDa; AltName: Full=Keratin-1[illegible]

---

6. gi|46485130 **Mass:** 85186 **Score:** 79 **Matches:** 2(2) **Sequences:** 2(2) **emPAI:** 0.08
TPA_exp: keratin Kb40 [Mus musculus]

Query	Observed	Mr(expt)	Mr(calc)	Delta	Miss	Score	Expect	Rank	Unique	Peptide
14685	**692.1215**	**1382.2284**	**1382.6830**	**-0.4546**	**0**	**57**	**0.0039**	**1**	**U**	**R.SLNNQFASFIDK.V**
15600	**739.0794**	**1476.1442**	**1475.7984**	**0.3459**	**1**	**60**	**0.0008**	**1**		**R.FLEQQNKVLETK.W**

Proteins matching the same set of peptides:
gi|111185567 **Mass:** 54730 **Score:** 79 **Matches:** 2(2) **Sequences:** 2(2)
Krt78 protein [Mus musculus]
gi|111185722 **Mass:** 56744 **Score:** 79 **Matches:** 2(2) **Sequences:** 2(2)
Krt78 protein [Mus musculus]
gi|119850791 **Mass:** 54739 **Score:** 79 **Matches:** 2(2) **Sequences:** 2(2)
Krt78 protein [Mus musculus]
gi|145580629 **Mass:** 112194 **Score:** 79 **Matches:** 2(2) **Sequences:** 2(2)
keratin Kb40 [Mus musculus]

---

7. gi|85701680 **Mass:** 62806 **Score:** 72 **Matches:** 2(2) **Sequences:** 2(2) **emPAI:** 0.11
keratin, type II cytoskeletal 2 oral [Mus musculus]

Query	Observed	Mr(expt)	Mr(calc)	Delta	Miss	Score	Expect	Rank	Unique	Peptide
11433	**1065.4515**	**1064.4442**	**1064.5138**	**-0.0696**	**0**	**54**	**0.0051**	**1**	**U**	**K.AQYEDIAQK.S**
15600	739.0794	1476.1442	1475.7984	0.3459	1	60	0.0008	1		R.FLEQQNKVLETK.W

Proteins matching the same set of peptides:
gi|148672072 **Mass:** 63519 **Score:** 72 **Matches:** 2(2) **Sequences:** 2(2)
mCG144546 [Mus musculus]

---

8. gi|387399 **Mass:** 10655 **Score:** 64 **Matches:** 1(1) **Sequences:** 1(1) **emPAI:** 0.32
epidermal keratin type I, partial [Mus musculus]

Query	Observed	Mr(expt)	Mr(calc)	Delta	Miss	Score	Expect	Rank	Unique	Peptide
16047	**770.4561**	**2308.3463**	**2308.0567**	**0.2896**	**0**	**64**	**0.00083**	**1**	**U**	**R.LLEGEDAHLSSSQFSSGSQSSR.D**

---

9. gi|16303309 **Mass:** 61743 **Score:** 62 **Matches:** 1(1) **Sequences:** 1(1) **emPAI:** 0.05
type II keratin 5 [Mus musculus]

Query	Observed	Mr(expt)	Mr(calc)	Delta	Miss	Score	Expect	Rank	Unique	Peptide
15266	**721.0629**	**1440.1112**	**1440.6629**	**-0.5517**	**0**	**62**	**0.0013**	**1**	**U**	**R.VDALMDEINFMK.M**

Proteins matching the same set of peptides:
gi|20911031 **Mass:** 61729 **Score:** 62 **Matches:** 1(1) **Sequences:** 1(1)
keratin, type II cytoskeletal 5 [Mus musculus]

---

10. gi|26349293 **Mass:** 76506 **Score:** 61 **Matches:** 1(1) **Sequences:** 1(1) **emPAI:** 0.04
unnamed protein product [Mus musculus]

Query	Observed	Mr(expt)	Mr(calc)	Delta	Miss	Score	Expect	Rank	Unique	Peptide
13424	**624.1697**	**1246.3248**	**1245.6717**	**0.6531**	**0**	**61**	**0.0014**	**1**	**U**	**R.AFINLEAAGVGGK.E**

Proteins matching the same set of peptides:
gi|37360558 **Mass:** 99638 **Score:** 61 **Matches:** 1(1) **Sequences:** 1(1)
mKIAA1815 protein [Mus musculus]
gi|74209380 **Mass:** 100058 **Score:** 61 **Matches:** 1(1) **Sequences:** 1(1)
unnamed protein product [Mus musculus]
gi|124487057 **Mass:** 100084 **Score:** 61 **Matches:** 1(1) **Sequences:** 1(1)
endoplasmic reticulum metallopeptidase 1 [Mus musculus]
gi|148709741 **Mass:** 94565 **Score:** 61 **Matches:** 1(1) **Sequences:** 1(1)
mCG124990, isoform CRA_a [Mus musculus]
gi|148709742 **Mass:** 102045 **Score:** 61 **Matches:** 1(1) **Sequences:** 1(1)
mCG124990, isoform CRA_b [Mus musculus]

---

11. gi|6753620 **Mass:** 73056 **Score:** 61 **Matches:** 1(1) **Sequences:** 1(1) **emPAI:** 0.04
ATP-dependent RNA helicase DDX3X [Mus musculus]

Query	Observed	Mr(expt)	Mr(calc)	Delta	Miss	Score	Expect	Rank	Unique	Peptide
14436	679.2409	1356.4673	1356.7765	-0.3093	0	61	0.0017	1	U	K.QYPISLVLAPTR.E

Proteins matching the same set of peptides:
gi|14861844 **Mass:** 73095 **Score:** 61 **Matches:** 1(1) **Sequences:** 1(1)
putative ATP-dependent RNA helicase Pl10 [Mus musculus]
gi|25141235 **Mass:** 73382 **Score:** 61 **Matches:** 1(1) **Sequences:** 1(1)
ATP-dependent RNA helicase DDX3Y [Mus musculus]
gi|74181660 **Mass:** 73339 **Score:** 61 **Matches:** 1(1) **Sequences:** 1(1)
unnamed protein product [Mus musculus]
gi|148706200 **Mass:** 71999 **Score:** 61 **Matches:** 1(1) **Sequences:** 1(1)
DEAD (Asp-Glu-Ala-Asp) box polypeptide 3, Y-linked, isoform CRA_a [Mus musculus]
gi|219521150 **Mass:** 73027 **Score:** 61 **Matches:** 1(1) **Sequences:** 1(1)
Ddx3x protein [Mus musculus]
gi|223462261 **Mass:** 72969 **Score:** 61 **Matches:** 1(1) **Sequences:** 1(1)
Ddx3x protein [Mus musculus]

---

12. gi|47523977 **Mass:** 56715 **Score:** 60 **Matches:** 1(1) **Sequences:** 1(1) **emPAI:** 0.06
keratin, type II cytoskeletal 72 [Mus musculus]

Query	Observed	Mr(expt)	Mr(calc)	Delta	Miss	Score	Expect	Rank	Unique	Peptide
15600	739.0794	1476.1442	1475.7620	0.3822	0	60	0.00096	2	U	R.FLEQQNQVLETK.W

---

13. gi|16716569 **Mass:** 26118 **Score:** 59 **Matches:** 3(3) **Sequences:** 1(1) **emPAI:** 0.13
protease, serine, 1 precursor [Mus musculus]

Query	Observed	Mr(expt)	Mr(calc)	Delta	Miss	Score	Expect	Rank	Unique	Peptide
15580	738.0673	2211.1800	2210.0967	1.0832	0	52	0.014	1	U	R.LGEHNINVLEGNEQFIDAAK.I 15575 15577

---

14. gi|1389682 **Mass:** 68068 **Score:** 59 **Matches:** 1(1) **Sequences:** 1(1) **emPAI:** 0.05
plakoglobin, partial [Mus musculus]

Query	Observed	Mr(expt)	Mr(calc)	Delta	Miss	Score	Expect	Rank	Unique	Peptide
15662	742.6564	1483.2982	1482.7467	0.5515	0	59	0.0027	1	U	R.NEGTATYAAAVLFR.I

Proteins matching the same set of peptides:
gi|28395018 **Mass:** 81749 **Score:** 59 **Matches:** 1(1) **Sequences:** 1(1)
junction plakoglobin [Mus musculus]

---

15. gi|19354359 **Mass:** 54698 **Score:** 59 **Matches:** 1(1) **Sequences:** 1(1) **emPAI:** 0.06
Ranbp10 protein [Mus musculus]

Query	Observed	Mr(expt)	Mr(calc)	Delta	Miss	Score	Expect	Rank	Unique	Peptide
11862	552.3564	1102.6982	1102.5618	0.1363	0	59	0.0018	1	U	R.VGEAIETTQR.F

Proteins matching the same set of peptides:
gi|50510939 **Mass:** 66515 **Score:** 59 **Matches:** 1(1) **Sequences:** 1(1)

mKIAA1464 protein [Mus musculus]
gi|353526275 **Mass:** 67146 **Score:** 59 **Matches:** 1(1) **Sequences:** 1(1)
RecName: Full=Ran-binding protein 10; Short=RanBP10
gi|40804757 **Mass:** 70044 **Score:** 59 **Matches:** 1(1) **Sequences:** 1(1)
ran-binding protein 10 [Mus musculus]

---

16. gi|52789 **Mass:** 54415 **Score:** 57 **Matches:** 1(1) **Sequences:** 1(1) **emPAI:** 0.06
unnamed protein product [Mus musculus]

Query	Observed	Mr(expt)	Mr(calc)	Delta	Miss	Score	Expect	Rank	Unique	Peptide
14685	692.1215	1382.2284	1382.7194	-0.4910	1	57	0.0039	1	U	K.SLNNKFASFIDK.V

Proteins matching the same set of peptides:
gi|309215 **Mass:** 53210 **Score:** 57 **Matches:** 1(1) **Sequences:** 1(1)
EndoA' cytokeratin (5' end put.); putative [Mus musculus]
gi|511654 **Mass:** 54220 **Score:** 57 **Matches:** 1(1) **Sequences:** 1(1)
keratin type II [Mus musculus]
gi|74177777 **Mass:** 54546 **Score:** 57 **Matches:** 1(1) **Sequences:** 1(1)
unnamed protein product [Mus musculus]
gi|74219975 **Mass:** 54459 **Score:** 57 **Matches:** 1(1) **Sequences:** 1(1)
unnamed protein product [Mus musculus]
gi|76779293 **Mass:** 54514 **Score:** 57 **Matches:** 1(1) **Sequences:** 1(1)
Keratin 8 [Mus musculus]
gi|114145561 **Mass:** 54531 **Score:** 57 **Matches:** 1(1) **Sequences:** 1(1)
keratin, type II cytoskeletal 8 [Mus musculus]

---

17. gi|54777 **Mass:** 57108 **Score:** 56 **Matches:** 1(1) **Sequences:** 1(1) **emPAI:** 0.06
unnamed protein product [Mus musculus]

Query	Observed	Mr(expt)	Mr(calc)	Delta	Miss	Score	Expect	Rank	Unique	Peptide
13198	**611.9623**	**1221.9101**	**1221.6162**	**0.2939**	**0**	**56**	**0.0059**	**1**	**U**	**R.LITLEEEMTK.Y**

Proteins matching the same set of peptides:
gi|42415475 **Mass:** 57023 **Score:** 56 **Matches:** 1(1) **Sequences:** 1(1)
protein disulfide-isomerase precursor [Mus musculus]
gi|74141920 **Mass:** 57053 **Score:** 56 **Matches:** 1(1) **Sequences:** 1(1)
unnamed protein product [Mus musculus]
gi|74149659 **Mass:** 43005 **Score:** 56 **Matches:** 1(1) **Sequences:** 1(1)
unnamed protein product [Mus musculus]
gi|74178069 **Mass:** 57099 **Score:** 56 **Matches:** 1(1) **Sequences:** 1(1)
unnamed protein product [Mus musculus]
gi|74190076 **Mass:** 56963 **Score:** 56 **Matches:** 1(1) **Sequences:** 1(1)
unnamed protein product [Mus musculus]
gi|74198312 **Mass:** 57051 **Score:** 56 **Matches:** 1(1) **Sequences:** 1(1)
unnamed protein product [Mus musculus]
gi|74198706 **Mass:** 57000 **Score:** 56 **Matches:** 1(1) **Sequences:** 1(1)
unnamed protein product [Mus musculus]
gi|74203945 **Mass:** 56566 **Score:** 56 **Matches:** 1(1) **Sequences:** 1(1)
unnamed protein product [Mus musculus]
gi|74212231 **Mass:** 57053 **Score:** 56 **Matches:** 1(1) **Sequences:** 1(1)
unnamed protein product [Mus musculus]
gi|74219772 **Mass:** 57008 **Score:** 56 **Matches:** 1(1) **Sequences:** 1(1)
unnamed protein product [Mus musculus]
gi|74220649 **Mass:** 57067 **Score:** 56 **Matches:** 1(1) **Sequences:** 1(1)
unnamed protein product [Mus musculus]
gi|148702818 **Mass:** 58984 **Score:** 56 **Matches:** 1(1) **Sequences:** 1(1)
prolyl 4-hydroxylase, beta polypeptide, isoform CRA_a [Mus musculus]
gi|148702819 **Mass:** 61492 **Score:** 56 **Matches:** 1(1) **Sequences:** 1(1)
prolyl 4-hydroxylase, beta polypeptide, isoform CRA_b [Mus musculus]

---

18. gi|20070408 **Mass:** 113195 **Score:** 55 **Matches:** 1(1) **Sequences:** 1(1) **emPAI:** 0.03

glycine dehydrogenase [decarboxylating], mitochondrial precursor [Mus musculus]

Query	Observed	Mr(expt)	Mr(calc)	Delta	Miss	Score	Expect	Rank	Unique	Peptide
11734	546.4593	1090.9039	1091.4228	-0.5189	0	55	0.0027	1	U	R.DSSGGGGGGGGGDR.G

Proteins matching the same set of peptides:

gi|148709754 **Mass:** 30341 **Score:** 55 **Matches:** 1(1) **Sequences:** 1(1)
glycine decarboxylase, isoform CRA_c [Mus musculus]
gi|148709755 **Mass:** 114180 **Score:** 55 **Matches:** 1(1) **Sequences:** 1(1)
glycine decarboxylase, isoform CRA_d [Mus musculus]
gi|148709756 **Mass:** 98319 **Score:** 55 **Matches:** 1(1) **Sequences:** 1(1)
glycine decarboxylase, isoform CRA_e [Mus musculus]

---

19. gi|3642689 **Mass:** 9181 **Score:** 54 **Matches:** 1(1) **Sequences:** 1(1) **emPAI:** 0.38
immunoglobulin heavy chain-binding protein, partial [Mus musculus]

Query	Observed	Mr(expt)	Mr(calc)	Delta	Miss	Score	Expect	Rank	Unique	Peptide
15298	722.9624	1443.9102	1443.6994	0.2108	0	54	0.0046	1	U	-.TWNDPTVQQDIK.F

---

20. gi|26344548 **Mass:** 58526 **Score:** 54 **Matches:** 1(1) **Sequences:** 1(1) **emPAI:** 0.06
unnamed protein product [Mus musculus]

Query	Observed	Mr(expt)	Mr(calc)	Delta	Miss	Score	Expect	Rank	Unique	Peptide
11185	521.0774	1040.1402	1039.6390	0.5013	0	54	0.003	1	U	K.VLEALQVLR.C

Proteins matching the same set of peptides:

gi|35193112 **Mass:** 55286 **Score:** 54 **Matches:** 1(1) **Sequences:** 1(1)
Wdr26 protein [Mus musculus]
gi|148681181 **Mass:** 58625 **Score:** 54 **Matches:** 1(1) **Sequences:** 1(1)
WD repeat domain 26 [Mus musculus]
gi|264681550 **Mass:** 70499 **Score:** 54 **Matches:** 1(1) **Sequences:** 1(1)
WD repeat-containing protein 26 [Mus musculus]

---

21. gi|836898 **Mass:** 59960 **Score:** 54 **Matches:** 2(1) **Sequences:** 2(1) **emPAI:** 0.11
prolyl 4-hydroxylase alpha(I)-subunit, partial [Mus musculus]

Query	Observed	Mr(expt)	Mr(calc)	Delta	Miss	Score	Expect	Rank	Unique	Peptide
12213	567.0950	1132.1754	1131.5924	0.5830	0	49	0.011	1	U	K.GIAVDYLPER.Q
13453	625.1786	1248.3426	1248.6462	-0.3036	0	43	0.091	1	U	K.LLELDPEHQR.A

Proteins matching the same set of peptides:

gi|26336999 **Mass:** 60848 **Score:** 54 **Matches:** 2(1) **Sequences:** 2(1)
unnamed protein product [Mus musculus]
gi|33859596 **Mass:** 60872 **Score:** 54 **Matches:** 2(1) **Sequences:** 2(1)
prolyl 4-hydroxylase subunit alpha-1 precursor [Mus musculus]
gi|74224984 **Mass:** 60886 **Score:** 54 **Matches:** 2(1) **Sequences:** 2(1)
unnamed protein product [Mus musculus]
gi|74225936 **Mass:** 63769 **Score:** 54 **Matches:** 2(1) **Sequences:** 2(1)
unnamed protein product [Mus musculus]

---

22. gi|4159806 **Mass:** 65183 **Score:** 52 **Matches:** 1(1) **Sequences:** 1(1) **emPAI:** 0.05
type II keratin subunit protein [Mus musculus]

Query	Observed	Mr(expt)	Mr(calc)	Delta	Miss	Score	Expect	Rank	Unique	Peptide
13588	633.1061	1264.1977	1264.6299	-0.4322	0	52	0.01	1	U	R.TNAENEFVTIK.K

Proteins matching the same set of peptides:

gi|12859782 **Mass:** 65586 **Score:** 52 **Matches:** 1(1) **Sequences:** 1(1)
unnamed protein product [Mus musculus]
gi|126116585 **Mass:** 65565 **Score:** 52 **Matches:** 1(1) **Sequences:** 1(1)
keratin, type II cytoskeletal 1 [Mus musculus]

---

23. gi|37935731 **Mass:** 192078 **Score:** 52 **Matches:** 1(1) **Sequences:** 1(1) **emPAI:** 0.02
ATP-binding cassette transporter sub-family A member 16 [Mus musculus]

Query	Observed	Mr(expt)	Mr(calc)	Delta	Miss	Score	Expect	Rank	Unique	Peptide
13521	629.4885	1256.9625	1256.4835	0.4790	0	52	0.012	1	U	R.MEECEALCTR.L

Proteins matching the same set of peptides:
gi|148685261 **Mass:** 167887 **Score:** 52 **Matches:** 1(1) **Sequences:** 1(1)
ATP-binding cassette, sub-family A (ABC1), member 16 [Mus musculus]

24. gi|21595163 **Mass:** 71011 **Score:** 48 **Matches:** 1(1) **Sequences:** 1(1) **emPAI:** 0.05
Glycosyltransferase 25 domain containing 1 [Mus musculus]

Query	Observed	Mr(expt)	Mr(calc)	Delta	Miss	Score	Expect	Rank	Unique	Peptide
9480	435.0096	868.0046	867.5905	0.4140	0	48	0.0098	1	U	R.VLIALLAR.N

Proteins matching the same set of peptides:
gi|148697003 **Mass:** 54889 **Score:** 48 **Matches:** 1(1) **Sequences:** 1(1)
glycosyltransferase 25 domain containing 1, isoform CRA_b [Mus musculus]
gi|170784829 **Mass:** 71015 **Score:** 48 **Matches:** 1(1) **Sequences:** 1(1)
procollagen galactosyltransferase 1 precursor [Mus musculus]
gi|74217150 **Mass:** 70997 **Score:** 48 **Matches:** 1(1) **Sequences:** 1(1)
unnamed protein product [Mus musculus]

25. gi|12847276 **Score:** 48 **Matches:** 1(1) **Sequences:** 1(1) **emPAI:** 0.07
unnamed protein product [Mus musculus]

Query	Observed	Mr(expt)	Mr(calc)	Delta	Miss	Score	Expect	Rank	Unique	Peptide
15600	739.0794	1476.1442	1475.7442	0.4000	1	48	0.013	3	U	R.YEPKCPLGVDISR.E

Proteins matching the same set of peptides:
gi|31560081 **Score:** 48 **Matches:** 1(1) **Sequences:** 1(1)
gi|148667576 **Score:** 48 **Matches:** 1(1) **Sequences:** 1(1)

26. gi|74191500 **Mass:** 57022 **Score:** 46 **Matches:** 1(1) **Sequences:** 1(1) **emPAI:** 0.06
unnamed protein product [Mus musculus]

Query	Observed	Mr(expt)	Mr(calc)	Delta	Miss	Score	Expect	Rank	Unique	Peptide
13198	611.9623	1221.9101	1220.6686	1.2415	1	46	0.05	2	U	R.LITLEEKMTK.Y

27. gi|81875970 **Mass:** 71638 **Score:** 45 **Matches:** 1(0) **Sequences:** 1(0) **emPAI:** 0.05
RecName: Full=Alkyldihydroxyacetonephosphate synthase, peroxisomal; Short=Alkyl-DHAP synthase; AltName: Full=Alkylglycerone-phosphate synthase; Flags: Precursor

Query	Observed	Mr(expt)	Mr(calc)	Delta	Miss	Score	Expect	Rank	Unique	Peptide
15186	717.2085	2148.6037	2149.1127	-0.5090	1	45	0.06	1	U	R.RAASAAGASPAATPAAPESGTIPK.K

Proteins matching the same set of peptides:
gi|148695250 **Mass:** 53219 **Score:** 45 **Matches:** 1(0) **Sequences:** 1(0)
alkylglycerone phosphate synthase, isoform CRA_a [Mus musculus]
gi|295444834 **Mass:** 74296 **Score:** 45 **Matches:** 1(0) **Sequences:** 1(0)
alkyldihydroxyacetonephosphate synthase, peroxisomal [Mus musculus]

28. gi|7513694 **Mass:** 109137 **Score:** 44 **Matches:** 1(0) **Sequences:** 1(0) **emPAI:** 0.03
IgG Fc binding protein - mouse (fragment)

Query	Observed	Mr(expt)	Mr(calc)	Delta	Miss	Score	Expect	Rank	Unique	Peptide
9539	874.3927	873.3854	873.4920	-0.1066	0	44	0.06	1	U	R.ISVINGGSK.A

Proteins matching the same set of peptides:
gi|148692210 **Mass:** 147962 **Score:** 44 **Matches:** 1(0) **Sequences:** 1(0)
mCG145390, isoform CRA_a [Mus musculus]
gi|148692211 **Mass:** 146729 **Score:** 44 **Matches:** 1(0) **Sequences:** 1(0)

mCG145390, isoform CRA_b [Mus musculus]
gi|169790797 **Mass:** 275058 **Score:** 44 **Matches:** 1(0) **Sequences:** 1(0)
Fc fragment of IgG binding protein precursor [Mus musculus]
gi|257467625 **Mass:** 280044 **Score:** 44 **Matches:** 1(0) **Sequences:** 1(0)
Fc fragment of IgG binding protein-like precursor [Mus musculus]

---

29. gi|148664969 **Mass:** 26715 **Score:** 44 **Matches:** 1(1) **Sequences:** 1(1) **emPAI:** 0.12
RIKEN cDNA 2900011O08, isoform CRA_a [Mus musculus]

Query	Observed	Mr(expt)	Mr(calc)	Delta	Miss	Score	Expect	Rank	Unique	Peptide
12819	593.2423	1184.4701	1185.5812	-1.1111	0	44	0.053	1	U	-.NTADLSCLPPR.S

---

30. gi|12855410 **Mass:** 22073 **Score:** 43 **Matches:** 1(1) **Sequences:** 1(1) **emPAI:** 0.15
unnamed protein product [Mus musculus]

Query	Observed	Mr(expt)	Mr(calc)	Delta	Miss	Score	Expect	Rank	Unique	Peptide
13489	627.3521	1252.6896	1252.6526	0.0370	1	43	0.056	1	U	R.EKMVVFFPPK.E

Mascot: http://www.matrixscience.com/

Mascot Search Results

PFHR9

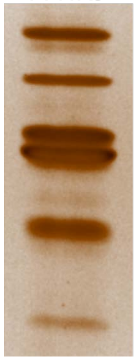

3

User	:		
Email	:		
Search title	:		
MS data file	:	ZIEN-29Nov2017_3m.mgf	
Database	:	NCBInr 20120419 (17893860 sequences; 6141683785 residues)	
Taxonomy	:	Mus musculus (house mouse) (138831 sequences)	
Timestamp	:	30 Nov 2017 at 20:39:15 GMT	
Enzyme	:	Trypsin	
Variable modifications	:	Carbamidomethyl (C),Oxidation (M),Oxidation (P)	
Mass values	:	Monoisotopic	
Protein Mass	:	Unrestricted	
Peptide Mass Tolerance	:	± 1.25 Da	
Fragment Mass Tolerance	:	± 1.001 Da	
Max Missed Cleavages	:	1	
Instrument type	:	Default	
Number of queries	:	19332	
Protein hits	:	gi|54777	unnamed protein product [Mus musculus]
		gi|148701597	procollagen-proline, 2-oxoglutarate 4-dioxygenase (proline 4-hydroxylase), alpha II polypeptide, isoform CRA_b [Mus musculus]
		gi|148701600	procollagen-proline, 2-oxoglutarate 4-dioxygenase (proline 4-hydroxylase), alpha II polypeptide, isoform CRA_e [Mus musculus]
		gi|74225936	unnamed protein product [Mus musculus]
		gi|16716569	protease, serine, 1 precursor [Mus musculus]
		gi|347839	matricin [Mus musculus]
		gi|21312570	protein ERGIC-53 precursor [Mus musculus]
		gi|387398	epidermal keratin type I, partial [Mus musculus]
		gi|51452	unnamed protein product [Mus musculus]
		gi|74213832	unnamed protein product [Mus musculus]
		gi|22164776	keratin, type II cytoskeletal 79 [Mus musculus]
		gi|12843914	unnamed protein product [Mus musculus]
		gi|5295992	chaperonin containing TCP-1 theta subunit [Mus musculus]
		gi|1419012	CCT zeta2, zeta2 subunit of the chaperonin containing TCP-1 (CCT) [Mus musculus]
		gi|7710044	kelch-like ECH-associated protein 1 [Mus musculus]
		gi|2623222	ATP synthase beta-subunit [Mus musculus]
		gi|192262	pro-alpha-1 type I collagen, partial [Mus musculus]
		gi|551295	pyruvate kinase M [Mus musculus]
		gi|52789	unnamed protein product [Mus musculus]
		gi|46485130	TPA_exp: keratin Kb40 [Mus musculus]
		gi|1304157	78 kDa glucose-regulated protein [Mus musculus]
		gi|4159806	type II keratin subunit protein [Mus musculus]
		gi|293686	epidermal keratin subunit II [Mus musculus]
		gi|3687320	cartilage-associated protein (CASP) [Mus musculus]
		gi|74203983	unnamed protein product [Mus musculus]
		gi|468546	CCT (chaperonin containing TCP-1) beta subunit [Mus musculus]
		gi|22297268	AID, partial [Homo sapiens]
		gi|51450	heat shock protein [Mus musculus]
		gi|6680748	ATP synthase subunit alpha, mitochondrial precursor [Mus musculus]
		gi|148688671	mCG16097 [Mus musculus]
		gi|6755893	trypsin 4 precursor [Mus musculus]
		gi|148707748	nuclear casein kinase and cyclin-dependent kinase substrate 1, isoform CRA_b [Mus musculus]
		gi|53830774	lactase [Mus musculus]
		gi|26324736	unnamed protein product [Mus musculus]
		gi|7513694	IgG Fc binding protein - mouse (fragment)
		gi|111599858	Dehydrogenase E1 and transketolase domain containing 1 [Mus musculus]
		gi|74224513	unnamed protein product [Mus musculus]
		gi|21410133	Kidins220 protein [Mus musculus]
		gi|26333649	unnamed protein product [Mus musculus]
		gi|986918	A10 [Mus musculus]
		gi|21313084	chromodomain Y-like protein 2 [Mus musculus]
		gi|124504325	Armcx4 protein [Mus musculus]
		gi|12843046	unnamed protein product [Mus musculus]
		gi|12836661	unnamed protein product [Mus musculus]

gi|148681245 mCG145199 [Mus musculus]
gi|148667455 TEA domain family member 4, isoform CRA_b [Mus musculus]
gi|642072 fibrillin-1, partial [Mus musculus]
gi|12836791 unnamed protein product [Mus musculus]
gi|14250408 Aspartyl-tRNA synthetase [Mus musculus]
gi|17512475 Ankyrin repeat and SOCS box-containing 6 [Mus musculus]
gi|309271872 PREDICTED: zinc finger protein 469 [Mus musculus]
gi|26380166 unnamed protein product [Mus musculus]

Select Summary Report

Format As | Select Summary (protein hits) | Help

Significance threshold p< 0.05 Max. number of hits AUTO

Standard scoring ○ MudPIT scoring ◉ Ions score or expect cut-off 42 Show sub-sets 0

Show pop-ups ◉ Suppress pop-ups ○ Require bold red ☐

Re-Search ◉ All queries ○ Unassigned ○ Below homology threshold ○ Below identity threshold

1. gi|54777 **Mass:** 57108 **Score:** 426 **Matches:** 28(25) **Sequences:** 16(15) **emPAI:** 1.90
unnamed protein product [Mus musculus]

Query	Observed	Mr(expt)	Mr(calc)	Delta	Miss	Score	Expect	Rank	Unique	Peptide
10277	381.1882	760.3618	760.3715	-0.0097	0	50	0.015	1	U	K.AEGSEIR.L
10911	395.3300	788.6454	788.3664	0.2790	0	59	0.0018	1	U	K.NGDTASPK.E
13577	483.6877	965.3609	965.5586	-0.1977	0	55	0.0037	1	U	R.ILEFFGLK.K
14327	1051.4263	1050.4190	1050.4982	-0.0791	0	51	0.0088	1	U	R.NNFEGEITK.E
14499	1067.7178	1066.7105	1065.5091	1.2014	0	45	0.033	1	U	R.TVIDYNGER.T
14641	1081.6606	1080.6533	1080.6695	-0.0162	0	52	0.007	1	U	K.THILLFLPK.S 14649
14648	541.6124	1081.2102	1080.6695	0.5406	0	(48)	0.012	1	U	K.THILLFLPK.S 14646
16005	1203.5746	1202.5673	1201.5979	0.9694	0	49	0.017	1	U	R.EADDIVNWLK.K
16043	604.0506	1206.0866	1205.6213	0.4653	0	51	0.0064	1	U	R.LITLEEEMTK.Y 16046
16166	608.9402	1215.8658	1215.5884	0.2775	0	47	0.025	1	U	K.SNFEEALAAHK.Y
16232	611.6342	1221.2538	1221.6162	-0.3625	0	(44)	0.088	1	U	R.LITLEEEMTK.Y
16991	647.9220	1293.8294	1292.5918	1.2376	0	84	4.2e-006	1	U	K.MDSTANEVEAVK.V 16980 16985 16986 16990
17115	654.5397	1307.0648	1307.6357	-0.5709	1	46	0.043	1	U	R.NNFEGEITKEK.L
18095	475.6379	1423.8917	1423.7711	0.1206	1	(46)	0.031	1	U	K.YQLDKDGVVLFK.K
18099	713.1129	1424.2113	1423.7711	0.4402	1	55	0.0069	1	U	K.YQLDKDGVVLFK.K 18100
18159	717.4308	1432.8470	1431.6882	1.1589	1	63	0.0011	1	U	K.SVSDYDGKLSSFK.R
15815	594.4030	1780.1872	1779.8275	0.3597	0	(61)	0.0019	1	U	K.VDATEESDLAQQYGVR.G
19294	891.2250	1780.4354	1779.8275	0.6079	0	78	2.7e-005	1	U	K.VDATEESDLAQQYGVR.G
16231	611.6050	1831.7933	1832.9057	-1.1124	0	43	0.1	1	U	K.ILFIFIDSDHTDNQR.I
19326	1493.5814	2985.1482	2984.4866	0.6616	0	98	2.4e-007	1	U	R.TGPAATTLSDTAAAESLVDSSEVTVIGFFK.D

Proteins matching the same set of peptides:
gi|74212231 **Mass:** 57053 **Score:** 426 **Matches:** 28(25) **Sequences:** 16(15)
unnamed protein product [Mus musculus]
gi|74190076 **Mass:** 56963 **Score:** 426 **Matches:** 28(25) **Sequences:** 16(15)
unnamed protein product [Mus musculus]
gi|42415475 **Mass:** 57023 **Score:** 426 **Matches:** 28(25) **Sequences:** 16(15)
protein disulfide-isomerase precursor [Mus musculus]
gi|74141920 **Mass:** 57053 **Score:** 426 **Matches:** 28(25) **Sequences:** 16(15)
unnamed protein product [Mus musculus]

gi|74178069 **Mass:** 57099 **Score:** 426 **Matches:** 28(25) **Sequences:** 16(15)
unnamed protein product [Mus musculus]
gi|74198312 **Mass:** 57051 **Score:** 426 **Matches:** 28(25) **Sequences:** 16(15)
unnamed protein product [Mus musculus]
gi|74198706 **Mass:** 57000 **Score:** 426 **Matches:** 28(25) **Sequences:** 16(15)
unnamed protein product [Mus musculus]
gi|74219772 **Mass:** 57008 **Score:** 426 **Matches:** 28(25) **Sequences:** 16(15)
unnamed protein product [Mus musculus]
gi|148702818 **Mass:** 58984 **Score:** 426 **Matches:** 28(25) **Sequences:** 16(15)
prolyl 4-hydroxylase, beta polypeptide, isoform CRA_a [Mus musculus]
gi|148702819 **Mass:** 61492 **Score:** 426 **Matches:** 28(25) **Sequences:** 16(15)
prolyl 4-hydroxylase, beta polypeptide, isoform CRA_b [Mus musculus]

---

2. gi|148701597 **Mass:** 57417 **Score:** 223 **Matches:** 17(15) **Sequences:** 10(10) **emPAI:** 0.75
procollagen-proline, 2-oxoglutarate 4-dioxygenase (proline 4-hydroxylase), alpha II polypeptide, isoform CRA_b [Mus musculus]

Query	Observed	Mr(expt)	Mr(calc)	Delta	Miss	Score	Expect	Rank	Unique	Peptide
13332	470.4389	938.8633	938.3981	0.4651	0	51	0.006	1	U	R.SDEQDAFK.R
14757	1093.6360	1092.6287	1092.5703	0.0585	0	44	0.054	1		K.EYILVEEAK.L 14753
14783	548.5247	1095.0348	1094.4992	0.5356	1	44	0.031	1	U	R.SDEQDAFKR.L
15163	1125.7443	1124.7370	1124.6958	0.0413	0	46	0.028	1		R.VPQLLIAPFK.E
15611	583.9658	1165.9171	1165.6091	0.3080	0	48	0.013	1		R.LLSLDPSHER.A
16687	631.0879	1260.1612	1260.6198	-0.4585	0	68	0.00026	1		K.QLDAGEEATVTK.S 16695 16699
18396	734.0641	1466.1136	1466.8034	-0.6898	1	59	0.0027	1		K.KGTAVFWYNLLR.S 18398
18833	774.6111	1547.2077	1547.6450	-0.4372	0	55	0.0061	1		R.YYDVMSDEEIER.I 18840 18842
19065	805.0440	1608.0735	1607.7250	0.3485	0	47	0.046	1		R.GQEFLRPCGTTEVD.- 19066
19092	809.5747	1617.1349	1616.7318	0.4030	0	69	0.00021	1		K.SSWLEEDDDPVVAR.V

Proteins matching the same set of peptides:
gi|226874876 **Mass:** 60977 **Score:** 223 **Matches:** 17(15) **Sequences:** 10(10)
prolyl 4-hydroxylase subunit alpha-2 isoform 2 precursor [Mus musculus]

---

3. gi|148701600 **Mass:** 66870 **Score:** 216 **Matches:** 16(14) **Sequences:** 9(9) **emPAI:** 0.54
procollagen-proline, 2-oxoglutarate 4-dioxygenase (proline 4-hydroxylase), alpha II polypeptide, isoform CRA_e [Mus musculus]

Query	Observed	Mr(expt)	Mr(calc)	Delta	Miss	Score	Expect	Rank	Unique	Peptide
13143	460.4962	918.9779	918.4923	0.4856	0	47	0.016	1	U	R.RPFDSGLK.T
14757	1093.6360	1092.6287	1092.5703	0.0585	0	44	0.054	1		K.EYILVEEAK.L 14753
15163	1125.7443	1124.7370	1124.6958	0.0413	0	46	0.028	1		R.VPQLLIAPFK.E
15611	583.9658	1165.9171	1165.6091	0.3080	0	48	0.013	1		R.LLSLDPSHER.A
16687	631.0879	1260.1612	1260.6198	-0.4585	0	68	0.00026	1		K.QLDAGEEATVTK.S 16695 16699
18396	734.0641	1466.1136	1466.8034	-0.6898	1	59	0.0027	1		K.KGTAVFWYNLLR.S 18398
18833	774.6111	1547.2077	1547.6450	-0.4372	0	55	0.0061	1		R.YYDVMSDEEIER.I 18840 18842
19065	805.0440	1608.0735	1607.7250	0.3485	0	47	0.046	1		R.GQEFLRPCGTTEVD.- 19066
19092	809.5747	1617.1349	1616.7318	0.4030	0	69	0.00021	1		K.SSWLEEDDDPVVAR.V

Proteins matching the same set of peptides:
gi|209862961 **Mass:** 60774 **Score:** 216 **Matches:** 16(14) **Sequences:** 9(9)
prolyl 4-hydroxylase subunit alpha-2 isoform 1 precursor [Mus musculus]

---

4. gi|74225936 **Mass:** 63769 **Score:** 160 **Matches:** 10(8) **Sequences:** 8(6) **emPAI:** 0.42
unnamed protein product [Mus musculus]

Query	Observed	Mr(expt)	Mr(calc)	Delta	Miss	Score	Expect	Rank	Unique	Peptide
13568	483.3983	964.7821	964.5090	0.2731	0	50	0.0085	1	U	K.GNLPGVQHK.S
14305	524.3110	1046.6075	1045.5590	1.0485	0	44	0.11	1	U	R.RPCTLSELK.S
15244	1132.6241	1131.6168	1131.5924	0.0244	0	44	0.05	1	U	K.GIAVDYLPER.Q
15607	583.4688	1164.9231	1164.6074	0.3157	0	68	0.00014	1	U	R.HAACPVLVGNK.W
16994	648.0558	1294.0970	1293.7180	0.3790	1	47	0.017	1	U	K.DLVTSLKDYIK.A
18891	783.3275	1564.6405	1564.7522	-0.1117	0	53	0.013	1	U	K.SAWLSGYEDPVVSR.I
17262	662.6197	1984.8374	1984.0490	0.7884	1	44	0.076	1	U	R.RATISNPVTGALETVHYR.I
18487	739.6022	2215.7847	2215.1274	0.6573	1	58	0.0028	1	U	R.LTSTATKDPEGFVGHPVNAFK.L 18476 18483

---

5. gi|16716569 **Mass:** 26118 **Score:** 136 **Matches:** 5(4) **Sequences:** 1(1) **emPAI:** 0.27
protease, serine, 1 precursor [Mus musculus]

Query	Observed	Mr(expt)	Mr(calc)	Delta	Miss	Score	Expect	Rank	Unique	Peptide
18449	737.5288	2209.5646	2210.0967	-0.5321	0	(67)	0.00036	1	U	R.LGEHNINVLEGNEQFIDAAK.I 18450 18452 18459
19321	1106.1886	2210.3626	2210.0967	0.2659	0	78	2.9e-005	1	U	R.LGEHNINVLEGNEQFIDAAK.I

---

6. gi|347839 **Mass:** 60450 **Score:** 101 **Matches:** 2(2) **Sequences:** 2(2) **emPAI:** 0.11
matricin [Mus musculus]

Query	Observed	Mr(expt)	Mr(calc)	Delta	Miss	Score	Expect	Rank	Unique	Peptide
15211	565.4988	1128.9830	1128.6251	0.3579	1	73	4.7e-005	1	U	R.KVQSGNINAAK.T
16416	619.2510	1236.4874	1236.6094	-0.1220	0	70	0.00023	1	U	R.MALDDMISTLK.K

Proteins matching the same set of peptides:
gi|6753320 **Mass:** 60591 **Score:** 101 **Matches:** 2(2) **Sequences:** 2(2)
T-complex protein 1 subunit gamma [Mus musculus]
gi|74142612 **Mass:** 58241 **Score:** 101 **Matches:** 2(2) **Sequences:** 2(2)
unnamed protein product [Mus musculus]
gi|74226937 **Mass:** 60563 **Score:** 101 **Matches:** 2(2) **Sequences:** 2(2)
unnamed protein product [Mus musculus]

---

7. gi|21312570 **Mass:** 57753 **Score:** 91 **Matches:** 3(3) **Sequences:** 2(2) **emPAI:** 0.12
protein ERGIC-53 precursor [Mus musculus]

Query	Observed	Mr(expt)	Mr(calc)	Delta	Miss	Score	Expect	Rank	Unique	Peptide
16899	642.4886	1282.9626	1282.6405	0.3222	0	66	0.00044	1	U	R.YVSSLTEEISR.R 16889
18234	723.1138	2166.3195	2165.9978	0.3217	0	50	0.019	1	U	K.GHPDLQGQPADDIFESIGDR.E

Proteins matching the same set of peptides:
gi|74152119 **Mass:** 61236 **Score:** 91 **Matches:** 3(3) **Sequences:** 2(2)
unnamed protein product [Mus musculus]

---

8. gi|387398 **Mass:** 41454 **Score:** 83 **Matches:** 2(2) **Sequences:** 1(1) **emPAI:** 0.08
epidermal keratin type I, partial [Mus musculus]

Query	Observed	Mr(expt)	Mr(calc)	Delta	Miss	Score	Expect	Rank	Unique	Peptide
14151	515.5348	1029.0550	1028.5866	0.4684	0	66	0.00022	1	U	R.VLDELTLAR.A 14152

Proteins matching the same set of peptides:
gi|741022 **Mass:** 49086 **Score:** 83 **Matches:** 2(2) **Sequences:** 1(1)
keratin 15
gi|904215 **Mass:** 49129 **Score:** 83 **Matches:** 2(2) **Sequences:** 1(1)

cytokeratin 15 [Mus musculus]
gi|1083398 **Mass:** 7958 **Score:** 83 **Matches:** 2(2) **Sequences:** 1(1)
keratin 15, type I, cytoskeletal - mouse (fragments)
gi|6680606 **Mass:** 44515 **Score:** 83 **Matches:** 2(2) **Sequences:** 1(1)
keratin, type I cytoskeletal 19 [Mus musculus]
gi|7106335 **Mass:** 48132 **Score:** 83 **Matches:** 2(2) **Sequences:** 1(1)
keratin, type I cytoskeletal 17 [Mus musculus]
gi|13097093 **Mass:** 30629 **Score:** 83 **Matches:** 2(2) **Sequences:** 1(1)
Krt14 protein [Mus musculus]
gi|21489935 **Mass:** 52834 **Score:** 83 **Matches:** 2(2) **Sequences:** 1(1)
keratin, type I cytoskeletal 14 [Mus musculus]
gi|38503465 **Mass:** 50146 **Score:** 83 **Matches:** 2(2) **Sequences:** 1(1)
keratin 17n [Mus musculus]
gi|83304265 **Mass:** 49107 **Score:** 83 **Matches:** 2(2) **Sequences:** 1(1)
RecName: Full=Keratin, type I cytoskeletal 15; AltName: Full=Cytokeratin-15; Short=CK-15; AltName: Full=Keratin-15; Short=K15
gi|85726518 **Mass:** 49857 **Score:** 83 **Matches:** 2(2) **Sequences:** 1(1)
Krt42 protein [Mus musculus]
gi|148670626 **Mass:** 54668 **Score:** 83 **Matches:** 2(2) **Sequences:** 1(1)
mCG144006 [Mus musculus]
gi|154090941 **Mass:** 50102 **Score:** 83 **Matches:** 2(2) **Sequences:** 1(1)
keratin, type I cytoskeletal 42 [Mus musculus]
gi|156139032 **Mass:** 50162 **Score:** 83 **Matches:** 2(2) **Sequences:** 1(1)
Keratin 42 [Mus musculus]
gi|226823220 **Mass:** 49463 **Score:** 83 **Matches:** 2(2) **Sequences:** 1(1)
keratin, type I cytoskeletal 15 [Mus musculus]

---

9. gi|51452 **Mass:** 58833 **Score:** 66 **Matches:** 2(2) **Sequences:** 2(2) **emPAI:** 0.11
unnamed protein product [Mus musculus]

Query	Observed	Mr(expt)	Mr(calc)	Delta	Miss	Score	Expect	Rank	Unique	Peptide
17855	695.5581	1389.1017	1388.6976	0.4041	0	52	0.012	1	U	R.GYISPYFINTSK.G
18659	752.6903	1503.3661	1503.7490	-0.3830	0	53	0.011	1	U	K.TLNDELEIIEGMK.F

Proteins matching the same set of peptides:
gi|51455 **Mass:** 60903 **Score:** 66 **Matches:** 2(2) **Sequences:** 2(2)
heat shock protein 65 [Mus musculus]
gi|26353954 **Mass:** 60918 **Score:** 66 **Matches:** 2(2) **Sequences:** 2(2)
unnamed protein product [Mus musculus]
gi|76779273 **Mass:** 59388 **Score:** 66 **Matches:** 2(2) **Sequences:** 2(2)
Hspd1 protein, partial [Mus musculus]
gi|183396771 **Mass:** 60917 **Score:** 66 **Matches:** 2(2) **Sequences:** 2(2)
60 kDa heat shock protein, mitochondrial [Mus musculus]

---

10. gi|74213832 **Mass:** 52871 **Score:** 65 **Matches:** 1(1) **Sequences:** 1(1) **emPAI:** 0.06
unnamed protein product [Mus musculus]

Query	Observed	Mr(expt)	Mr(calc)	Delta	Miss	Score	Expect	Rank	Unique	Peptide
17796	692.4768	1382.9391	1382.5772	0.3618	0	65	0.00055	1	U	R.GGAEQFMEETER.S

Proteins matching the same set of peptides:
gi|148666718 **Mass:** 43932 **Score:** 65 **Matches:** 1(1) **Sequences:** 1(1)
chaperonin subunit 7 (eta), isoform CRA_c [Mus musculus]
gi|26346713 **Mass:** 59598 **Score:** 65 **Matches:** 1(1) **Sequences:** 1(1)

unnamed protein product [Mus musculus]
gi|74147234 **Mass:** 59610 **Score:** 65 **Matches:** 1(1) **Sequences:** 1(1)
unnamed protein product [Mus musculus]
gi|74151643 **Mass:** 59646 **Score:** 65 **Matches:** 1(1) **Sequences:** 1(1)
unnamed protein product [Mus musculus]
gi|74191272 **Mass:** 59600 **Score:** 65 **Matches:** 1(1) **Sequences:** 1(1)
unnamed protein product [Mus musculus]
gi|74212111 **Mass:** 59614 **Score:** 65 **Matches:** 1(1) **Sequences:** 1(1)
unnamed protein product [Mus musculus]
gi|148666717 **Mass:** 61343 **Score:** 65 **Matches:** 1(1) **Sequences:** 1(1)
chaperonin subunit 7 (eta), isoform CRA_b [Mus musculus]
gi|238814391 **Mass:** 59614 **Score:** 65 **Matches:** 1(1) **Sequences:** 1(1)
T-complex protein 1 subunit eta [Mus musculus]

---

11. gi|22164776 **Mass:** 57517 **Score:** 65 **Matches:** 2(1) **Sequences:** 2(1) **emPAI:** 0.06
keratin, type II cytoskeletal 79 [Mus musculus]

Query	Observed	Mr(expt)	Mr(calc)	Delta	Miss	Score	Expect	Rank	Unique	Peptide
16899	642.4886	1282.9626	1283.5491	-0.5864	0	43	0.084	2	U	K.GGFSSNSASGGGGSR.M
17315	**665.6135**	**1329.2125**	**1328.7187**	**0.4938**	**0**	**65**	**0.00064**	**1**	**U**	**R.NLDLDSIIAEVK.A**

Proteins matching the same set of peptides:
gi|148672069 **Mass:** 59231 **Score:** 65 **Matches:** 2(1) **Sequences:** 2(1)
cDNA sequence BC031593 [Mus musculus]

---

12. gi|12843914 **Mass:** 29993 **Score:** 63 **Matches:** 2(2) **Sequences:** 1(1) **emPAI:** 0.11
unnamed protein product [Mus musculus]

Query	Observed	Mr(expt)	Mr(calc)	Delta	Miss	Score	Expect	Rank	Unique	Peptide
17066	**652.3516**	**1302.6886**	**1301.7078**	**0.9807**	**0**	**56**	**0.0062**	**1**	**U**	**R.SLDLDSIIAEVK.A** 17059

Proteins matching the same set of peptides:
gi|16303309 **Mass:** 61743 **Score:** 63 **Matches:** 2(2) **Sequences:** 1(1)
type II keratin 5 [Mus musculus]
gi|20911031 **Mass:** 61729 **Score:** 63 **Matches:** 2(2) **Sequences:** 1(1)
keratin, type II cytoskeletal 5 [Mus musculus]
gi|54607171 **Mass:** 59299 **Score:** 63 **Matches:** 2(2) **Sequences:** 1(1)
keratin, type II cytoskeletal 6A [Mus musculus]
gi|59798479 **Mass:** 60285 **Score:** 63 **Matches:** 2(2) **Sequences:** 1(1)
RecName: Full=Keratin, type II cytoskeletal 6B; AltName: Full=Cytokeratin-6B; Short=CK-6B; AltName: Full=Keratin-6-beta; Short=mK6-beta; AltName: Full=Keratin-6B; Short=K6B
gi|110645788 **Mass:** 60236 **Score:** 63 **Matches:** 2(2) **Sequences:** 1(1)
Krt6b protein [Mus musculus]
gi|113195684 **Mass:** 59490 **Score:** 63 **Matches:** 2(2) **Sequences:** 1(1)
keratin, type II cytoskeletal 6B [Mus musculus]
gi|116063325 **Mass:** 60154 **Score:** 63 **Matches:** 2(2) **Sequences:** 1(1)
Krt6b protein [Mus musculus]
gi|148672084 **Mass:** 34775 **Score:** 63 **Matches:** 2(2) **Sequences:** 1(1)
mCG17577 [Mus musculus]
gi|148672085 **Mass:** 38119 **Score:** 63 **Matches:** 2(2) **Sequences:** 1(1)
mCG144996 [Mus musculus]
gi|13272554 **Mass:** 42357 **Score:** 63 **Matches:** 2(2) **Sequences:** 1(1)
cytokeratin KRT2-6HF [Mus musculus]
gi|29789317 **Mass:** 59704 **Score:** 63 **Matches:** 2(2) **Sequences:** 1(1)

keratin, type II cytoskeletal 75 [Mus musculus]
gi|148672089 **Mass:** 62241 **Score:** 63 **Matches:** 2(2) **Sequences:** 1(1)
keratin 75 [Mus musculus]

---

13. gi|5295992 **Mass:** 59531 **Score:** 63 **Matches:** 2(2) **Sequences:** 2(2) **emPAI:** 0.11
chaperonin containing TCP-1 theta subunit [Mus musculus]

Query	Observed	Mr(expt)	Mr(calc)	Delta	Miss	Score	Expect	Rank	Unique	Peptide
13522	481.9923	961.9701	961.5055	0.4646	0	52	0.0053	1	U	K.APGFAQMLK.D
15269	567.3992	1132.7838	1131.5772	1.2066	0	53	0.012	1	U	R.DVDEVSSLLR.T

Proteins matching the same set of peptides:
gi|50510319 **Mass:** 60190 **Score:** 63 **Matches:** 2(2) **Sequences:** 2(2)
mKIAA0002 protein [Mus musculus]
gi|74219231 **Mass:** 59518 **Score:** 63 **Matches:** 2(2) **Sequences:** 2(2)
unnamed protein product [Mus musculus]
gi|126723461 **Mass:** 59518 **Score:** 63 **Matches:** 2(2) **Sequences:** 2(2)
T-complex protein 1 subunit theta [Mus musculus]

---

14. gi|1419012 **Mass:** 58147 **Score:** 63 **Matches:** 1(1) **Sequences:** 1(1) **emPAI:** 0.06
CCT zeta2, zeta2 subunit of the chaperonin containing TCP-1 (CCT) [Mus musculus]

Query	Observed	Mr(expt)	Mr(calc)	Delta	Miss	Score	Expect	Rank	Unique	Peptide
13828	496.0008	989.9870	989.5216	0.4655	0	63	0.00052	1	U	K.MLVSGAGDIK.L

Proteins matching the same set of peptides:
gi|6753324 **Mass:** 57968 **Score:** 63 **Matches:** 1(1) **Sequences:** 1(1)
T-complex protein 1 subunit zeta [Mus musculus]
gi|62948125 **Mass:** 58040 **Score:** 63 **Matches:** 1(1) **Sequences:** 1(1)
Chaperonin containing Tcp1, subunit 6a (zeta) [Mus musculus]
gi|74198471 **Mass:** 58067 **Score:** 63 **Matches:** 1(1) **Sequences:** 1(1)
unnamed protein product [Mus musculus]
gi|74204475 **Mass:** 57967 **Score:** 63 **Matches:** 1(1) **Sequences:** 1(1)
unnamed protein product [Mus musculus]
gi|74204595 **Mass:** 58068 **Score:** 63 **Matches:** 1(1) **Sequences:** 1(1)
unnamed protein product [Mus musculus]
gi|226693361 **Mass:** 58148 **Score:** 63 **Matches:** 1(1) **Sequences:** 1(1)
T-complex protein 1 subunit zeta-2 [Mus musculus]

---

15. gi|7710044 **Mass:** 69508 **Score:** 60 **Matches:** 1(1) **Sequences:** 1(1) **emPAI:** 0.05
kelch-like ECH-associated protein 1 [Mus musculus]

Query	Observed	Mr(expt)	Mr(calc)	Delta	Miss	Score	Expect	Rank	Unique	Peptide
17782	691.6884	1381.3623	1381.6990	-0.3367	0	60	0.0021	1	U	R.LLYAVGGFDGTNR.L

Proteins matching the same set of peptides:
gi|26337871 **Mass:** 69478 **Score:** 60 **Matches:** 1(1) **Sequences:** 1(1)
unnamed protein product [Mus musculus]
gi|37359786 **Mass:** 71015 **Score:** 60 **Matches:** 1(1) **Sequences:** 1(1)
mKIAA0132 protein [Mus musculus]
gi|74181739 **Mass:** 69492 **Score:** 60 **Matches:** 1(1) **Sequences:** 1(1)
unnamed protein product [Mus musculus]
gi|74200263 **Mass:** 43846 **Score:** 60 **Matches:** 1(1) **Sequences:** 1(1)

unnamed protein product [Mus musculus]
gi|74212473 **Mass:** 69482 **Score:** 60 **Matches:** 1(1) **Sequences:** 1(1)
unnamed protein product [Mus musculus]
gi|93278448 **Mass:** 34652 **Score:** 60 **Matches:** 1(1) **Sequences:** 1(1)
Chain A, Structural Basis For The Defects Of Human Lung Cancer Somatic Mutations In The Repression Activity Of Keap1 On Nrf2
gi|158428176 **Mass:** 34946 **Score:** 60 **Matches:** 1(1) **Sequences:** 1(1)
Chain A, Crystal Structure Of The Keap1 Protein In Complexed With The N-Terminal Region Of The Nrf2 Transcription Factor

---

16. gi|2623222 **Mass:** 56344 **Score:** 60 **Matches:** 1(1) **Sequences:** 1(1) **emPAI:** 0.06
ATP synthase beta-subunit [Mus musculus]

Query	Observed	Mr(expt)	Mr(calc)	Delta	Miss	Score	Expect	Rank	Unique	Peptide
18179	718.5294	1435.0442	1434.7467	0.2975	0	60	0.00091	1	U	R.FTQAGSEVSALLGR.I

Proteins matching the same set of peptides:
gi|23272966 **Mass:** 56632 **Score:** 60 **Matches:** 1(1) **Sequences:** 1(1)
Atp5b protein [Mus musculus]
gi|31980648 **Mass:** 56265 **Score:** 60 **Matches:** 1(1) **Sequences:** 1(1)
ATP synthase subunit beta, mitochondrial precursor [Mus musculus]
gi|74139650 **Mass:** 56207 **Score:** 60 **Matches:** 1(1) **Sequences:** 1(1)
unnamed protein product [Mus musculus]
gi|74151565 **Mass:** 56294 **Score:** 60 **Matches:** 1(1) **Sequences:** 1(1)
unnamed protein product [Mus musculus]
gi|74185232 **Mass:** 56278 **Score:** 60 **Matches:** 1(1) **Sequences:** 1(1)
unnamed protein product [Mus musculus]
gi|74197074 **Mass:** 56294 **Score:** 60 **Matches:** 1(1) **Sequences:** 1(1)
unnamed protein product [Mus musculus]
gi|74198645 **Mass:** 56294 **Score:** 60 **Matches:** 1(1) **Sequences:** 1(1)
unnamed protein product [Mus musculus]
gi|74204509 **Mass:** 56294 **Score:** 60 **Matches:** 1(1) **Sequences:** 1(1)
unnamed protein product [Mus musculus]
gi|74220566 **Mass:** 56221 **Score:** 60 **Matches:** 1(1) **Sequences:** 1(1)
unnamed protein product [Mus musculus]
gi|74225421 **Mass:** 56235 **Score:** 60 **Matches:** 1(1) **Sequences:** 1(1)
unnamed protein product [Mus musculus]
gi|89574015 **Mass:** 48047 **Score:** 60 **Matches:** 1(1) **Sequences:** 1(1)
mitochondrial ATP synthase, H+ transporting F1 complex beta subunit [Mus musculus]

---

17. gi|192262 **Mass:** 54219 **Score:** 58 **Matches:** 1(1) **Sequences:** 1(1) **emPAI:** 0.06
pro-alpha-1 type I collagen, partial [Mus musculus]

Query	Observed	Mr(expt)	Mr(calc)	Delta	Miss	Score	Expect	Rank	Unique	Peptide
15700	589.5136	1177.0126	1176.5598	0.4528	0	58	0.0014	1	U	R.GQAGVMGFPGPK.G

Proteins matching the same set of peptides:
gi|470674 **Mass:** 137859 **Score:** 58 **Matches:** 1(1) **Sequences:** 1(1)
collagen pro-alpha-1 type I chain [Mus musculus]
gi|34328108 **Mass:** 137948 **Score:** 58 **Matches:** 1(1) **Sequences:** 1(1)
collagen alpha-1(I) chain precursor [Mus musculus]
gi|37589303 **Mass:** 117788 **Score:** 58 **Matches:** 1(1) **Sequences:** 1(1)
Col1a1 protein [Mus musculus]

---

18. gi|551295 **Mass:** 57824 **Score:** 57 **Matches:** 1(1) **Sequences:** 1(1) **emPAI:** 0.06

pyruvate kinase M [Mus musculus]

Query	Observed	Mr(expt)	Mr(calc)	Delta	Miss	Score	Expect	Rank	Unique	Peptide
18372	732.1815	1462.3485	1461.8079	0.5406	0	57	0.0032	1	U	K.IYVDDGLISLQVK.E

Proteins matching the same set of peptides:

gi|1405933 **Mass:** 57878 **Score:** 57 **Matches:** 1(1) **Sequences:** 1(1)
M2-type pyruvate kinase [Mus musculus]
gi|31981562 **Mass:** 57808 **Score:** 57 **Matches:** 1(1) **Sequences:** 1(1)
pyruvate kinase isozymes M1/M2 isoform 1 [Mus musculus]
gi|74151988 **Mass:** 57820 **Score:** 57 **Matches:** 1(1) **Sequences:** 1(1)
unnamed protein product [Mus musculus]
gi|74196318 **Mass:** 57807 **Score:** 57 **Matches:** 1(1) **Sequences:** 1(1)
unnamed protein product [Mus musculus]
gi|74212815 **Mass:** 43138 **Score:** 57 **Matches:** 1(1) **Sequences:** 1(1)
unnamed protein product [Mus musculus]
gi|74221210 **Mass:** 57809 **Score:** 57 **Matches:** 1(1) **Sequences:** 1(1)
unnamed protein product [Mus musculus]
gi|359807367 **Mass:** 57948 **Score:** 57 **Matches:** 1(1) **Sequences:** 1(1)
pyruvate kinase isozymes M1/M2 isoform 2 [Mus musculus]

---

19. gi|52789 **Mass:** 54415 **Score:** 57 **Matches:** 1(1) **Sequences:** 1(1) **emPAI:** 0.06
unnamed protein product [Mus musculus]

Query	Observed	Mr(expt)	Mr(calc)	Delta	Miss	Score	Expect	Rank	Unique	Peptide
17798	692.5499	1383.0852	1382.7194	0.3659	1	57	0.0016	1	U	K.SLNNKFASFIDK.V

Proteins matching the same set of peptides:

gi|309215 **Mass:** 53210 **Score:** 57 **Matches:** 1(1) **Sequences:** 1(1)
EndoA' cytokeratin (5' end put.); putative [Mus musculus]
gi|511654 **Mass:** 54220 **Score:** 57 **Matches:** 1(1) **Sequences:** 1(1)
keratin type II [Mus musculus]
gi|74177777 **Mass:** 54546 **Score:** 57 **Matches:** 1(1) **Sequences:** 1(1)
unnamed protein product [Mus musculus]
gi|74219975 **Mass:** 54459 **Score:** 57 **Matches:** 1(1) **Sequences:** 1(1)
unnamed protein product [Mus musculus]
gi|76779293 **Mass:** 54514 **Score:** 57 **Matches:** 1(1) **Sequences:** 1(1)
Keratin 8 [Mus musculus]
gi|114145561 **Mass:** 54531 **Score:** 57 **Matches:** 1(1) **Sequences:** 1(1)
keratin, type II cytoskeletal 8 [Mus musculus]

---

20. gi|46485130 **Mass:** 85186 **Score:** 57 **Matches:** 1(1) **Sequences:** 1(1) **emPAI:** 0.04
TPA_exp: keratin Kb40 [Mus musculus]

Query	Observed	Mr(expt)	Mr(calc)	Delta	Miss	Score	Expect	Rank	Unique	Peptide
17798	692.5499	1383.0852	1382.6830	0.4022	0	57	0.0017	2	U	R.SLNNQFASFIDK.V

Proteins matching the same set of peptides:

gi|111185567 **Score:** 57 **Matches:** 1(1) **Sequences:** 1(1)
gi|111185722 **Score:** 57 **Matches:** 1(1) **Sequences:** 1(1)
gi|119850791 **Score:** 57 **Matches:** 1(1) **Sequences:** 1(1)
gi|145580629 **Mass:** 112194 **Score:** 57 **Matches:** 1(1) **Sequences:** 1(1)
keratin Kb40 [Mus musculus]

---

21. gi|1304157 **Mass:** 72412 **Score:** 57 **Matches:** 1(1) **Sequences:** 1(1) **emPAI:** 0.05
78 kDa glucose-regulated protein [Mus musculus]

Query	Observed	Mr(expt)	Mr(calc)	Delta	Miss	Score	Expect	Rank	Unique	Peptide
18139	715.6650	1429.3155	1429.6838	-0.3682	0	57	0.0043	1	U	R.TWNDPSVQQDIK.F

Proteins matching the same set of peptides:
gi|2598562 **Mass:** 72433 **Score:** 57 **Matches:** 1(1) **Sequences:** 1(1)
BiP [Mus musculus]
gi|12835845 **Mass:** 72378 **Score:** 57 **Matches:** 1(1) **Sequences:** 1(1)
unnamed protein product [Mus musculus]
gi|74143673 **Mass:** 56858 **Score:** 57 **Matches:** 1(1) **Sequences:** 1(1)
unnamed protein product [Mus musculus]
gi|74188814 **Mass:** 72305 **Score:** 57 **Matches:** 1(1) **Sequences:** 1(1)
unnamed protein product [Mus musculus]
gi|74198293 **Mass:** 72419 **Score:** 57 **Matches:** 1(1) **Sequences:** 1(1)
unnamed protein product [Mus musculus]
gi|74198974 **Mass:** 72361 **Score:** 57 **Matches:** 1(1) **Sequences:** 1(1)
unnamed protein product [Mus musculus]
gi|74207492 **Mass:** 72301 **Score:** 57 **Matches:** 1(1) **Sequences:** 1(1)
unnamed protein product [Mus musculus]
gi|74220199 **Mass:** 68455 **Score:** 57 **Matches:** 1(1) **Sequences:** 1(1)
unnamed protein product [Mus musculus]
gi|74225394 **Mass:** 72337 **Score:** 57 **Matches:** 1(1) **Sequences:** 1(1)
unnamed protein product [Mus musculus]
gi|254540166 **Mass:** 72377 **Score:** 57 **Matches:** 1(1) **Sequences:** 1(1)
78 kDa glucose-regulated protein precursor [Mus musculus]

---

22. gi|4159806 **Mass:** 65183 **Score:** 55 **Matches:** 2(2) **Sequences:** 2(2) **emPAI:** 0.10
type II keratin subunit protein [Mus musculus]

Query	Observed	Mr(expt)	Mr(calc)	Delta	Miss	Score	Expect	Rank	Unique	Peptide
17798	692.5499	1383.0852	1383.7034	-0.6182	1	44	0.031	3	U	K.SLNDKFASFIDK.V
17884	697.6450	1393.2755	1392.7249	0.5506	1	55	0.0067	1	U	R.TNAENEFVTIKK.D

Proteins matching the same set of peptides:
gi|12859782 **Mass:** 65586 **Score:** 55 **Matches:** 2(2) **Sequences:** 2(2)
unnamed protein product [Mus musculus]
gi|126116585 **Mass:** 65565 **Score:** 55 **Matches:** 2(2) **Sequences:** 2(2)
keratin, type II cytoskeletal 1 [Mus musculus]

---

23. gi|293686 **Mass:** 59414 **Score:** 55 **Matches:** 1(1) **Sequences:** 1(1) **emPAI:** 0.06
epidermal keratin subunit II [Mus musculus]

Query	Observed	Mr(expt)	Mr(calc)	Delta	Miss	Score	Expect	Rank	Unique	Peptide
17884	697.6450	1393.2755	1393.7089	-0.4334	1	55	0.0067	1	U	R.TDAENEFVTLKK.D

---

24. gi|3687320 **Mass:** 46137 **Score:** 52 **Matches:** 1(1) **Sequences:** 1(1) **emPAI:** 0.07
cartilage-associated protein (CASP) [Mus musculus]

Query	Observed	Mr(expt)	Mr(calc)	Delta	Miss	Score	Expect	Rank	Unique	Peptide
19209	840.6470	1679.2794	1678.8488	0.4306	0	52	0.01	1	U	R.TSISDMELALPDFLK.A

Proteins matching the same set of peptides:

gi|12857227 **Mass:** 46121 **Score:** 52 **Matches:** 1(1) **Sequences:** 1(1)
unnamed protein product [Mus musculus]
gi|148677365 **Mass:** 46110 **Score:** 52 **Matches:** 1(1) **Sequences:** 1(1)
mCG7119 [Mus musculus]
gi|225543173 **Mass:** 46140 **Score:** 52 **Matches:** 1(1) **Sequences:** 1(1)
cartilage-associated protein precursor [Mus musculus]

---

25. gi|74203983 **Mass:** 60183 **Score:** 51 **Matches:** 1(1) **Sequences:** 1(1) **emPAI:** 0.05
unnamed protein product [Mus musculus]

Query	Observed	Mr(expt)	Mr(calc)	Delta	Miss	Score	Expect	Rank	Unique	Peptide
17926	700.4083	1398.8021	1398.6159	0.1862	0	51	0.02	1	U	K.SIDMSWDSVTMK.H

Proteins matching the same set of peptides:
gi|76677895 **Mass:** 58508 **Score:** 51 **Matches:** 1(1) **Sequences:** 1(1)
poly(U)-binding-splicing factor PUF60 isoform b [Mus musculus]
gi|111185612 **Mass:** 26264 **Score:** 51 **Matches:** 1(1) **Sequences:** 1(1)
Puf60 protein [Mus musculus]
gi|148697575 **Mass:** 58380 **Score:** 51 **Matches:** 1(1) **Sequences:** 1(1)
RIKEN cDNA 2410104I19, isoform CRA_c [Mus musculus]
gi|148697576 **Mass:** 58982 **Score:** 51 **Matches:** 1(1) **Sequences:** 1(1)
RIKEN cDNA 2410104I19, isoform CRA_d [Mus musculus]
gi|257196183 **Mass:** 60211 **Score:** 51 **Matches:** 1(1) **Sequences:** 1(1)
poly(U)-binding-splicing factor PUF60 isoform a [Mus musculus]
gi|257196186 **Mass:** 53995 **Score:** 51 **Matches:** 1(1) **Sequences:** 1(1)
poly(U)-binding-splicing factor PUF60 isoform c [Mus musculus]

---

26. gi|468546 **Mass:** 57411 **Score:** 50 **Matches:** 1(1) **Sequences:** 1(1) **emPAI:** 0.06
CCT (chaperonin containing TCP-1) beta subunit [Mus musculus]

Query	Observed	Mr(expt)	Mr(calc)	Delta	Miss	Score	Expect	Rank	Unique	Peptide
18778	767.4490	1532.8834	1531.7916	1.0918	0	50	0.023	1	U	R.DAALMVTNDGATILK.N

Proteins matching the same set of peptides:
gi|7670405 **Mass:** 52436 **Score:** 50 **Matches:** 1(1) **Sequences:** 1(1)
unnamed protein product [Mus musculus]
gi|126521835 **Mass:** 57441 **Score:** 50 **Matches:** 1(1) **Sequences:** 1(1)
T-complex protein 1 subunit beta [Mus musculus]
gi|148689878 **Mass:** 44497 **Score:** 50 **Matches:** 1(1) **Sequences:** 1(1)
chaperonin subunit 2 (beta), isoform CRA_b [Mus musculus]

---

27. gi|22297268 **Score:** 50 **Matches:** 1(1) **Sequences:** 1(1) **emPAI:** 0.16
AID, partial [Homo sapiens]

Query	Observed	Mr(expt)	Mr(calc)	Delta	Miss	Score	Expect	Rank	Unique	Peptide
10277	381.1882	760.3618	760.3715	-0.0097	0	50	0.015	2	U	K.AESEGLR.R

---

28. gi|51450 **Mass:** 46560 **Score:** 49 **Matches:** 1(1) **Sequences:** 1(1) **emPAI:** 0.07
heat shock protein [Mus musculus]

Query	Observed	Mr(expt)	Mr(calc)	Delta	Miss	Score	Expect	Rank	Unique	Peptide
16265	612.9139	1223.8133	1223.6510	0.1623	0	49	0.027	1	U	K.GVVEVTHDLQK.H

Proteins matching the same set of peptides:

gi|200966 **Mass:** 45623 **Score:** 49 **Matches:** 1(1) **Sequences:** 1(1)
put. serine protease inhibitor [Mus musculus]
gi|26345418 **Mass:** 46481 **Score:** 49 **Matches:** 1(1) **Sequences:** 1(1)
unnamed protein product [Mus musculus]
gi|26348007 **Mass:** 46490 **Score:** 49 **Matches:** 1(1) **Sequences:** 1(1)
unnamed protein product [Mus musculus]
gi|74139266 **Mass:** 46490 **Score:** 49 **Matches:** 1(1) **Sequences:** 1(1)
unnamed protein product [Mus musculus]
gi|74191337 **Mass:** 46505 **Score:** 49 **Matches:** 1(1) **Sequences:** 1(1)
unnamed protein product [Mus musculus]
gi|74198254 **Mass:** 46500 **Score:** 49 **Matches:** 1(1) **Sequences:** 1(1)
unnamed protein product [Mus musculus]
gi|148684430 **Mass:** 44954 **Score:** 49 **Matches:** 1(1) **Sequences:** 1(1)
serine (or cysteine) peptidase inhibitor, clade H, member 1, isoform CRA_a [Mus musculus]
gi|161353502 **Mass:** 46504 **Score:** 49 **Matches:** 1(1) **Sequences:** 1(1)
serpin H1 precursor [Mus musculus]

---

29. gi|6680748 **Mass:** 59716 **Score:** 49 **Matches:** 1(1) **Sequences:** 1(1) **emPAI:** 0.06
ATP synthase subunit alpha, mitochondrial precursor [Mus musculus]

Query	Observed	Mr(expt)	Mr(calc)	Delta	Miss	Score	Expect	Rank	Unique	Peptide
9365	362.2126	722.4106	722.4439	-0.0333	0	49	0.012	1	U	K.APGIIPR.I

Proteins matching the same set of peptides:
gi|74139457 **Mass:** 59656 **Score:** 49 **Matches:** 1(1) **Sequences:** 1(1)
unnamed protein product [Mus musculus]
gi|74146998 **Mass:** 55919 **Score:** 49 **Matches:** 1(1) **Sequences:** 1(1)
unnamed protein product [Mus musculus]
gi|74182195 **Mass:** 32607 **Score:** 49 **Matches:** 1(1) **Sequences:** 1(1)
unnamed protein product [Mus musculus]
gi|74211072 **Mass:** 59730 **Score:** 49 **Matches:** 1(1) **Sequences:** 1(1)
unnamed protein product [Mus musculus]
gi|74211198 **Mass:** 51304 **Score:** 49 **Matches:** 1(1) **Sequences:** 1(1)
unnamed protein product [Mus musculus]
gi|74211977 **Mass:** 59746 **Score:** 49 **Matches:** 1(1) **Sequences:** 1(1)
unnamed protein product [Mus musculus]
gi|148677500 **Mass:** 51819 **Score:** 49 **Matches:** 1(1) **Sequences:** 1(1)
ATP synthase, H+ transporting, mitochondrial F1 complex, alpha subunit, isoform 1, isoform CRA_d [Mus musculus]
gi|148677501 **Mass:** 54561 **Score:** 49 **Matches:** 1(1) **Sequences:** 1(1)
ATP synthase, H+ transporting, mitochondrial F1 complex, alpha subunit, isoform 1, isoform CRA_e [Mus musculus]
gi|148677504 **Mass:** 54738 **Score:** 49 **Matches:** 1(1) **Sequences:** 1(1)
ATP synthase, H+ transporting, mitochondrial F1 complex, alpha subunit, isoform 1, isoform CRA_h [Mus musculus]

---

30. gi|148688671 **Score:** 49 **Matches:** 1(1) **Sequences:** 1(1) **emPAI:** 0.30
mCG16097 [Mus musculus]

Query	Observed	Mr(expt)	Mr(calc)	Delta	Miss	Score	Expect	Rank	Unique	Peptide
9365	362.2126	722.4106	722.4439	-0.0333	0	49	0.012	1	U	K.APGLLPR.L

---

31. gi|6755893 **Mass:** 26257 **Score:** 48 **Matches:** 2(2) **Sequences:** 1(1) **emPAI:** 0.27
trypsin 4 precursor [Mus musculus]

Query	Observed	Mr(expt)	Mr(calc)	Delta	Miss	Score	Expect	Rank	Unique	Peptide
18452	737.6481	2209.9224	2211.0920	-1.1696	0	47	0.039	2	U	R.LGEHNINVLEGNEQFVNSAK.I

19321	1106.1886	2210.3626	2211.0920	-0.7293	0	(47)	0.044	2	U	R.LGEHNINVLEGNEQFVNSAK.I

Proteins matching the same set of peptides:
gi|51010909 **Mass:** 26260 **Score:** 48 **Matches:** 2(2) **Sequences:** 1(1)
trypsin 5 precursor [Mus musculus]
gi|74203392 **Mass:** 27146 **Score:** 48 **Matches:** 2(2) **Sequences:** 1(1)
unnamed protein product [Mus musculus]

---

32. gi|148707748 **Score:** 47 **Matches:** 1(1) **Sequences:** 1(1) **emPAI:** 0.10
nuclear casein kinase and cyclin-dependent kinase substrate 1, isoform CRA_b [Mus musculus]

Query	Observed	Mr(expt)	Mr(calc)	Delta	Miss	Score	Expect	Rank	Unique	Peptide
18778	767.4490	1532.8834	1531.6525	1.2309	1	47	0.044	2	U	K.EEDEEAESPPEKK.S

Proteins matching the same set of peptides:
gi|224809559 **Score:** 47 **Matches:** 1(1) **Sequences:** 1(1)
gi|224809563 **Score:** 47 **Matches:** 1(1) **Sequences:** 1(1)

---

33. gi|53830774 **Mass:** 34092 **Score:** 47 **Matches:** 1(1) **Sequences:** 1(1) **emPAI:** 0.10
lactase [Mus musculus]

Query	Observed	Mr(expt)	Mr(calc)	Delta	Miss	Score	Expect	Rank	Unique	Peptide
14788	**548.6042**	**1095.1938**	**1094.5132**	**0.6806**	**0**	**47**	**0.016**	**1**	**U**	**R.LPEFTESEK.K**

Proteins matching the same set of peptides:
gi|74192292 **Mass:** 139170 **Score:** 47 **Matches:** 1(1) **Sequences:** 1(1)
unnamed protein product [Mus musculus]
gi|124487297 **Mass:** 217665 **Score:** 47 **Matches:** 1(1) **Sequences:** 1(1)
lactase-phlorizin hydrolase preproprotein [Mus musculus]
gi|148707805 **Mass:** 217693 **Score:** 47 **Matches:** 1(1) **Sequences:** 1(1)
mCG128560 [Mus musculus]

---

34. gi|26324736 **Score:** 47 **Matches:** 1(1) **Sequences:** 1(1) **emPAI:** 0.06
unnamed protein product [Mus musculus]

Query	Observed	Mr(expt)	Mr(calc)	Delta	Miss	Score	Expect	Rank	Unique	Peptide
17059	651.8716	1301.7286	1300.7238	1.0048	0	47	0.029	2	U	R.SLNLDSIIAEVK.A

Proteins matching the same set of peptides:
gi|109735018 **Score:** 47 **Matches:** 1(1) **Sequences:** 1(1)
gi|148672090 **Score:** 47 **Matches:** 1(1) **Sequences:** 1(1)
gi|269914154 **Score:** 47 **Matches:** 1(1) **Sequences:** 1(1)

---

35. gi|7513694 **Mass:** 109137 **Score:** 46 **Matches:** 1(1) **Sequences:** 1(1) **emPAI:** 0.03
IgG Fc binding protein - mouse (fragment)

Query	Observed	Mr(expt)	Mr(calc)	Delta	Miss	Score	Expect	Rank	Unique	Peptide
12529	**437.9564**	**873.8983**	**873.4920**	**0.4063**	**0**	**46**	**0.022**	**1**	**U**	**R.ISVINGGSK.A**

Proteins matching the same set of peptides:
gi|148692210 **Mass:** 147962 **Score:** 46 **Matches:** 1(1) **Sequences:** 1(1)
mCG145390, isoform CRA_a [Mus musculus]
gi|148692211 **Mass:** 146729 **Score:** 46 **Matches:** 1(1) **Sequences:** 1(1)
mCG145390, isoform CRA_b [Mus musculus]

gi|169790797 **Mass:** 275058 **Score:** 46 **Matches:** 1(1) **Sequences:** 1(1)
Fc fragment of IgG binding protein precursor [Mus musculus]
gi|257467625 **Mass:** 280044 **Score:** 46 **Matches:** 1(1) **Sequences:** 1(1)
Fc fragment of IgG binding protein-like precursor [Mus musculus]

---

36. gi|111599858 **Score:** 46 **Matches:** 1(1) **Sequences:** 1(1) **emPAI:** 0.03
Dehydrogenase E1 and transketolase domain containing 1 [Mus musculus]

Query	Observed	Mr(expt)	Mr(calc)	Delta	Miss	Score	Expect	Rank	Unique	Peptide
10277	381.1882	760.3618	759.3875	0.9743	1	46	0.034	3	U	K.AKSGEPR.G

Proteins matching the same set of peptides:
gi|124487485 **Score:** 46 **Matches:** 1(1) **Sequences:** 1(1)

---

37. gi|74224513 **Mass:** 37387 **Score:** 44 **Matches:** 1(1) **Sequences:** 1(1) **emPAI:** 0.09
unnamed protein product [Mus musculus]

Query	Observed	Mr(expt)	Mr(calc)	Delta	Miss	Score	Expect	Rank	Unique	Peptide
11939	**419.3358**	**836.6570**	**835.5280**	**1.1290**	**0**	**44**	**0.035**	**1**	**U**	**R.VAAPPLLR.V**

---

38. gi|21410133 **Score:** 44 **Matches:** 1(1) **Sequences:** 1(1) **emPAI:** 0.13
Kidins220 protein [Mus musculus]

Query	Observed	Mr(expt)	Mr(calc)	Delta	Miss	Score	Expect	Rank	Unique	Peptide
10277	381.1882	760.3618	759.3875	0.9743	1	44	0.055	4	U	K.AEGKAER.V

Proteins matching the same set of peptides:
gi|28385979 **Mass:** 172572 **Score:** 44 **Matches:** 1(1) **Sequences:** 1(1)
Kidins220 protein [Mus musculus]
gi|28972688 **Mass:** 188137 **Score:** 44 **Matches:** 1(1) **Sequences:** 1(1)
mKIAA1250 protein [Mus musculus]
gi|62826025 **Score:** 44 **Matches:** 1(1) **Sequences:** 1(1)
gi|124487039 **Mass:** 199059 **Score:** 44 **Matches:** 1(1) **Sequences:** 1(1)
kinase D-interacting substrate of 220 kDa [Mus musculus]
gi|148705033 **Mass:** 190650 **Score:** 44 **Matches:** 1(1) **Sequences:** 1(1)
mCG18896 [Mus musculus]
gi|187957160 **Mass:** 185440 **Score:** 44 **Matches:** 1(1) **Sequences:** 1(1)
Kidins220 protein [Mus musculus]

---

39. gi|26333649 **Score:** 44 **Matches:** 1(1) **Sequences:** 1(1) **emPAI:** 0.07
unnamed protein product [Mus musculus]

Query	Observed	Mr(expt)	Mr(calc)	Delta	Miss	Score	Expect	Rank	Unique	Peptide
13828	496.0008	989.9870	989.5182	0.4689	1	44	0.04	2	U	K.FPKDAGLDK.L

Proteins matching the same set of peptides:
gi|74146722 **Score:** 44 **Matches:** 1(1) **Sequences:** 1(1)
gi|74192493 **Score:** 44 **Matches:** 1(1) **Sequences:** 1(1)
gi|113865995 **Score:** 44 **Matches:** 1(1) **Sequences:** 1(1)
gi|116325981 **Score:** 44 **Matches:** 1(1) **Sequences:** 1(1)
gi|148669182 **Score:** 44 **Matches:** 1(1) **Sequences:** 1(1)
gi|148707033 **Score:** 44 **Matches:** 1(1) **Sequences:** 1(1)
gi|254847433 **Score:** 44 **Matches:** 1(1) **Sequences:** 1(1)

gi|254847435 **Score:** 44 **Matches:** 1(1) **Sequences:** 1(1)
gi|254847438 **Score:** 44 **Matches:** 1(1) **Sequences:** 1(1)
gi|377837285 **Score:** 44 **Matches:** 1(1) **Sequences:** 1(1)
gi|377837287 **Score:** 44 **Matches:** 1(1) **Sequences:** 1(1)

---

40. gi|986918 **Mass:** 51416 **Score:** 44 **Matches:** 1(1) **Sequences:** 1(1) **emPAI:** 0.06
A10 [Mus musculus]

Query	Observed	Mr(expt)	Mr(calc)	Delta	Miss	Score	Expect	Rank	Unique	Peptide
15221	566.0025	1129.9904	1129.5979	0.3925	0	44	0.036	1	U	K.VTADVINAAEK.L

Proteins matching the same set of peptides:
gi|26345686 **Mass:** 56546 **Score:** 44 **Matches:** 1(1) **Sequences:** 1(1)
unnamed protein product [Mus musculus]
gi|52353955 **Mass:** 56549 **Score:** 44 **Matches:** 1(1) **Sequences:** 1(1)
D-3-phosphoglycerate dehydrogenase [Mus musculus]

---

41. gi|21313084 **Mass:** 56107 **Score:** 44 **Matches:** 1(1) **Sequences:** 1(1) **emPAI:** 0.06
chromodomain Y-like protein 2 [Mus musculus]

Query	Observed	Mr(expt)	Mr(calc)	Delta	Miss	Score	Expect	Rank	Unique	Peptide
15639	585.4729	1168.9312	1168.6676	0.2636	1	44	0.035	1	U	K.RVNSPLSRPK.K

Proteins matching the same set of peptides:
gi|32479356 **Mass:** 56107 **Score:** 44 **Matches:** 1(1) **Sequences:** 1(1)
chromodomain Y-like protein 2 [Mus musculus]
gi|74206467 **Mass:** 56077 **Score:** 44 **Matches:** 1(1) **Sequences:** 1(1)
unnamed protein product [Mus musculus]

---

42. gi|124504325 **Mass:** 128405 **Score:** 44 **Matches:** 1(0) **Sequences:** 1(0) **emPAI:** 0.03
Armcx4 protein [Mus musculus]

Query	Observed	Mr(expt)	Mr(calc)	Delta	Miss	Score	Expect	Rank	Unique	Peptide
12706	887.4671	886.4599	886.4621	-0.0022	1	44	0.071	1	U	K.GNRNSVPK.A

Proteins matching the same set of peptides:
gi|363989837 **Mass:** 242806 **Score:** 44 **Matches:** 1(0) **Sequences:** 1(0)
armadillo repeat-containing X-linked protein 4 [Mus musculus]

---

43. gi|12843046 **Mass:** 26390 **Score:** 43 **Matches:** 1(0) **Sequences:** 1(0) **emPAI:** 0.13
unnamed protein product [Mus musculus]

Query	Observed	Mr(expt)	Mr(calc)	Delta	Miss	Score	Expect	Rank	Unique	Peptide
18063	711.1305	1420.2464	1419.7146	0.5318	0	43	0.081	1	U	K.YVNWIQQTIAAN.-

Proteins matching the same set of peptides:
gi|71043961 **Mass:** 26405 **Score:** 43 **Matches:** 1(0) **Sequences:** 1(0)
trypsinogen 7 precursor [Mus musculus]
gi|148681611 **Mass:** 25182 **Score:** 43 **Matches:** 1(0) **Sequences:** 1(0)
RIKEN cDNA 2210010C04, isoform CRA_a [Mus musculus]

---

44. gi|12836661 **Mass:** 117980 **Score:** 43 **Matches:** 1(1) **Sequences:** 1(1) **emPAI:** 0.03
unnamed protein product [Mus musculus]

Query	Observed	Mr(expt)	Mr(calc)	Delta	Miss	Score	Expect	Rank	Unique	Peptide

14417	530.9701	1059.9256	1059.5093	0.4164	0	43	0.045	1	U	R.CPPPLACALK.A

Proteins matching the same set of peptides:
gi\|16741201 **Mass:** 70327 **Score:** 43 **Matches:** 1(1) **Sequences:** 1(1)
Pelp1 protein [Mus musculus]
gi\|37046775 **Mass:** 91728 **Score:** 43 **Matches:** 1(1) **Sequences:** 1(1)
Pelp1 protein, partial [Mus musculus]
gi\|257900472 **Mass:** 117995 **Score:** 43 **Matches:** 1(1) **Sequences:** 1(1)
proline-, glutamic acid- and leucine-rich protein 1 [Mus musculus]

---

45. gi\|148681245 **Mass:** 9487 **Score:** 43 **Matches:** 1(0) **Sequences:** 1(0) **emPAI:** 0.37
mCG145199 [Mus musculus]

Query	Observed	Mr(expt)	Mr(calc)	Delta	Miss	Score	Expect	Rank	Unique	Peptide
11940	419.3534	836.6922	836.4729	0.2193	1	43	0.085	1	U	R.LAGHGRAR.S

---

46. gi\|148667455 **Mass:** 58456 **Score:** 43 **Matches:** 1(1) **Sequences:** 1(1) **emPAI:** 0.06
TEA domain family member 4, isoform CRA_b [Mus musculus]

Query	Observed	Mr(expt)	Mr(calc)	Delta	Miss	Score	Expect	Rank	Unique	Peptide
14488	534.0168	1066.0190	1066.5995	-0.5805	1	43	0.041	1	U	R.ARGPAAVAAAGR.L

Proteins matching the same set of peptides:
gi\|148667459 **Mass:** 62771 **Score:** 43 **Matches:** 1(1) **Sequences:** 1(1)
TEA domain family member 4, isoform CRA_f [Mus musculus]
gi\|148667454 **Mass:** 59545 **Score:** 43 **Matches:** 1(1) **Sequences:** 1(1)
TEA domain family member 4, isoform CRA_a [Mus musculus]

---

47. gi\|642072 **Mass:** 118421 **Score:** 43 **Matches:** 1(0) **Sequences:** 1(0) **emPAI:** 0.03
fibrillin-1, partial [Mus musculus]

Query	Observed	Mr(expt)	Mr(calc)	Delta	Miss	Score	Expect	Rank	Unique	Peptide
14550	536.8515	1071.6884	1070.4451	1.2433	0	43	0.076	1	U	R.CVNTDGSYR.C

Proteins matching the same set of peptides:
gi\|726324 **Mass:** 312194 **Score:** 43 **Matches:** 1(0) **Sequences:** 1(0)
fibrillin-1 [Mus musculus]
gi\|2494284 **Mass:** 312051 **Score:** 43 **Matches:** 1(0) **Sequences:** 1(0)
RecName: Full=Fibrillin-1; Flags: Precursor
gi\|3688648 **Mass:** 418019 **Score:** 43 **Matches:** 1(0) **Sequences:** 1(0)
mutant fibrillin-1 [Mus musculus]
gi\|118197277 **Mass:** 312083 **Score:** 43 **Matches:** 1(0) **Sequences:** 1(0)
fibrillin-1 precursor [Mus musculus]
gi\|148696192 **Mass:** 312056 **Score:** 43 **Matches:** 1(0) **Sequences:** 1(0)
fibrillin 1, isoform CRA_a [Mus musculus]

---

48. gi\|12836791 **Mass:** 23191 **Score:** 43 **Matches:** 1(0) **Sequences:** 1(0) **emPAI:** 0.14
unnamed protein product [Mus musculus]

Query	Observed	Mr(expt)	Mr(calc)	Delta	Miss	Score	Expect	Rank	Unique	Peptide
16636	629.0249	1256.0352	1255.5478	0.4874	0	43	0.11	1	U	-.MPPGPCAWPPR.A

Proteins matching the same set of peptides:

gi|47059145 **Mass:** 23306 **Score:** 43 **Matches:** 1(0) **Sequences:** 1(0)
fibronectin type III domain-containing protein 5 precursor [Mus musculus]

---

49. gi|14250408 **Mass:** 57081 **Score:** 42 **Matches:** 1(0) **Sequences:** 1(0) **emPAI:** 0.06
Aspartyl-tRNA synthetase [Mus musculus]

Query	Observed	Mr(expt)	Mr(calc)	Delta	Miss	Score	Expect	Rank	Unique	Peptide
15565	581.3489	1160.6833	1159.6601	1.0232	0	42	0.077	1	U	K.IYVISLAEPR.L

Proteins matching the same set of peptides:
gi|26346400 **Mass:** 57111 **Score:** 42 **Matches:** 1(0) **Sequences:** 1(0)
unnamed protein product [Mus musculus]
gi|74139282 **Mass:** 57145 **Score:** 42 **Matches:** 1(0) **Sequences:** 1(0)
unnamed protein product [Mus musculus]
gi|74141887 **Mass:** 57067 **Score:** 42 **Matches:** 1(0) **Sequences:** 1(0)
unnamed protein product [Mus musculus]
gi|74226918 **Mass:** 57109 **Score:** 42 **Matches:** 1(0) **Sequences:** 1(0)
unnamed protein product [Mus musculus]
gi|148707802 **Mass:** 64317 **Score:** 42 **Matches:** 1(0) **Sequences:** 1(0)
aspartyl-tRNA synthetase [Mus musculus]
gi|211065507 **Mass:** 57111 **Score:** 42 **Matches:** 1(0) **Sequences:** 1(0)
aspartate--tRNA ligase, cytoplasmic isoform 1 [Mus musculus]

---

50. gi|17512475 **Mass:** 46233 **Score:** 42 **Matches:** 1(0) **Sequences:** 1(0) **emPAI:** 0.07
Ankyrin repeat and SOCS box-containing 6 [Mus musculus]

Query	Observed	Mr(expt)	Mr(calc)	Delta	Miss	Score	Expect	Rank	Unique	Peptide
18446	736.9879	2207.9419	2208.1902	-0.2483	1	42	0.14	1	U	K.IQALHASLRQLESYPPPLK.H

Proteins matching the same set of peptides:
gi|19111154 **Mass:** 46247 **Score:** 42 **Matches:** 1(0) **Sequences:** 1(0)
ankyrin repeat and SOCS box protein 6 [Mus musculus]
gi|26354010 **Mass:** 46217 **Score:** 42 **Matches:** 1(0) **Sequences:** 1(0)
unnamed protein product [Mus musculus]

---

51. gi|309271872 **Mass:** 403142 **Score:** 42 **Matches:** 1(1) **Sequences:** 1(1) **emPAI:** 0.01
PREDICTED: zinc finger protein 469 [Mus musculus]

Query	Observed	Mr(expt)	Mr(calc)	Delta	Miss	Score	Expect	Rank	Unique	Peptide
14830	551.5288	1101.0431	1101.5051	-0.4620	0	42	0.054	1	U	R.SSPGQPSSSPR.L

---

52. gi|26380166 **Mass:** 13950 **Score:** 42 **Matches:** 1(1) **Sequences:** 1(1) **emPAI:** 0.24
unnamed protein product [Mus musculus]

Query	Observed	Mr(expt)	Mr(calc)	Delta	Miss	Score	Expect	Rank	Unique	Peptide
17965	703.0179	1404.0212	1404.6885	-0.6673	0	42	0.056	1	U	K.GPSEGAYDVILPR.A

Proteins matching the same set of peptides:
gi|46395891 **Mass:** 48390 **Score:** 42 **Matches:** 1(1) **Sequences:** 1(1)
RecName: Full=G-protein coupled receptor family C group 5 member C; AltName: Full=Retinoic acid-induced gene 3 protein; Short=RAIG-3; Flags: Precursor
gi|148669181 **Mass:** 48467 **Score:** 42 **Matches:** 1(1) **Sequences:** 1(1)
G protein-coupled receptor, family C, group 5, member C [Mus musculus]
gi|160333231 **Mass:** 48453 **Score:** 42 **Matches:** 1(1) **Sequences:** 1(1)
G-protein coupled receptor family C group 5 member C isoform a precursor [Mus musculus]

gi|160333233 **Mass:** 48416 **Score:** 42 **Matches:** 1(1) **Sequences:** 1(1)
G-protein coupled receptor family C group 5 member C isoform b precursor [Mus musculus]
gi|215406578 **Mass:** 11983 **Score:** 42 **Matches:** 1(1) **Sequences:** 1(1)
G protein-coupled receptor, family C, group 5, member C [Mus musculus]

Mascot: http://www.matrixscience.com/

PFHR9

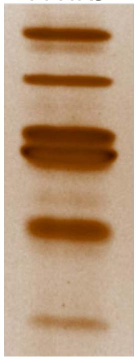

4

Mascot Search Results

```
User                   :
Email                  :
Search title           :
MS data file           : ZIEN-29Nov2017_4m.mgf
Database               : NCBInr 20120419 (17893860 sequences; 6141683785 residues)
Taxonomy               : Mus musculus (house mouse) (138831 sequences)
Timestamp              : 30 Nov 2017 at 20:51:28 GMT
Enzyme                 : Trypsin
Variable modifications : Oxidation (M),Carbamidomethyl (C),Oxidation (P)
Mass values            : Monoisotopic
Protein Mass           : Unrestricted
Peptide Mass Tolerance : ± 1.25 Da
Fragment Mass Tolerance: ± 1.001 Da
Max Missed Cleavages   : 1
Instrument type        : Default
Number of queries      : 16414
Protein hits           : gi|22164776  keratin, type II cytoskeletal 79 [Mus musculus]
                         gi|51450     heat shock protein [Mus musculus]
                         gi|7638398   epidermal keratin 10 [Mus musculus]
                         gi|16716569  protease, serine, 1 precursor [Mus musculus]
                         gi|12843914  unnamed protein product [Mus musculus]
                         gi|9910294   keratin, type II cytoskeletal 71 [Mus musculus]
                         gi|2498741   RecName: Full=Prolyl 4-hydroxylase subunit alpha-2; Short=4-PH alpha-2; AltName: Full=Procollagen-proline,2-oxoglutarate-4-dioxygenase subunit alpha-2; Flags: Precursc
                         gi|148672076 mCG17605, isoform CRA_b [Mus musculus]
                         gi|51092303  Try10-like trypsinogen precursor [Mus musculus]
                         gi|4159806   type II keratin subunit protein [Mus musculus]
                         gi|6166378   growth suppressor 1L [Mus musculus]
                         gi|295841496 tubulin beta 3 [Mus musculus]
                         gi|438134    DNA-directed DNA polymerase [Mus musculus]
                         gi|6680888   CD63 antigen [Mus musculus]
                         gi|202210    alpha-tubulin isotype M-alpha-2 [Mus musculus]
                         gi|3687320   cartilage-associated protein (CASP) [Mus musculus]
                         gi|85701680  keratin, type II cytoskeletal 2 oral [Mus musculus]
                         gi|21489935  keratin, type I cytoskeletal 14 [Mus musculus]
                         gi|468546    CCT (chaperonin containing TCP-1) beta subunit [Mus musculus]
                         gi|6755893   trypsin 4 precursor [Mus musculus]
                         gi|27370092  elongation factor Tu, mitochondrial isoform 1 [Mus musculus]
                         gi|293686    epidermal keratin subunit II [Mus musculus]
                         gi|3212116   prefoldin subunit 2 [Mus musculus]
                         gi|193645    glucose-regulated protein 78, partial [Mus musculus]
                         gi|53830774  lactase [Mus musculus]
                         gi|81872882  RecName: Full=Adenomatous polyposis coli protein 2
                         gi|191765    alpha-fetoprotein, partial [Mus musculus]
                         gi|54777     unnamed protein product [Mus musculus]
                         gi|26390362  unnamed protein product [Mus musculus]
```

Select Summary Report

Format As | Select Summary (protein hits) | Help

Significance threshold p< 0.05 | Max. number of hits AUTO

Standard scoring ○ MudPIT scoring ◉ | Ions score or expect cut-off 43 | Show sub-sets 0

Show pop-ups ◉ Suppress pop-ups ○ | Require bold red ☐

Re-Search ◉ All queries ○ Unassigned ○ Below homology threshold ○ Below identity threshold

1. gi|22164776 **Mass:** 57517 **Score:** 102 **Matches:** 2(2) **Sequences:** 1(1) **emPAI:** 0.06

keratin, type II cytoskeletal 79 [Mus musculus]

Query	Observed	Mr(expt)	Mr(calc)	Delta	Miss	Score	Expect	Rank	Unique	Peptide
14241	665.9768	1329.9391	1328.7187	1.2204	0	79	1.2e-005	1	U	R.NLDLDSIIAEVK.A 14234

Proteins matching the same set of peptides:

gi|148672069 **Mass:** 59231 **Score:** 102 **Matches:** 2(2) **Sequences:** 1(1)
cDNA sequence BC031593 [Mus musculus]

---

2. gi|51450 **Mass:** 46560 **Score:** 102 **Matches:** 4(3) **Sequences:** 4(3) **emPAI:** 0.32
heat shock protein [Mus musculus]

Query	Observed	Mr(expt)	Mr(calc)	Delta	Miss	Score	Expect	Rank	Unique	Peptide
14017	653.5609	1305.1073	1305.6677	-0.5604	0	67	0.00035	1	U	R.DNQSGSLLFIGR.L
14227	665.1298	1328.2451	1327.6628	0.5823	0	52	0.013	1	U	K.LQMVEMPLAHK.L
14757	690.9507	1379.8868	1379.5881	0.2987	0	44	0.097	1	U	R.TGLYNYYDDEK.E
16290	830.6638	1659.3131	1658.7941	0.5190	0	59	0.0027	1	U	R.LYGPSSVSFADDFVR.S

Proteins matching the same set of peptides:
gi|200966 **Mass:** 45623 **Score:** 102 **Matches:** 4(3) **Sequences:** 4(3)
put. serine protease inhibitor [Mus musculus]
gi|26345418 **Mass:** 46481 **Score:** 102 **Matches:** 4(3) **Sequences:** 4(3)
unnamed protein product [Mus musculus]
gi|26348007 **Mass:** 46490 **Score:** 102 **Matches:** 4(3) **Sequences:** 4(3)
unnamed protein product [Mus musculus]
gi|74191337 **Mass:** 46505 **Score:** 102 **Matches:** 4(3) **Sequences:** 4(3)
unnamed protein product [Mus musculus]
gi|74198254 **Mass:** 46500 **Score:** 102 **Matches:** 4(3) **Sequences:** 4(3)
unnamed protein product [Mus musculus]
gi|148684430 **Mass:** 44954 **Score:** 102 **Matches:** 4(3) **Sequences:** 4(3)
serine (or cysteine) peptidase inhibitor, clade H, member 1, isoform CRA_a [Mus musculus]
gi|161353502 **Mass:** 46504 **Score:** 102 **Matches:** 4(3) **Sequences:** 4(3)
serpin H1 precursor [Mus musculus]

---

3. gi|7638398 **Mass:** 34962 **Score:** 85 **Matches:** 2(2) **Sequences:** 2(2) **emPAI:** 0.20
epidermal keratin 10 [Mus musculus]

Query	Observed	Mr(expt)	Mr(calc)	Delta	Miss	Score	Expect	Rank	Unique	Peptide
11545	546.0249	1090.0351	1089.5237	0.5115	0	46	0.023	1		K.VTMQNLNDR.L
14774	691.5409	1381.0672	1380.6408	0.4264	0	80	1.6e-005	1	U	R.ALEESNYELEGK.I

Proteins matching the same set of peptides:
gi|387397 **Mass:** 57807 **Score:** 85 **Matches:** 2(2) **Sequences:** 2(2)
epidermal keratin subunit I, partial [Mus musculus]
gi|12852157 **Mass:** 58587 **Score:** 85 **Matches:** 2(2) **Sequences:** 2(2)
unnamed protein product [Mus musculus]
gi|26349141 **Mass:** 52653 **Score:** 85 **Matches:** 2(2) **Sequences:** 2(2)
unnamed protein product [Mus musculus]
gi|26349459 **Mass:** 49471 **Score:** 85 **Matches:** 2(2) **Sequences:** 2(2)
unnamed protein product [Mus musculus]
gi|112983636 **Mass:** 57007 **Score:** 85 **Matches:** 2(2) **Sequences:** 2(2)
keratin, type I cytoskeletal 10 [Mus musculus]
gi|116242600 **Mass:** 57735 **Score:** 85 **Matches:** 2(2) **Sequences:** 2(2)
RecName: Full=Keratin, type I cytoskeletal 10; AltName: Full=56 kDa cytokeratin; AltName: Full=Cytokeratin-10; Short=CK-10; AltName: Full=Keratin, type I cytoskeletal 59 kDa; AltName: Full=Keratin-1[illegible]

---

4. gi|16716569 **Mass:** 26118 **Score:** 83 **Matches:** 2(1) **Sequences:** 1(1) **emPAI:** 0.13
protease, serine, 1 precursor [Mus musculus]

Query	Observed	Mr(expt)	Mr(calc)	Delta	Miss	Score	Expect	Rank	Unique	Peptide
15632	737.8900	2210.6482	2210.0967	0.5515	0	(44)	0.083	1	U	R.LGEHNINVLEGNEQFIDAAK.I
16412	1106.3871	2210.7596	2210.0967	0.6629	0	83	7.4e-006	1	U	R.LGEHNINVLEGNEQFIDAAK.I

---

5. gi|12843914 **Mass:** 29993 **Score:** 82 **Matches:** 1(1) **Sequences:** 1(1) **emPAI:** 0.11
unnamed protein product [Mus musculus]

Query	Observed	Mr(expt)	Mr(calc)	Delta	Miss	Score	Expect	Rank	Unique	Peptide
13984	651.6257	1301.2369	1301.7078	-0.4709	0	82	1.5e-005	1	U	R.SLDLDSIIAEVK.A

Proteins matching the same set of peptides:
gi|13272554 **Mass:** 42357 **Score:** 82 **Matches:** 1(1) **Sequences:** 1(1)
cytokeratin KRT2-6HF [Mus musculus]
gi|16303309 **Mass:** 61743 **Score:** 82 **Matches:** 1(1) **Sequences:** 1(1)
type II keratin 5 [Mus musculus]
gi|20911031 **Mass:** 61729 **Score:** 82 **Matches:** 1(1) **Sequences:** 1(1)
keratin, type II cytoskeletal 5 [Mus musculus]
gi|29789317 **Mass:** 59704 **Score:** 82 **Matches:** 1(1) **Sequences:** 1(1)
keratin, type II cytoskeletal 75 [Mus musculus]
gi|54607171 **Mass:** 59299 **Score:** 82 **Matches:** 1(1) **Sequences:** 1(1)
keratin, type II cytoskeletal 6A [Mus musculus]
gi|59798479 **Mass:** 60285 **Score:** 82 **Matches:** 1(1) **Sequences:** 1(1)
RecName: Full=Keratin, type II cytoskeletal 6B; AltName: Full=Cytokeratin-6B; Short=CK-6B; AltName: Full=Keratin-6-beta; Short=mK6-beta; AltName: Full=Keratin-6B; Short=K6B
gi|110645788 **Mass:** 60236 **Score:** 82 **Matches:** 1(1) **Sequences:** 1(1)
Krt6b protein [Mus musculus]
gi|113195684 **Mass:** 59490 **Score:** 82 **Matches:** 1(1) **Sequences:** 1(1)
keratin, type II cytoskeletal 6B [Mus musculus]
gi|116063325 **Mass:** 60154 **Score:** 82 **Matches:** 1(1) **Sequences:** 1(1)
Krt6b protein [Mus musculus]
gi|148672084 **Mass:** 34775 **Score:** 82 **Matches:** 1(1) **Sequences:** 1(1)
mCG17577 [Mus musculus]
gi|148672089 **Mass:** 62241 **Score:** 82 **Matches:** 1(1) **Sequences:** 1(1)
keratin 75 [Mus musculus]
gi|148672085 **Mass:** 38119 **Score:** 82 **Matches:** 1(1) **Sequences:** 1(1)
mCG144996 [Mus musculus]

---

6. gi|9910294 **Mass:** 57347 **Score:** 75 **Matches:** 2(2) **Sequences:** 2(2) **emPAI:** 0.12
keratin, type II cytoskeletal 71 [Mus musculus]

Query	Observed	Mr(expt)	Mr(calc)	Delta	Miss	Score	Expect	Rank	Unique	Peptide
11976	562.3885	1122.7624	1122.5557	0.2068	0	49	0.029	1		K.AEAEALYQTK.F
15642	738.2304	1474.4461	1474.7780	-0.3318	0	65	0.00053	1	U	R.FLEQQNQVLQTK.W

Proteins matching the same set of peptides:
gi|33146295 **Mass:** 57391 **Score:** 75 **Matches:** 2(2) **Sequences:** 2(2)
type II keratin [Mus musculus]
gi|148672081 **Mass:** 43429 **Score:** 75 **Matches:** 2(2) **Sequences:** 2(2)
mCG17589, isoform CRA_b [Mus musculus]

---

7. gi|2498741 **Mass:** 60964 **Score:** 66 **Matches:** 1(1) **Sequences:** 1(1) **emPAI:** 0.05
RecName: Full=Prolyl 4-hydroxylase subunit alpha-2; Short=4-PH alpha-2; AltName: Full=Procollagen-proline,2-oxoglutarate-4-dioxygenase subunit alpha-2; Flags: Precursor

Query	Observed	Mr(expt)	Mr(calc)	Delta	Miss	Score	Expect	Rank	Unique	Peptide
16157	785.1390	1568.2634	1568.6743	-0.4109	0	66	0.00051	1	U	R.QFFPTDEDESGAAR.A

Proteins matching the same set of peptides:
gi|148701596 **Mass:** 40313 **Score:** 66 **Matches:** 1(1) **Sequences:** 1(1)
procollagen-proline, 2-oxoglutarate 4-dioxygenase (proline 4-hydroxylase), alpha II polypeptide, isoform CRA_a [Mus musculus]
gi|148701597 **Mass:** 57417 **Score:** 66 **Matches:** 1(1) **Sequences:** 1(1)
procollagen-proline, 2-oxoglutarate 4-dioxygenase (proline 4-hydroxylase), alpha II polypeptide, isoform CRA_b [Mus musculus]
gi|148701599 **Mass:** 61292 **Score:** 66 **Matches:** 1(1) **Sequences:** 1(1)
procollagen-proline, 2-oxoglutarate 4-dioxygenase (proline 4-hydroxylase), alpha II polypeptide, isoform CRA_d [Mus musculus]
gi|148701600 **Mass:** 66870 **Score:** 66 **Matches:** 1(1) **Sequences:** 1(1)
procollagen-proline, 2-oxoglutarate 4-dioxygenase (proline 4-hydroxylase), alpha II polypeptide, isoform CRA_e [Mus musculus]
gi|209862961 **Mass:** 60774 **Score:** 66 **Matches:** 1(1) **Sequences:** 1(1)
prolyl 4-hydroxylase subunit alpha-2 isoform 1 precursor [Mus musculus]
gi|226874876 **Mass:** 60977 **Score:** 66 **Matches:** 1(1) **Sequences:** 1(1)
prolyl 4-hydroxylase subunit alpha-2 isoform 2 precursor [Mus musculus]

---

8. gi|148672076 **Mass:** 72140 **Score:** 65 **Matches:** 2(1) **Sequences:** 2(1) **emPAI:** 0.09

mCG17605, isoform CRA_b [Mus musculus]

Query	Observed	Mr(expt)	Mr(calc)	Delta	Miss	Score	Expect	Rank	Unique	Peptide
11007	521.2697	1040.5249	1040.5502	-0.0253	0	46	0.067	1	U	K.VDPEIQNVK.S
13545	628.0165	1254.0184	1253.6001	0.4184	0	65	0.0007	1	U	R.GFSSGSAVVSGGSR.R

Proteins matching the same set of peptides:

gi|398168 **Mass:** 70934 **Score:** 65 **Matches:** 2(1) **Sequences:** 2(1)
keratin 2 epidermis [Mus musculus]
gi|111308159 **Mass:** 70880 **Score:** 65 **Matches:** 2(1) **Sequences:** 2(1)
Keratin 2 [Mus musculus]
gi|124487419 **Mass:** 70880 **Score:** 65 **Matches:** 2(1) **Sequences:** 2(1)
keratin, type II cytoskeletal 2 epidermal [Mus musculus]
gi|148672075 **Mass:** 71783 **Score:** 65 **Matches:** 2(1) **Sequences:** 2(1)
mCG17605, isoform CRA_a [Mus musculus]

---

9. gi|51092303 **Mass:** 26514 **Score:** 65 **Matches:** 1(1) **Sequences:** 1(1) **emPAI:** 0.13
Try10-like trypsinogen precursor [Mus musculus]

Query	Observed	Mr(expt)	Mr(calc)	Delta	Miss	Score	Expect	Rank	Unique	Peptide
12639	588.3151	1174.6156	1174.6267	-0.0112	0	65	0.00087	1	U	K.TLDNDIMLIK.L

Proteins matching the same set of peptides:

gi|84781771 **Mass:** 26204 **Score:** 65 **Matches:** 1(1) **Sequences:** 1(1)
trypsin 10 precursor [Mus musculus]

---

10. gi|4159806 **Mass:** 65183 **Score:** 64 **Matches:** 1(1) **Sequences:** 1(1) **emPAI:** 0.05
type II keratin subunit protein [Mus musculus]

Query	Observed	Mr(expt)	Mr(calc)	Delta	Miss	Score	Expect	Rank	Unique	Peptide
15642	738.2304	1474.4461	1474.8144	-0.3682	1	64	0.0007	2	U	R.FLEQQNKVLQTK.W

---

11. gi|6166378 **Mass:** 84758 **Score:** 58 **Matches:** 1(1) **Sequences:** 1(1) **emPAI:** 0.04
growth suppressor 1L [Mus musculus]

Query	Observed	Mr(expt)	Mr(calc)	Delta	Miss	Score	Expect	Rank	Unique	Peptide
15783	747.1336	1492.2527	1491.7398	0.5129	0	58	0.0013	1	U	R.SPYNYLQVAYFK.I

Proteins matching the same set of peptides:

gi|6166380 **Mass:** 61560 **Score:** 58 **Matches:** 1(1) **Sequences:** 1(1)
growth suppressor 1S [Mus musculus]
gi|12846125 **Mass:** 84033 **Score:** 58 **Matches:** 1(1) **Sequences:** 1(1)
unnamed protein product [Mus musculus]
gi|23271416 **Mass:** 83540 **Score:** 58 **Matches:** 1(1) **Sequences:** 1(1)
Leprecan 1 [Mus musculus]
gi|26326437 **Mass:** 83476 **Score:** 58 **Matches:** 1(1) **Sequences:** 1(1)
unnamed protein product [Mus musculus]
gi|74205527 **Mass:** 84439 **Score:** 58 **Matches:** 1(1) **Sequences:** 1(1)
unnamed protein product [Mus musculus]
gi|109150433 **Mass:** 83598 **Score:** 58 **Matches:** 1(1) **Sequences:** 1(1)
prolyl 3-hydroxylase 1 isoform 2 precursor [Mus musculus]
gi|109150437 **Mass:** 84426 **Score:** 58 **Matches:** 1(1) **Sequences:** 1(1)
prolyl 3-hydroxylase 1 isoform 1 precursor [Mus musculus]
gi|148698509 **Mass:** 84378 **Score:** 58 **Matches:** 1(1) **Sequences:** 1(1)
leprecan 1, isoform CRA_a [Mus musculus]
gi|148698510 **Mass:** 83550 **Score:** 58 **Matches:** 1(1) **Sequences:** 1(1)
leprecan 1, isoform CRA_b [Mus musculus]
gi|148698511 **Mass:** 63605 **Score:** 58 **Matches:** 1(1) **Sequences:** 1(1)
leprecan 1, isoform CRA_c [Mus musculus]

---

12. gi|295841496 **Mass:** 14518 **Score:** 58 **Matches:** 2(2) **Sequences:** 1(1) **emPAI:** 0.23

tubulin beta 3 [Mus musculus]

Query	Observed	Mr(expt)	Mr(calc)	Delta	Miss	Score	Expect	Rank	Unique	Peptide
14141	660.4166	1318.8186	1318.6955	0.1231	0	52	0.016	1	U	R.IMNTFSVVPSPK.V 14142

Proteins matching the same set of peptides:

gi|202229 **Mass:** 42212 **Score:** 58 **Matches:** 2(2) **Sequences:** 1(1)
beta-tubulin, partial [Mus musculus]
gi|5174735 **Mass:** 49799 **Score:** 58 **Matches:** 2(2) **Sequences:** 1(1)
tubulin beta-4B chain [Homo sapiens]
gi|7106439 **Mass:** 49639 **Score:** 58 **Matches:** 2(2) **Sequences:** 1(1)
tubulin beta-5 chain [Mus musculus]
gi|12846758 **Mass:** 49608 **Score:** 58 **Matches:** 2(2) **Sequences:** 1(1)
unnamed protein product [Mus musculus]
gi|12851187 **Mass:** 49496 **Score:** 58 **Matches:** 2(2) **Sequences:** 1(1)
unnamed protein product [Mus musculus]
gi|12963615 **Mass:** 50386 **Score:** 58 **Matches:** 2(2) **Sequences:** 1(1)
tubulin beta-3 chain [Mus musculus]
gi|13542680 **Mass:** 49783 **Score:** 58 **Matches:** 2(2) **Sequences:** 1(1)
Tubulin, beta 2C [Mus musculus]
gi|21361322 **Mass:** 49554 **Score:** 58 **Matches:** 2(2) **Sequences:** 1(1)
tubulin beta-4A chain [Homo sapiens]
gi|26355849 **Mass:** 32238 **Score:** 58 **Matches:** 2(2) **Sequences:** 1(1)
unnamed protein product [Mus musculus]
gi|26356799 **Mass:** 34123 **Score:** 58 **Matches:** 2(2) **Sequences:** 1(1)
unnamed protein product [Mus musculus]
gi|74141821 **Mass:** 49667 **Score:** 58 **Matches:** 2(2) **Sequences:** 1(1)
unnamed protein product [Mus musculus]
gi|74144588 **Mass:** 49771 **Score:** 58 **Matches:** 2(2) **Sequences:** 1(1)
unnamed protein product [Mus musculus]
gi|74204140 **Mass:** 49616 **Score:** 58 **Matches:** 2(2) **Sequences:** 1(1)
unnamed protein product [Mus musculus]
gi|74212109 **Mass:** 49620 **Score:** 58 **Matches:** 2(2) **Sequences:** 1(1)
unnamed protein product [Mus musculus]
gi|74223737 **Mass:** 49652 **Score:** 58 **Matches:** 2(2) **Sequences:** 1(1)
unnamed protein product [Mus musculus]
gi|74223783 **Mass:** 50402 **Score:** 58 **Matches:** 2(2) **Sequences:** 1(1)
unnamed protein product [Mus musculus]
gi|148676266 **Mass:** 39342 **Score:** 58 **Matches:** 2(2) **Sequences:** 1(1)
mCG20287 [Mus musculus]
gi|148678613 **Mass:** 49970 **Score:** 58 **Matches:** 2(2) **Sequences:** 1(1)
mCG1424 [Mus musculus]
gi|148682710 **Mass:** 49784 **Score:** 58 **Matches:** 2(2) **Sequences:** 1(1)
mCG49614, isoform CRA_b [Mus musculus]
gi|148691289 **Mass:** 49910 **Score:** 58 **Matches:** 2(2) **Sequences:** 1(1)
tubulin, beta 5 [Mus musculus]

---

13. gi|438134 **Mass:** 123705 **Score:** 57 **Matches:** 1(1) **Sequences:** 1(1) **emPAI:** 0.03
DNA-directed DNA polymerase [Mus musculus]

Query	Observed	Mr(expt)	Mr(calc)	Delta	Miss	Score	Expect	Rank	Unique	Peptide
11720	553.2844	1104.5543	1104.6040	-0.0497	1	57	0.0047	1	U	R.RQGPGPGVPPK.R

Proteins matching the same set of peptides:

gi|2827903 **Mass:** 123657 **Score:** 57 **Matches:** 1(1) **Sequences:** 1(1)
DNA polymerase delta catalytic subunit [Mus musculus]
gi|14318657 **Mass:** 123666 **Score:** 57 **Matches:** 1(1) **Sequences:** 1(1)
Polymerase (DNA directed), delta 1, catalytic subunit [Mus musculus]
gi|26353290 **Mass:** 123698 **Score:** 57 **Matches:** 1(1) **Sequences:** 1(1)
unnamed protein product [Mus musculus]

gi|148690782 **Mass:** 125906 **Score:** 57 **Matches:** 1(1) **Sequences:** 1(1)
polymerase (DNA directed), delta 1, catalytic subunit, isoform CRA_a [Mus musculus]
gi|148690783 **Mass:** 90830 **Score:** 57 **Matches:** 1(1) **Sequences:** 1(1)
polymerase (DNA directed), delta 1, catalytic subunit, isoform CRA_b [Mus musculus]
gi|148690785 **Mass:** 122966 **Score:** 57 **Matches:** 1(1) **Sequences:** 1(1)
polymerase (DNA directed), delta 1, catalytic subunit, isoform CRA_d [Mus musculus]
gi|148690786 **Mass:** 123682 **Score:** 57 **Matches:** 1(1) **Sequences:** 1(1)
polymerase (DNA directed), delta 1, catalytic subunit, isoform CRA_e [Mus musculus]
gi|148690787 **Mass:** 107166 **Score:** 57 **Matches:** 1(1) **Sequences:** 1(1)
polymerase (DNA directed), delta 1, catalytic subunit, isoform CRA_f [Mus musculus]
gi|254587977 **Mass:** 123712 **Score:** 57 **Matches:** 1(1) **Sequences:** 1(1)
DNA polymerase delta catalytic subunit [Mus musculus]

---

14. gi|6680888 **Mass:** 25749 **Score:** 56 **Matches:** 1(1) **Sequences:** 1(1) **emPAI:** 0.13
CD63 antigen [Mus musculus]

Query	Observed	Mr(expt)	Mr(calc)	Delta	Miss	Score	Expect	Rank	Unique	Peptide
15128	708.0230	1414.0315	1413.6711	0.3604	0	56	0.0049	1	U	K.SFQQQMQNYLK.D

Proteins matching the same set of peptides:
gi|26389974 **Mass:** 26766 **Score:** 56 **Matches:** 1(1) **Sequences:** 1(1)
unnamed protein product [Mus musculus]
gi|74151897 **Mass:** 25777 **Score:** 56 **Matches:** 1(1) **Sequences:** 1(1)
unnamed protein product [Mus musculus]

---

15. gi|202210 **Mass:** 50134 **Score:** 55 **Matches:** 1(1) **Sequences:** 1(1) **emPAI:** 0.07
alpha-tubulin isotype M-alpha-2 [Mus musculus]

Query	Observed	Mr(expt)	Mr(calc)	Delta	Miss	Score	Expect	Rank	Unique	Peptide
10750	508.1963	1014.3781	1014.5709	-0.1928	0	55	0.004	1	U	K.DVNAAIATIK.T

Proteins matching the same set of peptides:
gi|202223 **Mass:** 22209 **Score:** 55 **Matches:** 1(1) **Sequences:** 1(1)
alpha-tubulin, partial [Mus musculus]
gi|202225 **Mass:** 39293 **Score:** 55 **Matches:** 1(1) **Sequences:** 1(1)
alpha-tubulin, partial [Mus musculus]
gi|6678465 **Mass:** 49928 **Score:** 55 **Matches:** 1(1) **Sequences:** 1(1)
tubulin alpha-3 chain [Mus musculus]
gi|6678469 **Mass:** 49877 **Score:** 55 **Matches:** 1(1) **Sequences:** 1(1)
tubulin alpha-1C chain [Mus musculus]
gi|6755901 **Mass:** 50104 **Score:** 55 **Matches:** 1(1) **Sequences:** 1(1)
tubulin alpha-1A chain [Mus musculus]
gi|12839396 **Mass:** 44021 **Score:** 55 **Matches:** 1(1) **Sequences:** 1(1)
unnamed protein product [Mus musculus]
gi|12850141 **Mass:** 38332 **Score:** 55 **Matches:** 1(1) **Sequences:** 1(1)
unnamed protein product [Mus musculus]
gi|13435888 **Mass:** 38746 **Score:** 55 **Matches:** 1(1) **Sequences:** 1(1)
Tuba1b protein [Mus musculus]
gi|34740335 **Mass:** 50120 **Score:** 55 **Matches:** 1(1) **Sequences:** 1(1)
tubulin alpha-1B chain [Mus musculus]
gi|53733821 **Mass:** 50120 **Score:** 55 **Matches:** 1(1) **Sequences:** 1(1)
Tubulin, alpha 1A [Mus musculus]
gi|74181454 **Mass:** 50104 **Score:** 55 **Matches:** 1(1) **Sequences:** 1(1)
unnamed protein product [Mus musculus]
gi|74186501 **Mass:** 50108 **Score:** 55 **Matches:** 1(1) **Sequences:** 1(1)
unnamed protein product [Mus musculus]
gi|74198443 **Mass:** 49907 **Score:** 55 **Matches:** 1(1) **Sequences:** 1(1)
unnamed protein product [Mus musculus]
gi|74198980 **Mass:** 50048 **Score:** 55 **Matches:** 1(1) **Sequences:** 1(1)
unnamed protein product [Mus musculus]
gi|74216624 **Mass:** 50116 **Score:** 55 **Matches:** 1(1) **Sequences:** 1(1)

unnamed protein product [Mus musculus]
gi|74220042 **Mass:** 50105 **Score:** 55 **Matches:** 1(1) **Sequences:** 1(1)
unnamed protein product [Mus musculus]
gi|148672205 **Mass:** 50888 **Score:** 55 **Matches:** 1(1) **Sequences:** 1(1)
mCG18413, isoform CRA_b [Mus musculus]
gi|148704842 **Mass:** 43956 **Score:** 55 **Matches:** 1(1) **Sequences:** 1(1)
mCG125663 [Mus musculus]

---

16. gi|3687320 **Mass:** 46137 **Score:** 54 **Matches:** 1(1) **Sequences:** 1(1) **emPAI:** 0.07
cartilage-associated protein (CASP) [Mus musculus]

Query	Observed	Mr(expt)	Mr(calc)	Delta	Miss	Score	Expect	Rank	Unique	Peptide
11191	**532.0100**	**1062.0053**	**1062.5458**	**-0.5405**	**0**	**54**	**0.0083**	**1**	**U**	**R.SVLADFQQR.E**

Proteins matching the same set of peptides:
gi|12857227 **Mass:** 46121 **Score:** 54 **Matches:** 1(1) **Sequences:** 1(1)
unnamed protein product [Mus musculus]
gi|148677365 **Mass:** 46110 **Score:** 54 **Matches:** 1(1) **Sequences:** 1(1)
mCG7119 [Mus musculus]
gi|225543173 **Mass:** 46140 **Score:** 54 **Matches:** 1(1) **Sequences:** 1(1)
cartilage-associated protein precursor [Mus musculus]

---

17. gi|85701680 **Mass:** 62806 **Score:** 53 **Matches:** 2(2) **Sequences:** 2(2) **emPAI:** 0.11
keratin, type II cytoskeletal 2 oral [Mus musculus]

Query	Observed	Mr(expt)	Mr(calc)	Delta	Miss	Score	Expect	Rank	Unique	Peptide
11227	**1065.4674**	**1064.4601**	**1064.5138**	**-0.0537**	**0**	**43**	**0.058**	**1**	**U**	**K.AQYEDIAQK.S**
11976	562.3885	1122.7624	1122.5557	0.2068	0	49	0.029	1		K.AEAEALYQTK.L

Proteins matching the same set of peptides:
gi|148672072 **Mass:** 63519 **Score:** 53 **Matches:** 2(2) **Sequences:** 2(2)
mCG144546 [Mus musculus]

---

18. gi|21489935 **Mass:** 52834 **Score:** 52 **Matches:** 2(2) **Sequences:** 2(2) **emPAI:** 0.13
keratin, type I cytoskeletal 14 [Mus musculus]

Query	Observed	Mr(expt)	Mr(calc)	Delta	Miss	Score	Expect	Rank	Unique	Peptide
10856	**515.0688**	**1028.1230**	**1028.5866**	**-0.4635**	**0**	**45**	**0.03**	**1**	**U**	**R.VLDELTLAR.A**
11545	546.0249	1090.0351	1089.5237	0.5115	0	46	0.023	1		K.VTMQNLNDR.L

Proteins matching the same set of peptides:
gi|148670626 **Mass:** 54668 **Score:** 52 **Matches:** 2(2) **Sequences:** 2(2)
mCG144006 [Mus musculus]
gi|741022 **Mass:** 49086 **Score:** 52 **Matches:** 2(2) **Sequences:** 2(2)
keratin 15
gi|904215 **Mass:** 49129 **Score:** 52 **Matches:** 2(2) **Sequences:** 2(2)
cytokeratin 15 [Mus musculus]
gi|83304265 **Mass:** 49107 **Score:** 52 **Matches:** 2(2) **Sequences:** 2(2)
RecName: Full=Keratin, type I cytoskeletal 15; AltName: Full=Cytokeratin-15; Short=CK-15; AltName: Full=Keratin-15; Short=K15
gi|226823220 **Mass:** 49463 **Score:** 52 **Matches:** 2(2) **Sequences:** 2(2)
keratin, type I cytoskeletal 15 [Mus musculus]

---

19. gi|468546 **Mass:** 57411 **Score:** 51 **Matches:** 1(1) **Sequences:** 1(1) **emPAI:** 0.06
CCT (chaperonin containing TCP-1) beta subunit [Mus musculus]

Query	Observed	Mr(expt)	Mr(calc)	Delta	Miss	Score	Expect	Rank	Unique	Peptide
16188	**791.9488**	**1581.8830**	**1581.9090**	**-0.0259**	**0**	**51**	**0.017**	**1**	**U**	**R.QVLLSAAEAAEVILR.V**

Proteins matching the same set of peptides:
gi|7670405 **Mass:** 52436 **Score:** 51 **Matches:** 1(1) **Sequences:** 1(1)
unnamed protein product [Mus musculus]

gi|126521835 **Mass:** 57441 **Score:** 51 **Matches:** 1(1) **Sequences:** 1(1)
T-complex protein 1 subunit beta [Mus musculus]

---

20. gi|6755893 **Score:** 50 **Matches:** 1(1) **Sequences:** 1(1) **emPAI:** 0.12
trypsin 4 precursor [Mus musculus]

Query	Observed	Mr(expt)	Mr(calc)	Delta	Miss	Score	Expect	Rank	Unique	Peptide
16412	1106.3871	2210.7596	2211.0920	-0.3323	0	50	0.017	2	U	R.LGEHNINVLEGNEQFVNSAK.I

Proteins matching the same set of peptides:
gi|51010909 **Score:** 50 **Matches:** 1(1) **Sequences:** 1(1)
gi|74203392 **Score:** 50 **Matches:** 1(1) **Sequences:** 1(1)

---

21. gi|27370092 **Mass:** 49477 **Score:** 50 **Matches:** 1(1) **Sequences:** 1(1) **emPAI:** 0.07
elongation factor Tu, mitochondrial isoform 1 [Mus musculus]

Query	Observed	Mr(expt)	Mr(calc)	Delta	Miss	Score	Expect	Rank	Unique	Peptide
12768	593.2388	1184.4630	1184.6149	-0.1519	0	50	0.027	1	U	R.AEAGDNLGALVR.G

Proteins matching the same set of peptides:
gi|148685430 **Mass:** 43302 **Score:** 50 **Matches:** 1(1) **Sequences:** 1(1)
mCG22399, isoform CRA_e [Mus musculus]
gi|148696761 **Mass:** 16047 **Score:** 50 **Matches:** 1(1) **Sequences:** 1(1)
mCG1048875, isoform CRA_a [Mus musculus]
gi|148696762 **Mass:** 12433 **Score:** 50 **Matches:** 1(1) **Sequences:** 1(1)
mCG1048875, isoform CRA_b [Mus musculus]
gi|148696763 **Mass:** 49507 **Score:** 50 **Matches:** 1(1) **Sequences:** 1(1)
mCG1048875, isoform CRA_c [Mus musculus]
gi|254911131 **Mass:** 47193 **Score:** 50 **Matches:** 1(1) **Sequences:** 1(1)
elongation factor Tu, mitochondrial isoform 2 [Mus musculus]
gi|148685429 **Mass:** 49613 **Score:** 50 **Matches:** 1(1) **Sequences:** 1(1)
mCG22399, isoform CRA_d [Mus musculus]

---

22. gi|293686 **Mass:** 59414 **Score:** 49 **Matches:** 1(1) **Sequences:** 1(1) **emPAI:** 0.06
epidermal keratin subunit II [Mus musculus]

Query	Observed	Mr(expt)	Mr(calc)	Delta	Miss	Score	Expect	Rank	Unique	Peptide
14931	698.0006	1393.9867	1393.7089	0.2778	1	49	0.025	1	U	R.TDAENEFVTLKK.D

---

23. gi|3212116 **Mass:** 16766 **Score:** 49 **Matches:** 1(1) **Sequences:** 1(1) **emPAI:** 0.20
prefoldin subunit 2 [Mus musculus]

Query	Observed	Mr(expt)	Mr(calc)	Delta	Miss	Score	Expect	Rank	Unique	Peptide
14659	686.9688	1371.9231	1370.7769	1.1462	0	49	0.014	1	U	K.IIETLSQQLQAK.G

Proteins matching the same set of peptides:
gi|31981577 **Mass:** 16524 **Score:** 49 **Matches:** 1(1) **Sequences:** 1(1)
prefoldin subunit 2 [Mus musculus]
gi|148707143 **Mass:** 11754 **Score:** 49 **Matches:** 1(1) **Sequences:** 1(1)
prefoldin 2, isoform CRA_b [Mus musculus]

---

24. gi|193645 **Mass:** 18932 **Score:** 46 **Matches:** 1(1) **Sequences:** 1(1) **emPAI:** 0.18
glucose-regulated protein 78, partial [Mus musculus]

Query	Observed	Mr(expt)	Mr(calc)	Delta	Miss	Score	Expect	Rank	Unique	Peptide
14961	699.5220	1397.0295	1396.7813	0.2482	0	46	0.041	1	U	K.ELEEIVQPIISK.L

Proteins matching the same set of peptides:
gi|387113 **Mass:** 16164 **Score:** 46 **Matches:** 1(1) **Sequences:** 1(1)
immunoglobulin heavy chain binding protein, partial [Mus musculus]
gi|1304157 **Mass:** 72412 **Score:** 46 **Matches:** 1(1) **Sequences:** 1(1)
78 kDa glucose-regulated protein [Mus musculus]

gi|2598562 **Mass:** 72433 **Score:** 46 **Matches:** 1(1) **Sequences:** 1(1)
BiP [Mus musculus]
gi|12835845 **Mass:** 72378 **Score:** 46 **Matches:** 1(1) **Sequences:** 1(1)
unnamed protein product [Mus musculus]
gi|74188814 **Mass:** 72305 **Score:** 46 **Matches:** 1(1) **Sequences:** 1(1)
unnamed protein product [Mus musculus]
gi|74198293 **Mass:** 72419 **Score:** 46 **Matches:** 1(1) **Sequences:** 1(1)
unnamed protein product [Mus musculus]
gi|74198974 **Mass:** 72361 **Score:** 46 **Matches:** 1(1) **Sequences:** 1(1)
unnamed protein product [Mus musculus]
gi|74207492 **Mass:** 72301 **Score:** 46 **Matches:** 1(1) **Sequences:** 1(1)
unnamed protein product [Mus musculus]
gi|74225394 **Mass:** 72337 **Score:** 46 **Matches:** 1(1) **Sequences:** 1(1)
unnamed protein product [Mus musculus]
gi|148676670 **Mass:** 56257 **Score:** 46 **Matches:** 1(1) **Sequences:** 1(1)
heat shock 70kD protein 5 (glucose-regulated protein), isoform CRA_b [Mus musculus]
gi|254540166 **Mass:** 72377 **Score:** 46 **Matches:** 1(1) **Sequences:** 1(1)
78 kDa glucose-regulated protein precursor [Mus musculus]

---

25. gi|53830774 **Mass:** 34092 **Score:** 46 **Matches:** 1(1) **Sequences:** 1(1) **emPAI:** 0.10
lactase [Mus musculus]

Query	Observed	Mr(expt)	Mr(calc)	Delta	Miss	Score	Expect	Rank	Unique	Peptide
11588	548.0338	1094.0531	1094.5132	-0.4600	0	46	0.018	1	U	R.LPEFTESEK.K

Proteins matching the same set of peptides:
gi|74192292 **Mass:** 139170 **Score:** 46 **Matches:** 1(1) **Sequences:** 1(1)
unnamed protein product [Mus musculus]
gi|124487297 **Mass:** 217665 **Score:** 46 **Matches:** 1(1) **Sequences:** 1(1)
lactase-phlorizin hydrolase preproprotein [Mus musculus]
gi|148707805 **Mass:** 217693 **Score:** 46 **Matches:** 1(1) **Sequences:** 1(1)
mCG128560 [Mus musculus]

---

26. gi|81872882 **Mass:** 242989 **Score:** 46 **Matches:** 1(1) **Sequences:** 1(1) **emPAI:** 0.01
RecName: Full=Adenomatous polyposis coli protein 2

Query	Observed	Mr(expt)	Mr(calc)	Delta	Miss	Score	Expect	Rank	Unique	Peptide
15985	762.1919	2283.5538	2284.1344	-0.5806	1	46	0.042	1	U	K.YQAAAMAVSPGTCVPSLYVRK.Q

Proteins matching the same set of peptides:
gi|117938322 **Mass:** 242963 **Score:** 46 **Matches:** 1(1) **Sequences:** 1(1)
adenomatous polyposis coli protein 2 [Mus musculus]

---

27. gi|191765 **Mass:** 47195 **Score:** 45 **Matches:** 1(0) **Sequences:** 1(0) **emPAI:** 0.07
alpha-fetoprotein, partial [Mus musculus]

Query	Observed	Mr(expt)	Mr(calc)	Delta	Miss	Score	Expect	Rank	Unique	Peptide
15680	740.7523	1479.4900	1478.7881	0.7018	0	45	0.065	1	U	K.LGEYGFQNAILVR.Y

Proteins matching the same set of peptides:
gi|26340966 **Mass:** 68678 **Score:** 45 **Matches:** 1(0) **Sequences:** 1(0)
unnamed protein product [Mus musculus]
gi|26341396 **Mass:** 64961 **Score:** 45 **Matches:** 1(0) **Sequences:** 1(0)
unnamed protein product [Mus musculus]
gi|74137565 **Mass:** 68688 **Score:** 45 **Matches:** 1(0) **Sequences:** 1(0)
unnamed protein product [Mus musculus]
gi|163310765 **Mass:** 68648 **Score:** 45 **Matches:** 1(0) **Sequences:** 1(0)
serum albumin precursor [Mus musculus]

---

28. gi|54777 **Mass:** 57108 **Score:** 45 **Matches:** 1(0) **Sequences:** 1(0) **emPAI:** 0.06
unnamed protein product [Mus musculus]

Query	Observed	Mr(expt)	Mr(calc)	Delta	Miss	Score	Expect	Rank	Unique	Peptide
13901	647.1276	1292.2406	1292.5918	-0.3512	0	45	0.055	1	U	K.MDSTANEVEAVK.V

Proteins matching the same set of peptides:

gi|42415475 **Mass:** 57023 **Score:** 45 **Matches:** 1(0) **Sequences:** 1(0)
protein disulfide-isomerase precursor [Mus musculus]
gi|74138891 **Mass:** 56951 **Score:** 45 **Matches:** 1(0) **Sequences:** 1(0)
unnamed protein product [Mus musculus]
gi|74141920 **Mass:** 57053 **Score:** 45 **Matches:** 1(0) **Sequences:** 1(0)
unnamed protein product [Mus musculus]
gi|74178069 **Mass:** 57099 **Score:** 45 **Matches:** 1(0) **Sequences:** 1(0)
unnamed protein product [Mus musculus]
gi|74190076 **Mass:** 56963 **Score:** 45 **Matches:** 1(0) **Sequences:** 1(0)
unnamed protein product [Mus musculus]
gi|74191500 **Mass:** 57022 **Score:** 45 **Matches:** 1(0) **Sequences:** 1(0)
unnamed protein product [Mus musculus]
gi|74198312 **Mass:** 57051 **Score:** 45 **Matches:** 1(0) **Sequences:** 1(0)
unnamed protein product [Mus musculus]
gi|74198632 **Mass:** 16308 **Score:** 45 **Matches:** 1(0) **Sequences:** 1(0)
unnamed protein product [Mus musculus]
gi|74198706 **Mass:** 57000 **Score:** 45 **Matches:** 1(0) **Sequences:** 1(0)
unnamed protein product [Mus musculus]
gi|74203945 **Mass:** 56566 **Score:** 45 **Matches:** 1(0) **Sequences:** 1(0)
unnamed protein product [Mus musculus]
gi|74212231 **Mass:** 57053 **Score:** 45 **Matches:** 1(0) **Sequences:** 1(0)
unnamed protein product [Mus musculus]
gi|74219772 **Mass:** 57008 **Score:** 45 **Matches:** 1(0) **Sequences:** 1(0)
unnamed protein product [Mus musculus]
gi|74220649 **Mass:** 57067 **Score:** 45 **Matches:** 1(0) **Sequences:** 1(0)
unnamed protein product [Mus musculus]
gi|148702818 **Mass:** 58984 **Score:** 45 **Matches:** 1(0) **Sequences:** 1(0)
prolyl 4-hydroxylase, beta polypeptide, isoform CRA_a [Mus musculus]
gi|148702819 **Mass:** 61492 **Score:** 45 **Matches:** 1(0) **Sequences:** 1(0)
prolyl 4-hydroxylase, beta polypeptide, isoform CRA_b [Mus musculus]

---

29. gi|26390362 **Mass:** 39663 **Score:** 45 **Matches:** 1(0) **Sequences:** 1(0) **emPAI:** 0.08
unnamed protein product [Mus musculus]

Query	Observed	Mr(expt)	Mr(calc)	Delta	Miss	Score	Expect	Rank	Unique	Peptide
12452	581.0289	1160.0432	1159.5622	0.4810	0	45	0.068	1	U	R.EGWTVQDVAR.V

Proteins matching the same set of peptides:

gi|148287013 **Mass:** 39693 **Score:** 45 **Matches:** 1(0) **Sequences:** 1(0)
paraneoplastic antigen Ma1 homolog [Mus musculus]

Mascot: http://www.matrixscience.com/

PFHR9

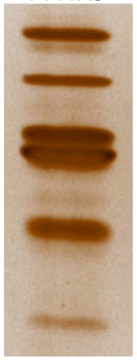

5

{MATRIX SCIENCE} Mascot Search Results

```
User                    :
Email                   :
Search title            :
MS data file            : ZIEN-29Nov2017_5m.mgf
Database                : NCBInr 20120419 (17893860 sequences; 6141683785 residues)
Taxonomy                : Mus musculus (house mouse) (138831 sequences)
Timestamp               : 30 Nov 2017 at 21:31:00 GMT
Enzyme                  : Trypsin
Variable modifications  : Carbamidomethyl (C),Oxidation (M),Oxidation (P)
Mass values             : Monoisotopic
Protein Mass            : Unrestricted
Peptide Mass Tolerance  : ± 1.25 Da
Fragment Mass Tolerance : ± 1.001 Da
Max Missed Cleavages    : 1
Instrument type         : Default
Number of queries       : 15860
Protein hits            : gi|4159806  type II keratin subunit protein [Mus musculus]
                          gi|46485130 TPA_exp: keratin Kb40 [Mus musculus]
                          gi|12859782 unnamed protein product [Mus musculus]
                          gi|47523977 keratin, type II cytoskeletal 72 [Mus musculus]
                          gi|387397   epidermal keratin subunit I, partial [Mus musculus]
                          gi|2498741  RecName: Full=Prolyl 4-hydroxylase subunit alpha-2; Short=4-PH alpha-2; AltName: Full=Procollagen-proline,2-oxoglutarate-4-dioxygenase subunit alpha-2; Flags: Precursor
                          gi|16303309 type II keratin 5 [Mus musculus]
                          gi|1389682  plakoglobin, partial [Mus musculus]
                          gi|16716569 protease, serine, 1 precursor [Mus musculus]
                          gi|26337253 unnamed protein product [Mus musculus]
                          gi|12846468 unnamed protein product [Mus musculus]
                          gi|6755893  trypsin 4 precursor [Mus musculus]
                          gi|3687320  cartilage-associated protein (CASP) [Mus musculus]
                          gi|26336999 unnamed protein product [Mus musculus]
                          gi|53830774 lactase [Mus musculus]
                          gi|51450    heat shock protein [Mus musculus]
                          gi|52789    unnamed protein product [Mus musculus]
                          gi|21489937 lanC-like protein 1 [Mus musculus]
                          gi|12859178 unnamed protein product [Mus musculus]
                          gi|19353667 Pcmtd2 protein, partial [Mus musculus]
```

Select Summary Report

Format As | Select Summary (protein hits) | Help

Significance threshold p< 0.05 | Max. number of hits AUTO

Standard scoring ○ MudPIT scoring ◉ Ions score or expect cut-off 43 | Show sub-sets 0

Show pop-ups ◉ Suppress pop-ups ○ | Require bold red ☐

Re-Search ◉ All queries ○ Unassigned ○ Below homology threshold ○ Below identity threshold

1. gi|4159806 **Mass:** 65183 **Score:** 178 **Matches:** 5(5) **Sequences:** 2(2) **emPAI:** 0.10
type II keratin subunit protein [Mus musculus]

Query	Observed	Mr(expt)	Mr(calc)	Delta	Miss	Score	Expect	Rank	Unique	Peptide
14220	692.5523	1383.0901	1383.7034	-0.6133	1	49	0.022	1		K.SLNDKFASFIDK.V
15069	738.1921	1474.3697	1474.8144	-0.4446	1	81	1.3e-005	1	U	R.FLEQQNKVLQTK.W 15077 15082 15083

2. gi|46485130 **Mass:** 85186 **Score:** 171 **Matches:** 6(6) **Sequences:** 2(2) **emPAI:** 0.08
TPA_exp: keratin Kb40 [Mus musculus]

Query	Observed	Mr(expt)	Mr(calc)	Delta	Miss	Score	Expect	Rank	Unique	Peptide
15082	738.6157	1475.2169	1475.7984	-0.5815	1	85	2.5e-006	1	U	R.FLEQQNKVLETK.W 15077 15083 15090 15094
15719	820.4082	1638.8018	1637.8525	0.9493	1	55	0.0075	1	U	R.SLNNQFASFIDKVR.F

Proteins matching the same set of peptides:
gi|111185567 **Mass:** 54730 **Score:** 171 **Matches:** 6(6) **Sequences:** 2(2)
Krt78 protein [Mus musculus]
gi|111185722 **Mass:** 56744 **Score:** 171 **Matches:** 6(6) **Sequences:** 2(2)
Krt78 protein [Mus musculus]
gi|119850791 **Mass:** 54739 **Score:** 171 **Matches:** 6(6) **Sequences:** 2(2)
Krt78 protein [Mus musculus]
gi|145580629 **Mass:** 112194 **Score:** 171 **Matches:** 6(6) **Sequences:** 2(2)
keratin Kb40 [Mus musculus]

---

3. gi|12859782 **Mass:** 65586 **Score:** 152 **Matches:** 5(5) **Sequences:** 2(2) **emPAI:** 0.10
unnamed protein product [Mus musculus]

Query	Observed	Mr(expt)	Mr(calc)	Delta	Miss	Score	Expect	Rank	Unique	Peptide
14220	692.5523	1383.0901	1383.7034	-0.6133	1	49	0.022	1		K.SLNDKFASFIDK.V
15082	738.6157	1475.2169	1474.7780	0.4389	0	81	7.5e-006	3	U	R.FLEQQNQVLQTK.W 15069 15077 15083

Proteins matching the same set of peptides:
gi|126116585 **Mass:** 65565 **Score:** 152 **Matches:** 5(5) **Sequences:** 2(2)
keratin, type II cytoskeletal 1 [Mus musculus]

---

4. gi|47523977 **Mass:** 56715 **Score:** 145 **Matches:** 5(5) **Sequences:** 1(1) **emPAI:** 0.06
keratin, type II cytoskeletal 72 [Mus musculus]

Query	Observed	Mr(expt)	Mr(calc)	Delta	Miss	Score	Expect	Rank	Unique	Peptide
15082	738.6157	1475.2169	1475.7620	-0.5451	0	80	7.9e-006	4	U	R.FLEQQNQVLETK.W 15077 15083 15090 15094

---

5. gi|387397 **Mass:** 57807 **Score:** 122 **Matches:** 3(3) **Sequences:** 2(2) **emPAI:** 0.12
epidermal keratin subunit I, partial [Mus musculus]

Query	Observed	Mr(expt)	Mr(calc)	Delta	Miss	Score	Expect	Rank	Unique	Peptide
14203	**691.6326**	**1381.2506**	**1380.6408**	**0.6097**	**0**	**81**	**1.7e-005**	**1**	**U**	**R.ALEESNYELEGK.I**
14287	**696.0388**	**1390.0630**	**1389.6736**	**0.3894**	**0**	**72**	**0.00013**	**1**	**U**	**K.QSLEASLAETEGR.Y** 14292

Proteins matching the same set of peptides:
gi|12852157 **Mass:** 58587 **Score:** 122 **Matches:** 3(3) **Sequences:** 2(2)
unnamed protein product [Mus musculus]
gi|26349141 **Mass:** 52653 **Score:** 122 **Matches:** 3(3) **Sequences:** 2(2)
unnamed protein product [Mus musculus]
gi|26349459 **Mass:** 49471 **Score:** 122 **Matches:** 3(3) **Sequences:** 2(2)
unnamed protein product [Mus musculus]
gi|112983636 **Mass:** 57007 **Score:** 122 **Matches:** 3(3) **Sequences:** 2(2)
keratin, type I cytoskeletal 10 [Mus musculus]
gi|116242600 **Mass:** 57735 **Score:** 122 **Matches:** 3(3) **Sequences:** 2(2)
RecName: Full=Keratin, type I cytoskeletal 10; AltName: Full=56 kDa cytokeratin; AltName: Full=Cytokeratin-10; Short=CK-10; AltName: Full=Keratin, type I cytoskeletal 59 kDa; AltName: Full=Keratin-1[illegible]

---

6. gi|2498741 **Mass:** 60964 **Score:** 74 **Matches:** 1(1) **Sequences:** 1(1) **emPAI:** 0.05
RecName: Full=Prolyl 4-hydroxylase subunit alpha-2; Short=4-PH alpha-2; AltName: Full=Procollagen-proline,2-oxoglutarate-4-dioxygenase subunit alpha-2; Flags: Precursor

Query	Observed	Mr(expt)	Mr(calc)	Delta	Miss	Score	Expect	Rank	Unique	Peptide
10969	**533.6311**	**1065.2477**	**1065.5819**	**-0.3342**	**0**	**74**	**3.2e-005**	**1**	**U**	**K.TGVLTVASYR.V**

Proteins matching the same set of peptides:
gi|74216495 **Mass:** 21055 **Score:** 74 **Matches:** 1(1) **Sequences:** 1(1)
unnamed protein product [Mus musculus]
gi|148701597 **Mass:** 57417 **Score:** 74 **Matches:** 1(1) **Sequences:** 1(1)
procollagen-proline, 2-oxoglutarate 4-dioxygenase (proline 4-hydroxylase), alpha II polypeptide, isoform CRA_b [Mus musculus]
gi|148701598 **Mass:** 21273 **Score:** 74 **Matches:** 1(1) **Sequences:** 1(1)
procollagen-proline, 2-oxoglutarate 4-dioxygenase (proline 4-hydroxylase), alpha II polypeptide, isoform CRA_c [Mus musculus]
gi|148701599 **Mass:** 61292 **Score:** 74 **Matches:** 1(1) **Sequences:** 1(1)
procollagen-proline, 2-oxoglutarate 4-dioxygenase (proline 4-hydroxylase), alpha II polypeptide, isoform CRA_d [Mus musculus]

gi|148701600 **Mass:** 66870 **Score:** 74 **Matches:** 1(1) **Sequences:** 1(1)
procollagen-proline, 2-oxoglutarate 4-dioxygenase (proline 4-hydroxylase), alpha II polypeptide, isoform CRA_e [Mus musculus]
gi|209862961 **Mass:** 60774 **Score:** 74 **Matches:** 1(1) **Sequences:** 1(1)
prolyl 4-hydroxylase subunit alpha-2 isoform 1 precursor [Mus musculus]
gi|226874876 **Mass:** 60977 **Score:** 74 **Matches:** 1(1) **Sequences:** 1(1)
prolyl 4-hydroxylase subunit alpha-2 isoform 2 precursor [Mus musculus]

---

7. gi|16303309 **Mass:** 61743 **Score:** 73 **Matches:** 2(2) **Sequences:** 2(2) **emPAI:** 0.11
type II keratin 5 [Mus musculus]

Query	Observed	Mr(expt)	Mr(calc)	Delta	Miss	Score	Expect	Rank	Unique	Peptide
13007	621.5424	1241.0702	1241.5564	-0.4862	0	53	0.01	1	U	R.TEAESWYQTK.Y
14648	713.6679	1425.3211	1424.6680	0.6532	0	57	0.0037	1	U	R.VDALMDEINFMK.M

Proteins matching the same set of peptides:
gi|20911031 **Mass:** 61729 **Score:** 73 **Matches:** 2(2) **Sequences:** 2(2)
keratin, type II cytoskeletal 5 [Mus musculus]

---

8. gi|1389682 **Mass:** 68068 **Score:** 73 **Matches:** 1(1) **Sequences:** 1(1) **emPAI:** 0.05
plakoglobin, partial [Mus musculus]

Query	Observed	Mr(expt)	Mr(calc)	Delta	Miss	Score	Expect	Rank	Unique	Peptide
15139	742.2332	1482.4517	1482.7467	-0.2949	0	73	8.8e-005	1	U	R.NEGTATYAAAVLFR.I

Proteins matching the same set of peptides:
gi|28395018 **Mass:** 81749 **Score:** 73 **Matches:** 1(1) **Sequences:** 1(1)
junction plakoglobin [Mus musculus]

---

9. gi|16716569 **Mass:** 26118 **Score:** 65 **Matches:** 2(2) **Sequences:** 1(1) **emPAI:** 0.13
protease, serine, 1 precursor [Mus musculus]

Query	Observed	Mr(expt)	Mr(calc)	Delta	Miss	Score	Expect	Rank	Unique	Peptide
15061	738.0139	2211.0197	2210.0967	0.9230	0	59	0.0026	1	U	R.LGEHNINVLEGNEQFIDAAK.I 15060

---

10. gi|26337253 **Mass:** 53417 **Score:** 63 **Matches:** 1(1) **Sequences:** 1(1) **emPAI:** 0.06
unnamed protein product [Mus musculus]

Query	Observed	Mr(expt)	Mr(calc)	Delta	Miss	Score	Expect	Rank	Unique	Peptide
14805	722.0333	1442.0520	1441.7235	0.3285	0	63	0.0012	1	U	K.GPAGVELNSMQPVK.E

Proteins matching the same set of peptides:
gi|71060037 **Mass:** 53416 **Score:** 63 **Matches:** 1(1) **Sequences:** 1(1)
Slc2a3 [Mus musculus]
gi|261862282 **Mass:** 53444 **Score:** 63 **Matches:** 1(1) **Sequences:** 1(1)
solute carrier family 2, facilitated glucose transporter member 3 [Mus musculus]

---

11. gi|12846468 **Mass:** 40872 **Score:** 58 **Matches:** 1(1) **Sequences:** 1(1) **emPAI:** 0.08
unnamed protein product [Mus musculus]

Query	Observed	Mr(expt)	Mr(calc)	Delta	Miss	Score	Expect	Rank	Unique	Peptide
14387	700.5706	1399.1266	1398.7983	0.3283	0	58	0.0028	1	U	K.LLAAFPNLANLSR.T

Proteins matching the same set of peptides:
gi|26341012 **Mass:** 38517 **Score:** 58 **Matches:** 1(1) **Sequences:** 1(1)
unnamed protein product [Mus musculus]
gi|26343781 **Mass:** 33610 **Score:** 58 **Matches:** 1(1) **Sequences:** 1(1)
unnamed protein product [Mus musculus]
gi|26351471 **Mass:** 49011 **Score:** 58 **Matches:** 1(1) **Sequences:** 1(1)
unnamed protein product [Mus musculus]
gi|50510431 **Mass:** 45578 **Score:** 58 **Matches:** 1(1) **Sequences:** 1(1)
mKIAA0265 protein [Mus musculus]
gi|58037463 **Mass:** 48981 **Score:** 58 **Matches:** 1(1) **Sequences:** 1(1)

kelch domain-containing protein 10 [Mus musculus]
gi|62945268 **Mass:** 45351 **Score:** 58 **Matches:** 1(1) **Sequences:** 1(1)
kelch domain-containing protein 10 [Rattus norvegicus]
gi|148681799 **Mass:** 33511 **Score:** 58 **Matches:** 1(1) **Sequences:** 1(1)
RIKEN cDNA 2410127E18, isoform CRA_a [Mus musculus]
gi|148681802 **Mass:** 32113 **Score:** 58 **Matches:** 1(1) **Sequences:** 1(1)
RIKEN cDNA 2410127E18, isoform CRA_c [Mus musculus]

---

12. gi|6755893 **Mass:** 26257 **Score:** 57 **Matches:** 1(1) **Sequences:** 1(1) **emPAI:** 0.13
trypsin 4 precursor [Mus musculus]

Query	Observed	Mr(expt)	Mr(calc)	Delta	Miss	Score	Expect	Rank	Unique	Peptide
15071	738.3822	2212.1248	2211.0920	1.0328	0	57	0.0052	1	U	R.LGEHNINVLEGNEQFVNSAK.I

Proteins matching the same set of peptides:
gi|51010909 **Mass:** 26260 **Score:** 57 **Matches:** 1(1) **Sequences:** 1(1)
trypsin 5 precursor [Mus musculus]
gi|74203392 **Mass:** 27146 **Score:** 57 **Matches:** 1(1) **Sequences:** 1(1)
unnamed protein product [Mus musculus]

---

13. gi|3687320 **Mass:** 46137 **Score:** 57 **Matches:** 2(2) **Sequences:** 2(2) **emPAI:** 0.15
cartilage-associated protein (CASP) [Mus musculus]

Query	Observed	Mr(expt)	Mr(calc)	Delta	Miss	Score	Expect	Rank	Unique	Peptide
13636	662.2533	1322.4920	1322.6176	-0.1256	0	47	0.045	1	U	R.DELMPLESAYR.H
15767	840.3280	1678.6414	1678.8488	-0.2073	0	47	0.043	1	U	R.TSISDMELALPDFLK.A

Proteins matching the same set of peptides:
gi|12857227 **Mass:** 46121 **Score:** 57 **Matches:** 2(2) **Sequences:** 2(2)
unnamed protein product [Mus musculus]
gi|148677365 **Mass:** 46110 **Score:** 57 **Matches:** 2(2) **Sequences:** 2(2)
mCG7119 [Mus musculus]
gi|225543173 **Mass:** 46140 **Score:** 57 **Matches:** 2(2) **Sequences:** 2(2)
cartilage-associated protein precursor [Mus musculus]

---

14. gi|26336999 **Mass:** 60848 **Score:** 55 **Matches:** 1(1) **Sequences:** 1(1) **emPAI:** 0.05
unnamed protein product [Mus musculus]

Query	Observed	Mr(expt)	Mr(calc)	Delta	Miss	Score	Expect	Rank	Unique	Peptide
15096	739.4654	2215.3743	2215.1274	0.2470	1	55	0.006	1	U	R.LTSTATKDPEGFVGHPVNAFK.L

Proteins matching the same set of peptides:
gi|33859596 **Mass:** 60872 **Score:** 55 **Matches:** 1(1) **Sequences:** 1(1)
prolyl 4-hydroxylase subunit alpha-1 precursor [Mus musculus]
gi|74148153 **Mass:** 51722 **Score:** 55 **Matches:** 1(1) **Sequences:** 1(1)
unnamed protein product [Mus musculus]
gi|74224984 **Mass:** 60886 **Score:** 55 **Matches:** 1(1) **Sequences:** 1(1)
unnamed protein product [Mus musculus]
gi|74225936 **Mass:** 63769 **Score:** 55 **Matches:** 1(1) **Sequences:** 1(1)
unnamed protein product [Mus musculus]

---

15. gi|53830774 **Mass:** 34092 **Score:** 49 **Matches:** 1(1) **Sequences:** 1(1) **emPAI:** 0.10
lactase [Mus musculus]

Query	Observed	Mr(expt)	Mr(calc)	Delta	Miss	Score	Expect	Rank	Unique	Peptide
11285	547.7469	1093.4792	1094.5132	-1.0339	0	49	0.014	1	U	R.LPEFTESEK.K

Proteins matching the same set of peptides:
gi|74192292 **Mass:** 139170 **Score:** 49 **Matches:** 1(1) **Sequences:** 1(1)
unnamed protein product [Mus musculus]
gi|124487297 **Mass:** 217665 **Score:** 49 **Matches:** 1(1) **Sequences:** 1(1)

lactase-phlorizin hydrolase preproprotein [Mus musculus]
gi|148707805 **Mass:** 217693 **Score:** 49 **Matches:** 1(1) **Sequences:** 1(1)
mCG128560 [Mus musculus]

---

16. gi|51450 **Mass:** 46560 **Score:** 48 **Matches:** 1(1) **Sequences:** 1(1) **emPAI:** 0.07
heat shock protein [Mus musculus]

Query	Observed	Mr(expt)	Mr(calc)	Delta	Miss	Score	Expect	Rank	Unique	Peptide
12831	613.0960	1224.1775	1223.6510	0.5265	0	48	0.012	1	U	K.GVVEVTHDLQK.H

Proteins matching the same set of peptides:
gi|200966 **Mass:** 45623 **Score:** 48 **Matches:** 1(1) **Sequences:** 1(1)
put. serine protease inhibitor [Mus musculus]
gi|26345418 **Mass:** 46481 **Score:** 48 **Matches:** 1(1) **Sequences:** 1(1)
unnamed protein product [Mus musculus]
gi|26348007 **Mass:** 46490 **Score:** 48 **Matches:** 1(1) **Sequences:** 1(1)
unnamed protein product [Mus musculus]
gi|74139266 **Mass:** 46490 **Score:** 48 **Matches:** 1(1) **Sequences:** 1(1)
unnamed protein product [Mus musculus]
gi|74191337 **Mass:** 46505 **Score:** 48 **Matches:** 1(1) **Sequences:** 1(1)
unnamed protein product [Mus musculus]
gi|74198254 **Mass:** 46500 **Score:** 48 **Matches:** 1(1) **Sequences:** 1(1)
unnamed protein product [Mus musculus]
gi|148684430 **Mass:** 44954 **Score:** 48 **Matches:** 1(1) **Sequences:** 1(1)
serine (or cysteine) peptidase inhibitor, clade H, member 1, isoform CRA_a [Mus musculus]
gi|161353502 **Mass:** 46504 **Score:** 48 **Matches:** 1(1) **Sequences:** 1(1)
serpin H1 precursor [Mus musculus]

---

17. gi|52789 **Mass:** 54415 **Score:** 45 **Matches:** 1(1) **Sequences:** 1(1) **emPAI:** 0.06
unnamed protein product [Mus musculus]

Query	Observed	Mr(expt)	Mr(calc)	Delta	Miss	Score	Expect	Rank	Unique	Peptide
14209	692.0989	1382.1832	1382.7194	-0.5362	1	45	0.058	1	U	K.SLNNKFASFIDK.V

Proteins matching the same set of peptides:
gi|309215 **Mass:** 53210 **Score:** 45 **Matches:** 1(1) **Sequences:** 1(1)
EndoA' cytokeratin (5' end put.); putative [Mus musculus]
gi|511654 **Mass:** 54220 **Score:** 45 **Matches:** 1(1) **Sequences:** 1(1)
keratin type II [Mus musculus]
gi|74177777 **Mass:** 54546 **Score:** 45 **Matches:** 1(1) **Sequences:** 1(1)
unnamed protein product [Mus musculus]
gi|74219975 **Mass:** 54459 **Score:** 45 **Matches:** 1(1) **Sequences:** 1(1)
unnamed protein product [Mus musculus]
gi|76779293 **Mass:** 54514 **Score:** 45 **Matches:** 1(1) **Sequences:** 1(1)
Keratin 8 [Mus musculus]
gi|114145561 **Mass:** 54531 **Score:** 45 **Matches:** 1(1) **Sequences:** 1(1)
keratin, type II cytoskeletal 8 [Mus musculus]

---

18. gi|21489937 **Mass:** 45312 **Score:** 45 **Matches:** 1(1) **Sequences:** 1(1) **emPAI:** 0.07
lanC-like protein 1 [Mus musculus]

Query	Observed	Mr(expt)	Mr(calc)	Delta	Miss	Score	Expect	Rank	Unique	Peptide
10769	524.0907	1046.1668	1045.5226	0.6442	0	45	0.029	1	U	R.ELLQQMER.G

Proteins matching the same set of peptides:
gi|148667828 **Mass:** 45346 **Score:** 45 **Matches:** 1(1) **Sequences:** 1(1)
LanC (bacterial lantibiotic synthetase component C)-like 1, isoform CRA_a [Mus musculus]
gi|148667829 **Mass:** 47990 **Score:** 45 **Matches:** 1(1) **Sequences:** 1(1)
LanC (bacterial lantibiotic synthetase component C)-like 1, isoform CRA_b [Mus musculus]

---

19. gi|12859178 **Mass:** 26301 **Score:** 45 **Matches:** 1(0) **Sequences:** 1(0) **emPAI:** 0.13

unnamed protein product [Mus musculus]

Query	Observed	Mr(expt)	Mr(calc)	Delta	Miss	Score	Expect	Rank	Unique	Peptide
13273	639.2183	1914.6330	1913.8795	0.7534	1	45	0.072	1	U	K.DAEEWFFTKTEELNR.E

Proteins matching the same set of peptides:

gi\|38503465 **Mass:** 50146 **Score:** 45 **Matches:** 1(0) **Sequences:** 1(0)
keratin 17n [Mus musculus]

gi\|85726518 **Mass:** 49857 **Score:** 45 **Matches:** 1(0) **Sequences:** 1(0)
Krt42 protein [Mus musculus]

gi\|154090941 **Mass:** 50102 **Score:** 45 **Matches:** 1(0) **Sequences:** 1(0)
keratin, type I cytoskeletal 42 [Mus musculus]

gi\|156139032 **Mass:** 50162 **Score:** 45 **Matches:** 1(0) **Sequences:** 1(0)
Keratin 42 [Mus musculus]

---

20. gi\|19353667 **Mass:** 19691 **Score:** 43 **Matches:** 2(1) **Sequences:** 2(1) **emPAI:** 0.17
Pcmtd2 protein, partial [Mus musculus]

Query	Observed	Mr(expt)	Mr(calc)	Delta	Miss	Score	Expect	Rank	Unique	Peptide
10138	489.0460	976.0774	975.4662	0.6112	0	43	0.045	1	U	R.TGPSAWETK.K
11516	556.1887	1110.3629	1110.5631	-0.2002	0	43	0.1	1	U	R.METIVFLDK.E

Proteins matching the same set of peptides:

gi\|23956398 **Mass:** 40730 **Score:** 43 **Matches:** 2(1) **Sequences:** 2(1)
protein-L-isoaspartate O-methyltransferase domain-containing protein 2 [Mus musculus]

gi\|26348291 **Mass:** 21087 **Score:** 43 **Matches:** 2(1) **Sequences:** 2(1)
unnamed protein product [Mus musculus]

Mascot: http://www.matrixscience.com/

Mascot Search Results

D17

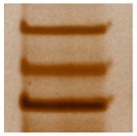

A

```
User                   :
Email                  :
Search title           :
MS data file           : ZIEN-29Nov2017_flyA.mgf
Database               : NCBInr 20120419 (17893860 sequences; 6141683785 residues)
Taxonomy               : Drosophila (fruit flies) (222527 sequences)
Timestamp              : 30 Nov 2017 at 18:31:47 GMT
Enzyme                 : Trypsin
Variable modifications : Carbamidomethyl (C),Oxidation (M)
Mass values            : Monoisotopic
Protein Mass           : Unrestricted
Peptide Mass Tolerance : ± 1.25 Da
Fragment Mass Tolerance: ± 1.001 Da
Max Missed Cleavages   : 1
Instrument type        : ESI-TRAP
Number of queries      : 16095
Protein hits           : gi|17737967  heat shock protein cognate 4, isoform A [Drosophila melanogaster]
                         gi|157658    heat shock protein cognate 72 [Drosophila melanogaster]
                         gi|157667    heat shock protein cognate 71 [Drosophila melanogaster]
                         gi|157678    heat shock cognate 70 protein (partial) (at locus 88E), partial [Drosophila melanogaster]
                         gi|6118254   DEAD-box protein abstrakt [Drosophila melanogaster]
                         gi|195449894 GK22765 [Drosophila willistoni]
                         gi|24651407  prolyl-4-hydroxylase-alpha EFB [Drosophila melanogaster]
                         gi|195341536 GM12882 [Drosophila sechellia]
                         gi|24644396  CG2082, isoform C [Drosophila melanogaster]
                         gi|157891    myosin heavy chain [Drosophila melanogaster]
                         gi|195161163 GL25329 [Drosophila persimilis]
                         gi|194864176 GG23181 [Drosophila erecta]
                         gi|194769542 GF19055 [Drosophila ananassae]
                         gi|194766886 GF22554 [Drosophila ananassae]
```

Select Summary Report

Format As | Select Summary (protein hits) ▼ | Help

Significance threshold p< 0.05 | Max. number of hits AUTO

Standard scoring ○ MudPIT scoring ● | Ions score or expect cut-off 44 | Show sub-sets 0

Show pop-ups ● Suppress pop-ups ○ | Require bold red ☐

Re-Search ◉ All queries ○ Unassigned ○ Below homology threshold ○ Below identity threshold

1. gi|17737967 **Mass:** 71087 **Score:** 168 **Matches:** 7(7) **Sequences:** 6(6) **emPAI:** 0.31
heat shock protein cognate 4, isoform A [Drosophila melanogaster]

Query	Observed	Mr(expt)	Mr(calc)	Delta	Miss	Score	Expect	Rank	Unique	Peptide
12119	587.4327	1172.8509	1172.5826	0.2684	0	51	0.011	1	U	K.WLDANQLADK.E
12465	600.5588	1199.1031	1198.6670	0.4362	0	57	0.0044	1	U	K.DAGTIAGLNVLR.I 12466
12751	613.9276	1225.8405	1225.5285	0.3120	0	49	0.032	1		K.FDDAAVQSDMK.H
13970	678.0540	1354.0934	1353.6235	0.4699	1	53	0.011	1		R.KFDDAAVQSDMK.H
15110	727.5170	1453.0195	1451.8096	1.2099	0	76	3e-005	1	U	K.SVIHDIVLVGGSTR.I
15964	839.5435	1677.0724	1676.8192	0.2532	0	92	1.2e-006	1	U	K.NQVAMNPTQTIFDAK.R

Proteins matching the same set of peptides:

gi|19527633 **Mass:** 71060 **Score:** 168 **Matches:** 7(7) **Sequences:** 6(6)
RH04426p [Drosophila melanogaster]

gi|194900946 **Mass:** 71001 **Score:** 168 **Matches:** 7(7) **Sequences:** 6(6)
GG16900 [Drosophila erecta]

gi|195328813 **Mass:** 70971 **Score:** 168 **Matches:** 7(7) **Sequences:** 6(6)
GM24208 [Drosophila sechellia]

gi|195570732 **Mass:** 70916 **Score:** 168 **Matches:** 7(7) **Sequences:** 6(6)
Hsc70-4 [Drosophila simulans]

---

2. gi|157658 **Mass:** 72190 **Score:** 108 **Matches:** 5(5) **Sequences:** 5(5) **emPAI:** 0.25
heat shock protein cognate 72 [Drosophila melanogaster]

Query	Observed	Mr(expt)	Mr(calc)	Delta	Miss	Score	Expect	Rank	Unique	Peptide
9446	478.0703	954.1261	953.5043	0.6218	0	62	0.00066	1	U	R.ALSGSHQVR.I
11066	552.6559	1103.2972	1103.5710	-0.2738	0	56	0.0035	1	U	K.LESAIDESIK.W
13643	659.0761	1316.1375	1315.6295	0.5080	0	55	0.006	1	U	R.NELESYAYSLK.N
15556	754.5173	1507.0201	1505.7800	1.2402	0	51	0.015	1	U	K.VFAPEEISAMVLGK.M
15667	764.1249	1526.2352	1525.7413	0.4939	0	48	0.028	1	U	R.ITPSYVAFTADGER.L

Proteins matching the same set of peptides:

gi|24641402 **Mass:** 72216 **Score:** 108 **Matches:** 5(5) **Sequences:** 5(5)
heat shock protein cognate 3, isoform A [Drosophila melanogaster]

gi|28557577 **Mass:** 72069 **Score:** 108 **Matches:** 5(5) **Sequences:** 5(5)
RH21402p [Drosophila melanogaster]

gi|125981509 **Mass:** 72261 **Score:** 108 **Matches:** 5(5) **Sequences:** 5(5)
GA17988 [Drosophila pseudoobscura pseudoobscura]

gi|194762782 **Mass:** 72278 **Score:** 108 **Matches:** 5(5) **Sequences:** 5(5)
GF20248 [Drosophila ananassae]
gi|195165202 **Mass:** 72272 **Score:** 108 **Matches:** 5(5) **Sequences:** 5(5)
GL20354 [Drosophila persimilis]
gi|195457222 **Mass:** 72381 **Score:** 108 **Matches:** 5(5) **Sequences:** 5(5)
GK18328 [Drosophila willistoni]
gi|195480743 **Mass:** 72226 **Score:** 108 **Matches:** 5(5) **Sequences:** 5(5)
Hsc70-3 [Drosophila yakuba]

---

3. gi|157667 **Mass:** 74162 **Score:** 100 **Matches:** 6(5) **Sequences:** 5(4) **emPAI:** 0.24
heat shock protein cognate 71 [Drosophila melanogaster]

Query	Observed	Mr(expt)	Mr(calc)	Delta	Miss	Score	Expect	Rank	Unique	Peptide
9395	953.4493	952.4420	952.4865	-0.0445	0	51	0.013	1	U	K.DITNLSYK.V
9494	958.5143	957.5070	957.4879	0.0191	0	45	0.064	1	U	K.VIENAEGAR.T
11337	563.4215	1124.8284	1124.5349	0.2935	1	49	0.015	1	U	K.KAEEYATADK.Q
12759	614.1332	1226.2518	1225.6779	0.5739	0	66	0.00049	1	U	K.DAGQIAGLNVLR.V 12742
13805	668.6088	1335.2030	1334.6718	0.5312	0	45	0.035	1	U	K.ETAEAYLNTPVK.N

Proteins matching the same set of peptides:
gi|24653595 **Mass:** 74020 **Score:** 100 **Matches:** 6(5) **Sequences:** 5(4)
heat shock protein cognate 5 [Drosophila melanogaster]
gi|194883130 **Mass:** 74029 **Score:** 100 **Matches:** 6(5) **Sequences:** 5(4)
GG22433 [Drosophila erecta]
gi|195334294 **Mass:** 74060 **Score:** 100 **Matches:** 6(5) **Sequences:** 5(4)
GM20220 [Drosophila sechellia]
gi|195485915 **Mass:** 74021 **Score:** 100 **Matches:** 6(5) **Sequences:** 5(4)
GE12323 [Drosophila yakuba]
gi|195583328 **Mass:** 74074 **Score:** 100 **Matches:** 6(5) **Sequences:** 5(4)
GD25692 [Drosophila simulans]
gi|194756900 **Mass:** 73926 **Score:** 100 **Matches:** 6(5) **Sequences:** 5(4)
GF11360 [Drosophila ananassae]

---

4. gi|157678 **Mass:** 11411 **Score:** 97 **Matches:** 3(3) **Sequences:** 3(3) **emPAI:** 1.20
heat shock cognate 70 protein (partial) (at locus 88E), partial [Drosophila melanogaster]

Query	Observed	Mr(expt)	Mr(calc)	Delta	Miss	Score	Expect	Rank	Unique	Peptide
12751	613.9276	1225.8405	1225.5285	0.3120	0	49	0.032	1		K.FDDAAVQSDMK.H
13970	678.0540	1354.0934	1353.6235	0.4699	1	53	0.011	1		R.KFDDAAVQSDMK.H
15964	839.5435	1677.0724	1677.8032	-0.7308	0	79	2.5e-005	2	U	K.NEVAMNPTQTIFDAK.R

---

5. gi|6118254 **Mass:** 68888 **Score:** 60 **Matches:** 1(1) **Sequences:** 1(1) **emPAI:** 0.05
DEAD-box protein abstrakt [Drosophila melanogaster]

Query	Observed	Mr(expt)	Mr(calc)	Delta	Miss	Score	Expect	Rank	Unique	Peptide
14629	704.5223	1407.0300	1406.7769	0.2531	0	60	0.002	1	U	R.ILVEGETPSPPIR.S

Proteins matching the same set of peptides:
gi|17977678 **Mass:** 69444 **Score:** 60 **Matches:** 1(1) **Sequences:** 1(1)
abstrakt [Drosophila melanogaster]

---

6. gi|195449894 **Mass:** 21978 **Score:** 57 **Matches:** 1(1) **Sequences:** 1(1) **emPAI:** 0.15
GK22765 [Drosophila willistoni]

Query	Observed	Mr(expt)	Mr(calc)	Delta	Miss	Score	Expect	Rank	Unique	Peptide
9376	475.9925	949.9705	950.4127	-0.4422	0	57	0.0025	1	U	M.TTANACDQK.G

---

7. gi|24651407 **Mass:** 63075 **Score:** 53 **Matches:** 1(1) **Sequences:** 1(1) **emPAI:** 0.05
prolyl-4-hydroxylase-alpha EFB [Drosophila melanogaster]

Query	Observed	Mr(expt)	Mr(calc)	Delta	Miss	Score	Expect	Rank	Unique	Peptide
10856	545.6321	1089.2496	1089.5567	-0.3071	0	53	0.012	1	U	R.AFEGLNLGNR.I

Proteins matching the same set of peptides:
gi|125772807 **Mass:** 62641 **Score:** 53 **Matches:** 1(1) **Sequences:** 1(1)
GA15946 [Drosophila pseudoobscura pseudoobscura]
gi|194765194 **Mass:** 62567 **Score:** 53 **Matches:** 1(1) **Sequences:** 1(1)
GF22904 [Drosophila ananassae]
gi|194905436 **Mass:** 62737 **Score:** 53 **Matches:** 1(1) **Sequences:** 1(1)
GG11753 [Drosophila erecta]
gi|195110919 **Mass:** 55326 **Score:** 53 **Matches:** 1(1) **Sequences:** 1(1)
GI24860 [Drosophila mojavensis]
gi|195159323 **Mass:** 55783 **Score:** 53 **Matches:** 1(1) **Sequences:** 1(1)
GL13463 [Drosophila persimilis]
gi|195391754 **Mass:** 55603 **Score:** 53 **Matches:** 1(1) **Sequences:** 1(1)
GJ24502 [Drosophila virilis]
gi|195452726 **Mass:** 62836 **Score:** 53 **Matches:** 1(1) **Sequences:** 1(1)
GK14136 [Drosophila willistoni]
gi|195505190 **Mass:** 55776 **Score:** 53 **Matches:** 1(1) **Sequences:** 1(1)
GE10881 [Drosophila yakuba]
gi|195575089 **Mass:** 62991 **Score:** 53 **Matches:** 1(1) **Sequences:** 1(1)
GD21521 [Drosophila simulans]

8. gi|195341536 **Mass:** 63009 **Score:** 53 **Matches:** 1(1) **Sequences:** 1(1) **emPAI:** 0.05
GM12882 [Drosophila sechellia]

Query	Observed	Mr(expt)	Mr(calc)	Delta	Miss	Score	Expect	Rank	Unique	Peptide
10856	545.6321	1089.2496	1089.5567	-0.3071	0	53	0.012	1	U	R.AFEGINLGNR.I

9. gi|24644396 **Mass:** 39679 **Score:** 48 **Matches:** 1(1) **Sequences:** 1(1) **emPAI:** 0.08
CG2082, isoform C [Drosophila melanogaster]

Query	Observed	Mr(expt)	Mr(calc)	Delta	Miss	Score	Expect	Rank	Unique	Peptide
12547	**604.5787**	**1810.7142**	**1810.8558**	**-0.1416**	**1**	**48**	**0.036**	**1**	**U**	**R.GRAFSTASSGSLEGANGSR.R**

Proteins matching the same set of peptides:

gi|24644398 **Mass:** 39652 **Score:** 48 **Matches:** 1(1) **Sequences:** 1(1)
CG2082, isoform A [Drosophila melanogaster]
gi|62484286 **Mass:** 40038 **Score:** 48 **Matches:** 1(1) **Sequences:** 1(1)
CG2082, isoform B [Drosophila melanogaster]
gi|194745478 **Mass:** 40170 **Score:** 48 **Matches:** 1(1) **Sequences:** 1(1)
GF18647 [Drosophila ananassae]
gi|194898877 **Mass:** 39987 **Score:** 48 **Matches:** 1(1) **Sequences:** 1(1)
GG10854 [Drosophila erecta]
gi|195343799 **Mass:** 41940 **Score:** 48 **Matches:** 1(1) **Sequences:** 1(1)
GM10591 [Drosophila sechellia]
gi|262331602 **Mass:** 19636 **Score:** 48 **Matches:** 1(1) **Sequences:** 1(1)
AT13994p [Drosophila melanogaster]
gi|281360117 **Mass:** 40012 **Score:** 48 **Matches:** 1(1) **Sequences:** 1(1)
CG2082, isoform G [Drosophila melanogaster]
gi|281360119 **Mass:** 39979 **Score:** 48 **Matches:** 1(1) **Sequences:** 1(1)
CG2082, isoform H [Drosophila melanogaster]
gi|327180770 **Mass:** 41129 **Score:** 48 **Matches:** 1(1) **Sequences:** 1(1)
RH63159p [Drosophila melanogaster]
gi|383292515 **Mass:** 36950 **Score:** 48 **Matches:** 1(1) **Sequences:** 1(1)
CG2082, isoform O [Drosophila melanogaster]

10. gi|157891 **Score:** 48 **Matches:** 1(1) **Sequences:** 1(1) **emPAI:** 0.01
myosin heavy chain [Drosophila melanogaster]

Query	Observed	Mr(expt)	Mr(calc)	Delta	Miss	Score	Expect	Rank	Unique	Peptide
9376	475.9925	949.9705	950.4127	-0.4422	0	48	0.019	2	U	K.MQGETNQK.T

Proteins matching the same set of peptides:

gi|157892	Score: 48	Matches: 1(1)	Sequences: 1(1)
gi|2546936	Score: 48	Matches: 1(1)	Sequences: 1(1)
gi|2546937	Score: 48	Matches: 1(1)	Sequences: 1(1)
gi|2546938	Score: 48	Matches: 1(1)	Sequences: 1(1)
gi|2546939	Score: 48	Matches: 1(1)	Sequences: 1(1)
gi|24584692	Score: 48	Matches: 1(1)	Sequences: 1(1)
gi|24584694	Score: 48	Matches: 1(1)	Sequences: 1(1)
gi|24584696	Score: 48	Matches: 1(1)	Sequences: 1(1)
gi|24584700	Score: 48	Matches: 1(1)	Sequences: 1(1)
gi|24584702	Score: 48	Matches: 1(1)	Sequences: 1(1)
gi|24584704	Score: 48	Matches: 1(1)	Sequences: 1(1)
gi|24584706	Score: 48	Matches: 1(1)	Sequences: 1(1)
gi|24584710	Score: 48	Matches: 1(1)	Sequences: 1(1)
gi|24584712	Score: 48	Matches: 1(1)	Sequences: 1(1)
gi|24584714	Score: 48	Matches: 1(1)	Sequences: 1(1)
gi|24584716	Score: 48	Matches: 1(1)	Sequences: 1(1)
gi|28574239	Score: 48	Matches: 1(1)	Sequences: 1(1)
gi|110825729	Score: 48	Matches: 1(1)	Sequences: 1(1)
gi|194884434	Score: 48	Matches: 1(1)	Sequences: 1(1)
gi|195344578	Score: 48	Matches: 1(1)	Sequences: 1(1)
gi|195436762	Score: 48	Matches: 1(1)	Sequences: 1(1)
gi|195483992	Score: 48	Matches: 1(1)	Sequences: 1(1)
gi|198474044	Score: 48	Matches: 1(1)	Sequences: 1(1)
gi|219990777	Score: 48	Matches: 1(1)	Sequences: 1(1)
gi|281365095	Score: 48	Matches: 1(1)	Sequences: 1(1)
gi|281365099	Score: 48	Matches: 1(1)	Sequences: 1(1)
gi|383291520	Score: 48	Matches: 1(1)	Sequences: 1(1)
gi|383291521	Score: 48	Matches: 1(1)	Sequences: 1(1)
gi|383291522	Score: 48	Matches: 1(1)	Sequences: 1(1)
gi|383291523	Score: 48	Matches: 1(1)	Sequences: 1(1)
gi|383291524	Score: 48	Matches: 1(1)	Sequences: 1(1)

---

11. gi|195161163 **Mass:** 58891 **Score:** 47 **Matches:** 1(1) **Sequences:** 1(1) **emPAI:** 0.06

GL25329 [Drosophila persimilis]

Query	Observed	Mr(expt)	Mr(calc)	Delta	Miss	Score	Expect	Rank	Unique	Peptide
12491	**601.5865**	**1201.1585**	**1200.5232**	**0.6353**	**0**	**47**	**0.05**	**1**	**U**	**R.QDNGGGGGVGGGGGR.F**

---

12. gi|194864176 **Score:** 46 **Matches:** 1(1) **Sequences:** 1(1) **emPAI:** 0.02

GG23181 [Drosophila erecta]

Query	Observed	Mr(expt)	Mr(calc)	Delta	Miss	Score	Expect	Rank	Unique	Peptide
12466	600.5885	1199.1624	1198.6670	0.4955	0	46	0.058	2	U	R.ETNNVIAIGLR.D

13. gi|194769542 **Mass:** 194372 **Score:** 46 **Matches:** 1(1) **Sequences:** 1(1) **emPAI:** 0.02
GF19055 [Drosophila ananassae]

Query	Observed	Mr(expt)	Mr(calc)	Delta	Miss	Score	Expect	Rank	Unique	Peptide
9300	472.3273	942.6400	942.5498	0.0902	0	46	0.046	1	U	K.VIEISVQR.G

Proteins matching the same set of peptides:

gi|194892726 **Mass:** 191336 **Score:** 46 **Matches:** 1(1) **Sequences:** 1(1)
GG19193 [Drosophila erecta]

gi|195132717 **Mass:** 70104 **Score:** 46 **Matches:** 1(1) **Sequences:** 1(1)
GI21732 [Drosophila mojavensis]

gi|195392652 **Mass:** 186045 **Score:** 46 **Matches:** 1(1) **Sequences:** 1(1)
GJ19114 [Drosophila virilis]

gi|195432565 **Mass:** 215970 **Score:** 46 **Matches:** 1(1) **Sequences:** 1(1)
GK19785 [Drosophila willistoni]

gi|195481506 **Mass:** 198954 **Score:** 46 **Matches:** 1(1) **Sequences:** 1(1)
GE17755 [Drosophila yakuba]

gi|198469723 **Mass:** 196607 **Score:** 46 **Matches:** 1(1) **Sequences:** 1(1)
GA23340 [Drosophila pseudoobscura pseudoobscura]

14. gi|194766886 **Score:** 45 **Matches:** 1(1) **Sequences:** 1(1) **emPAI:** 0.01
GF22554 [Drosophila ananassae]

Query	Observed	Mr(expt)	Mr(calc)	Delta	Miss	Score	Expect	Rank	Unique	Peptide
9376	475.9925	949.9705	950.4742	-0.5037	1	45	0.034	3	U	K.MKSAELEK.E

Mascot: http://www.matrixscience.com/

MATRIX SCIENCE Mascot Search Results

D17

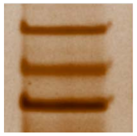

B

User	:	
Email	:	
Search title	:	
MS data file	:	ZIEN-29Nov2017_flyB.mgf
Database	:	NCBInr 20120419 (17893860 sequences; 6141683785 residues)
Taxonomy	:	Drosophila (fruit flies) (222527 sequences)
Timestamp	:	30 Nov 2017 at 18:51:10 GMT
Enzyme	:	Trypsin
Variable modifications	:	Carbamidomethyl (C),Oxidation (M)
Mass values	:	Monoisotopic
Protein Mass	:	Unrestricted
Peptide Mass Tolerance	:	± 1.25 Da
Fragment Mass Tolerance	:	± 1.001 Da
Max Missed Cleavages	:	1
Instrument type	:	Default
Number of queries	:	15798
Protein hits	:	gi|17647799 protein disulfide isomerase, isoform A [Drosophila melanogaster]
		gi|24664683 CG16979 [Drosophila melanogaster]
		gi|195451942 GK13309 [Drosophila willistoni]
		gi|24644396 CG2082, isoform C [Drosophila melanogaster]
		gi|198476294 GA25398 [Drosophila pseudoobscura pseudoobscura]
		gi|24640588 CG1440, isoform A [Drosophila melanogaster]
		gi|24651407 prolyl-4-hydroxylase-alpha EFB [Drosophila melanogaster]
		gi|195161605 GL26392 [Drosophila persimilis]
		gi|195450712 GK13603 [Drosophila willistoni]

Select Summary Report

Format As | Select Summary (protein hits) ▼ | Help

Significance threshold p< 0.05 | Max. number of hits AUTO

Standard scoring ○ MudPIT scoring ◉ | Ions score or expect cut-off 44 | Show sub-sets 0

Show pop-ups ◉ Suppress pop-ups ○ | Require bold red ☐

Re-Search | ◉ All queries ○ Unassigned ○ Below homology threshold ○ Below identity threshold

1. gi|17647799 **Mass:** 55746 **Score:** 120 **Matches:** 3(3) **Sequences:** 3(3) **emPAI:** 0.19

protein disulfide isomerase, isoform A [Drosophila melanogaster]

Query Observed Mr(expt) Mr(calc) Delta Miss Score Expect Rank Unique Peptide

12211	608.3029	1214.5912	1214.6659	-0.0747	0	55	0.0097	1	U	R.QAADIIAWVTK.K
13260	669.5514	1337.0882	1336.6180	0.4702	0	86	2.5e-006	1	U	K.MDSTANELESIK.I
15162	754.9669	1507.9192	1506.7202	1.1990	0	62	0.00091	1	U	K.SVFEGELNEENLK.K

Proteins matching the same set of peptides:
gi|195327719 **Mass:** 55772 **Score:** 120 **Matches:** 3(3) **Sequences:** 3(3)
GM24501 [Drosophila sechellia]

---

2. gi|24664683 **Mass:** 68207 **Score:** 66 **Matches:** 1(1) **Sequences:** 1(1) **emPAI:** 0.05
CG16979 [Drosophila melanogaster]

Query	Observed	Mr(expt)	Mr(calc)	Delta	Miss	Score	Expect	Rank	Unique	Peptide
15088	751.1436	1500.2727	1500.7937	-0.5210	0	66	0.00048	1	U	R.VADGNLVFNVPQTK.I

Proteins matching the same set of peptides:
gi|194872892 **Mass:** 68503 **Score:** 66 **Matches:** 1(1) **Sequences:** 1(1)
GG15911 [Drosophila erecta]
gi|195327809 **Mass:** 68273 **Score:** 66 **Matches:** 1(1) **Sequences:** 1(1)
GM25541 [Drosophila sechellia]
gi|195477958 **Mass:** 68443 **Score:** 66 **Matches:** 1(1) **Sequences:** 1(1)
GE23132 [Drosophila yakuba]
gi|195590489 **Mass:** 68190 **Score:** 66 **Matches:** 1(1) **Sequences:** 1(1)
GD14556 [Drosophila simulans]

---

3. gi|195451942 **Mass:** 253843 **Score:** 53 **Matches:** 1(1) **Sequences:** 1(1) **emPAI:** 0.01
GK13309 [Drosophila willistoni]

Query	Observed	Mr(expt)	Mr(calc)	Delta	Miss	Score	Expect	Rank	Unique	Peptide
15598	801.8900	1601.7655	1600.7917	0.9737	0	53	0.012	1	U	R.NGGLGAAGIVASNSGSNR.V

---

4. gi|24644396 **Mass:** 39679 **Score:** 52 **Matches:** 1(1) **Sequences:** 1(1) **emPAI:** 0.08
CG2082, isoform C [Drosophila melanogaster]

Query	Observed	Mr(expt)	Mr(calc)	Delta	Miss	Score	Expect	Rank	Unique	Peptide
12135	604.6827	1811.0264	1810.8558	0.1706	1	52	0.016	1	U	R.GRAFSTASSGSLEGANGSR.R

Proteins matching the same set of peptides:
gi|24644398 **Mass:** 39652 **Score:** 52 **Matches:** 1(1) **Sequences:** 1(1)
CG2082, isoform A [Drosophila melanogaster]
gi|62484286 **Mass:** 40038 **Score:** 52 **Matches:** 1(1) **Sequences:** 1(1)

CG2082, isoform B [Drosophila melanogaster]

gi|194745478 **Mass:** 40170 **Score:** 52 **Matches:** 1(1) **Sequences:** 1(1)
GF18647 [Drosophila ananassae]

gi|194898877 **Mass:** 39987 **Score:** 52 **Matches:** 1(1) **Sequences:** 1(1)
GG10854 [Drosophila erecta]

gi|195343799 **Mass:** 41940 **Score:** 52 **Matches:** 1(1) **Sequences:** 1(1)
GM10591 [Drosophila sechellia]

gi|262331602 **Mass:** 19636 **Score:** 52 **Matches:** 1(1) **Sequences:** 1(1)
AT13994p [Drosophila melanogaster]

gi|281360117 **Mass:** 40012 **Score:** 52 **Matches:** 1(1) **Sequences:** 1(1)
CG2082, isoform G [Drosophila melanogaster]

gi|281360119 **Mass:** 39979 **Score:** 52 **Matches:** 1(1) **Sequences:** 1(1)
CG2082, isoform H [Drosophila melanogaster]

gi|327180770 **Mass:** 41129 **Score:** 52 **Matches:** 1(1) **Sequences:** 1(1)
RH63159p [Drosophila melanogaster]

gi|383292515 **Mass:** 36950 **Score:** 52 **Matches:** 1(1) **Sequences:** 1(1)
CG2082, isoform O [Drosophila melanogaster]

---

5. gi|198476294 **Mass:** 41611 **Score:** 49 **Matches:** 1(1) **Sequences:** 1(1) **emPAI:** 0.08
GA25398 [Drosophila pseudoobscura pseudoobscura]

Query	Observed	Mr(expt)	Mr(calc)	Delta	Miss	Score	Expect	Rank	Unique	Peptide
14689	729.6755	1457.3365	1456.7885	0.5480	1	49	0.025	1	U	R.ETQIKNNIEQIK.D

---

6. gi|24640588 **Mass:** 55121 **Score:** 49 **Matches:** 1(1) **Sequences:** 1(1) **emPAI:** 0.06
CG1440, isoform A [Drosophila melanogaster]

Query	Observed	Mr(expt)	Mr(calc)	Delta	Miss	Score	Expect	Rank	Unique	Peptide
15015	746.5173	2236.5302	2236.8925	-0.3624	0	49	0.026	1	U	M.SDNNSGSGGGGSGGGGAGNNNSANSGK.N

---

7. gi|24651407 **Mass:** 63075 **Score:** 47 **Matches:** 2(2) **Sequences:** 2(2) **emPAI:** 0.11
prolyl-4-hydroxylase-alpha EFB [Drosophila melanogaster]

Query	Observed	Mr(expt)	Mr(calc)	Delta	Miss	Score	Expect	Rank	Unique	Peptide
10488	541.1962	1080.3779	1079.4077	0.9702	0	45	0.045	1	U	K.GDDGTDEMPK.S
15695	844.5203	1687.0260	1686.7308	0.2952	0	47	0.045	1	U	R.RPCDLEEDHGEFAI.-

Proteins matching the same set of peptides:

gi|195505190 **Mass:** 55776 **Score:** 47 **Matches:** 2(2) **Sequences:** 2(2)
GE10881 [Drosophila yakuba]

---

8. gi|195161605 **Mass:** 46663 **Score:** 47 **Matches:** 1(1) **Sequences:** 1(1) **emPAI:** 0.07
GL26392 [Drosophila persimilis]

Query	Observed	Mr(expt)	Mr(calc)	Delta	Miss	Score	Expect	Rank	Unique	Peptide
14284	712.2616	1422.5086	1422.7428	-0.2342	1	47	0.042	1	U	K.LIESMEKFLEGK.L

Proteins matching the same set of peptides:
gi|198472755 **Mass:** 46598 **Score:** 47 **Matches:** 1(1) **Sequences:** 1(1)
GA14329 [Drosophila pseudoobscura pseudoobscura]

---

9. gi|195450712 **Mass:** 342814 **Score:** 45 **Matches:** 1(0) **Sequences:** 1(0) **emPAI:** 0.01
GK13603 [Drosophila willistoni]

Query	Observed	Mr(expt)	Mr(calc)	Delta	Miss	Score	Expect	Rank	Unique	Peptide
15099	751.6011	1501.1876	1501.7922	-0.6046	1	45	0.057	1	U	K.SCELLDIQQRIK.S

Mascot: http://www.matrixscience.com/

Mascot Search Results

D17

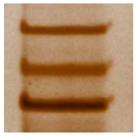

C

```
User                    :
Email                   :
Search title            :
MS data file            : ZIEN-29Nov2017_flyC.mgf
Database                : NCBInr 20120419 (17893860 sequences; 6141683785 residues)
Taxonomy                : Drosophila (fruit flies) (222527 sequences)
Timestamp               : 30 Nov 2017 at 19:12:36 GMT
Enzyme                  : Trypsin
Variable modifications  : Carbamidomethyl (C),Oxidation (M)
Mass values             : Monoisotopic
Protein Mass            : Unrestricted
Peptide Mass Tolerance  : ± 1.25 Da
Fragment Mass Tolerance: ± 1.001 Da
Max Missed Cleavages    : 1
Instrument type         : Default
Number of queries       : 16295
Protein hits            : gi|195590397 GD12572 [Drosophila simulans]
                          gi|17981717  catalase [Drosophila melanogaster]
                          gi|195022939 GH14374 [Drosophila grimshawi]
                          gi|8444      unnamed protein product [Drosophila melanogaster]
                          gi|287945    ATP synthase beta subunit [Drosophila melanogaster]
                          gi|19921848  CG8258 [Drosophila melanogaster]
                          gi|24655737  beta-Tubulin at 56D, isoform B [Drosophila melanogaster]
                          gi|24651407  prolyl-4-hydroxylase-alpha EFB [Drosophila melanogaster]
                          gi|195385695 GJ11603 [Drosophila virilis]
                          gi|195438453 GK24169 [Drosophila willistoni]
                          gi|3108349   pyruvate kinase [Drosophila melanogaster]
                          gi|195434210 GK19056 [Drosophila willistoni]
                          gi|21356731  CG5112 [Drosophila melanogaster]
                          gi|195395688 GJ10965 [Drosophila virilis]
                          gi|195434441 GK14789 [Drosophila willistoni]
                          gi|194763631 GF20995 [Drosophila ananassae]
                          gi|194901836 GG18651 [Drosophila erecta]
                          gi|24648478  KaiRIA [Drosophila melanogaster]
                          gi|195023900 GH20904 [Drosophila grimshawi]
                          gi|195488195 GE14061 [Drosophila yakuba]
                          gi|195126431 GI13073 [Drosophila mojavensis]
                          gi|16554779  no on or off transient A [Drosophila virilis]
```

Select Summary Report

Format As | Select Summary (protein hits) ▼ | Help

Significance threshold p< 0.05 Max. number of hits AUTO

Standard scoring ○ MudPIT scoring ◉ Ions score or expect cut-off 44 Show sub-sets 0

Show pop-ups ◉ Suppress pop-ups ○ Require bold red ☐

Re-Search ◉ All queries ○ Unassigned ○ Below homology threshold ○ Below identity threshold

1. gi|195590397 **Mass:** 55745 **Score:** 238 **Matches:** 12(12) **Sequences:** 8(8) **emPAI:** 0.58

GD12572 [Drosophila simulans]

Query	Observed	Mr(expt)	Mr(calc)	Delta	Miss	Score	Expect	Rank	Unique	Peptide
12656	608.6249	1215.2352	1214.6659	0.5693	0	58	0.0033	1	U	R.QAADIIAWVTK.K 12647
13773	669.4131	1336.8116	1336.6180	0.1936	0	68	0.00042	1	U	K.MDSTANELESIK.I
14382	694.6771	1387.3397	1387.7347	-0.3950	0	56	0.0062	1	U	K.QLAPIYDQLAEK.Y 14377
14981	719.0861	1436.1577	1435.7195	0.4382	0	46	0.046	1	U	K.DLTSVADAEQFLK.D
15648	754.4683	1506.9221	1506.7930	0.1292	0	57	0.0027	1	U	K.VLVSSNFESVALDK.S
15743	760.4873	1518.9600	1518.7930	0.1671	0	54	0.0087	1	U	K.VEEGVLVATVDNFK.Q 15736 15740
16281	1115.7028	2229.3910	2229.1430	0.2481	0	72	8.8e-005	1	U	K.FAQVQSLPLIVDFNHESASK.I
16021	787.0250	2358.0532	2357.2379	0.8153	1	58	0.0036	1	U	K.KFAQVQSLPLIVDFNHESASK.I

Proteins matching the same set of peptides:

gi|17647799 **Mass:** 55746 **Score:** 238 **Matches:** 12(12) **Sequences:** 8(8)
protein disulfide isomerase, isoform A [Drosophila melanogaster]

gi|194751557 **Mass:** 55553 **Score:** 238 **Matches:** 12(12) **Sequences:** 8(8)
GF10739 [Drosophila ananassae]

gi|194871212 **Mass:** 55744 **Score:** 238 **Matches:** 12(12) **Sequences:** 8(8)
GG13681 [Drosophila erecta]

gi|195327719 **Mass:** 55772 **Score:** 238 **Matches:** 12(12) **Sequences:** 8(8)
GM24501 [Drosophila sechellia]

---

2. gi|17981717 **Mass:** 57113 **Score:** 88 **Matches:** 3(3) **Sequences:** 3(3) **emPAI:** 0.18

catalase [Drosophila melanogaster]

Query	Observed	Mr(expt)	Mr(calc)	Delta	Miss	Score	Expect	Rank	Unique	Peptide
12231	589.4701	1176.9256	1176.6060	0.3196	0	46	0.048	1	U	R.MLTEELNLAK.S
12891	619.3951	1236.7757	1236.5986	0.1771	0	45	0.051	1	U	R.DAASNQLIDYK.N
15036	721.9436	1441.8726	1440.6481	1.2245	0	83	6.4e-006	1		R.FSTVGGESGSADTAR.D

Proteins matching the same set of peptides:

gi|194871234 **Mass:** 57183 **Score:** 88 **Matches:** 3(3) **Sequences:** 3(3)

GG13679 [Drosophila erecta]

gi|195352234 **Mass:** 57134 **Score:** 88 **Matches:** 3(3) **Sequences:** 3(3)
Cat [Drosophila sechellia]

gi|195494497 **Mass:** 57064 **Score:** 88 **Matches:** 3(3) **Sequences:** 3(3)
GE19975 [Drosophila yakuba]

gi|195591356 **Mass:** 57134 **Score:** 88 **Matches:** 3(3) **Sequences:** 3(3)
catalase [Drosophila simulans]

---

3. gi|195022939 **Mass:** 57059 **Score:** 86 **Matches:** 2(2) **Sequences:** 2(2) **emPAI:** 0.12
GH14374 [Drosophila grimshawi]

Query	Observed	Mr(expt)	Mr(calc)	Delta	Miss	Score	Expect	Rank	Unique	Peptide
12891	619.3951	1236.7757	1236.5986	0.1771	0	45	0.051	1	U	R.DAASNQLLDYK.N
15036	721.9436	1441.8726	1440.6481	1.2245	0	83	6.4e-006	1		R.FSTVGGESGSADTAR.D

Proteins matching the same set of peptides:

gi|195378516 **Mass:** 57117 **Score:** 86 **Matches:** 2(2) **Sequences:** 2(2)
GJ11574 [Drosophila virilis]

---

4. gi|8444 **Mass:** 62291 **Score:** 84 **Matches:** 2(2) **Sequences:** 2(2) **emPAI:** 0.11
unnamed protein product [Drosophila melanogaster]

Query	Observed	Mr(expt)	Mr(calc)	Delta	Miss	Score	Expect	Rank	Unique	Peptide
12806	**615.2691**	**1228.5236**	**1228.6775**	**-0.1539**	**0**	**64**	**0.0013**	**1**	**U**	**K.SNILVATDVAAR.G**
13278	**640.6392**	**1279.2638**	**1278.7296**	**0.5342**	**0**	**62**	**0.0014**	**1**	**U**	**R.GDGPIALVLAPTR.E**

Proteins matching the same set of peptides:

gi|24644479 **Mass:** 78500 **Score:** 84 **Matches:** 2(2) **Sequences:** 2(2)
Rm62, isoform A [Drosophila melanogaster]

gi|24644481 **Mass:** 62436 **Score:** 84 **Matches:** 2(2) **Sequences:** 2(2)
Rm62, isoform D [Drosophila melanogaster]

gi|24644483 **Mass:** 62791 **Score:** 84 **Matches:** 2(2) **Sequences:** 2(2)
Rm62, isoform E [Drosophila melanogaster]

gi|24644485 **Mass:** 62829 **Score:** 84 **Matches:** 2(2) **Sequences:** 2(2)
Rm62, isoform C [Drosophila melanogaster]

gi|194745414 **Mass:** 77004 **Score:** 84 **Matches:** 2(2) **Sequences:** 2(2)
GF18634 [Drosophila ananassae]

gi|194898941 **Mass:** 78362 **Score:** 84 **Matches:** 2(2) **Sequences:** 2(2)
GG10666 [Drosophila erecta]

gi|195144578 **Mass:** 71625 **Score:** 84 **Matches:** 2(2) **Sequences:** 2(2)
GL23489 [Drosophila persimilis]

gi|195343855 **Mass:** 78805 **Score:** 84 **Matches:** 2(2) **Sequences:** 2(2)
GM10578 [Drosophila sechellia]
gi|195453112 **Mass:** 81548 **Score:** 84 **Matches:** 2(2) **Sequences:** 2(2)
GK14214 [Drosophila willistoni]
gi|195502160 **Mass:** 78418 **Score:** 84 **Matches:** 2(2) **Sequences:** 2(2)
GE24123 [Drosophila yakuba]
gi|198452778 **Mass:** 79875 **Score:** 84 **Matches:** 2(2) **Sequences:** 2(2)
GA10214 [Drosophila pseudoobscura pseudoobscura]
gi|383292518 **Mass:** 50067 **Score:** 84 **Matches:** 2(2) **Sequences:** 2(2)
Rm62, isoform J [Drosophila melanogaster]
gi|383292519 **Mass:** 53163 **Score:** 84 **Matches:** 2(2) **Sequences:** 2(2)
Rm62, isoform K [Drosophila melanogaster]
gi|383292520 **Mass:** 51999 **Score:** 84 **Matches:** 2(2) **Sequences:** 2(2)
Rm62, isoform L [Drosophila melanogaster]
gi|383292521 **Mass:** 52514 **Score:** 84 **Matches:** 2(2) **Sequences:** 2(2)
Rm62, isoform M [Drosophila melanogaster]
gi|383873392 **Mass:** 33568 **Score:** 84 **Matches:** 2(2) **Sequences:** 2(2)
MIP33508p1 [Drosophila melanogaster]
gi|195109284 **Mass:** 79484 **Score:** 84 **Matches:** 2(2) **Sequences:** 2(2)
GI23184 [Drosophila mojavensis]

---

5. gi|287945 **Mass:** 53487 **Score:** 63 **Matches:** 2(2) **Sequences:** 2(2) **emPAI:** 0.13
ATP synthase beta subunit [Drosophila melanogaster]

Query	Observed	Mr(expt)	Mr(calc)	Delta	Miss	Score	Expect	Rank	Unique	Peptide
10435	**517.1084**	**1032.2022**	**1032.5604**	**-0.3582**	**0**	**46**	**0.035**	**1**	**U**	**K.VLDTGYPIR.I**
14074	**684.3671**	**1366.7196**	**1366.7456**	**-0.0260**	**0**	**61**	**0.0019**	**1**	**U**	**R.IINVIGEPIDER.G**

Proteins matching the same set of peptides:
gi|24638766 **Mass:** 54074 **Score:** 63 **Matches:** 2(2) **Sequences:** 2(2)
ATP synthase-beta, isoform A [Drosophila melanogaster]
gi|38048209 **Mass:** 21234 **Score:** 63 **Matches:** 2(2) **Sequences:** 2(2)
similar to Drosophila melanogaster ATPsyn-beta, partial [Drosophila yakuba]
gi|38048553 **Mass:** 21774 **Score:** 63 **Matches:** 2(2) **Sequences:** 2(2)
similar to Drosophila melanogaster ATPsyn-beta, partial [Drosophila yakuba]
gi|194770714 **Mass:** 54254 **Score:** 63 **Matches:** 2(2) **Sequences:** 2(2)
GF21882 [Drosophila ananassae]
gi|195064082 **Mass:** 54196 **Score:** 63 **Matches:** 2(2) **Sequences:** 2(2)
GH23965 [Drosophila grimshawi]

gi|195134016 **Mass:** 54192 **Score:** 63 **Matches:** 2(2) **Sequences:** 2(2)
GI14045 [Drosophila mojavensis]
gi|195355680 **Mass:** 41109 **Score:** 63 **Matches:** 2(2) **Sequences:** 2(2)
GM13022 [Drosophila sechellia]
gi|195450700 **Mass:** 54274 **Score:** 63 **Matches:** 2(2) **Sequences:** 2(2)
GK13685 [Drosophila willistoni]
gi|195469417 **Mass:** 54104 **Score:** 63 **Matches:** 2(2) **Sequences:** 2(2)
ATPsyn-beta [Drosophila yakuba]
gi|195564346 **Mass:** 47247 **Score:** 63 **Matches:** 2(2) **Sequences:** 2(2)
ATPsyn-beta [Drosophila simulans]

---

6. gi|19921848 **Mass:** 59396 **Score:** 62 **Matches:** 2(2) **Sequences:** 2(2) **emPAI:** 0.11
CG8258 [Drosophila melanogaster]

Query	Observed	Mr(expt)	Mr(calc)	Delta	Miss	Score	Expect	Rank	Unique	Peptide
11419	560.9957	1119.9768	1119.5560	0.4208	0	46	0.056	1	U	R.ALDDAINNFK.C
15559	749.0649	1496.1153	1495.7406	0.3747	0	57	0.0039	1	U	R.LGITTAEISDGYEK.A

---

7. gi|24655737 **Mass:** 50115 **Score:** 62 **Matches:** 2(2) **Sequences:** 2(2) **emPAI:** 0.14
beta-Tubulin at 56D, isoform B [Drosophila melanogaster]

Query	Observed	Mr(expt)	Mr(calc)	Delta	Miss	Score	Expect	Rank	Unique	Peptide
13754	668.3984	1334.7823	1334.6904	0.0919	0	53	0.012	1	U	R.IMNTYSVVPSPK.V
16093	801.2383	1600.4620	1600.8131	-0.3511	0	50	0.017	1	U	R.AVLVDLEPGTMDSVR.S

Proteins matching the same set of peptides:
gi|24655741 **Mass:** 51264 **Score:** 62 **Matches:** 2(2) **Sequences:** 2(2)
beta-Tubulin at 56D, isoform A [Drosophila melanogaster]
gi|188529361 **Mass:** 20477 **Score:** 62 **Matches:** 2(2) **Sequences:** 2(2)
beta-tubulin [Drosophila silvestris]
gi|194881355 **Mass:** 53339 **Score:** 62 **Matches:** 2(2) **Sequences:** 2(2)
GG20906 [Drosophila erecta]
gi|306922435 **Mass:** 51322 **Score:** 62 **Matches:** 2(2) **Sequences:** 2(2)
GH12877p [Drosophila melanogaster]

---

8. gi|24651407 **Mass:** 63075 **Score:** 60 **Matches:** 1(1) **Sequences:** 1(1) **emPAI:** 0.05
prolyl-4-hydroxylase-alpha EFB [Drosophila melanogaster]

Query	Observed	Mr(expt)	Mr(calc)	Delta	Miss	Score	Expect	Rank	Unique	Peptide
16080	799.1600	1596.3055	1595.7791	0.5264	0	60	0.0017	1	U	R.LQDTYQLDTSSVAR.G

Proteins matching the same set of peptides:

gi|125772807 **Mass:** 62641 **Score:** 60 **Matches:** 1(1) **Sequences:** 1(1)
GA15946 [Drosophila pseudoobscura pseudoobscura]

gi|194765194 **Mass:** 62567 **Score:** 60 **Matches:** 1(1) **Sequences:** 1(1)
GF22904 [Drosophila ananassae]

gi|194905436 **Mass:** 62737 **Score:** 60 **Matches:** 1(1) **Sequences:** 1(1)
GG11753 [Drosophila erecta]

gi|195055779 **Mass:** 55833 **Score:** 60 **Matches:** 1(1) **Sequences:** 1(1)
GH14110 [Drosophila grimshawi]

gi|195159323 **Mass:** 55783 **Score:** 60 **Matches:** 1(1) **Sequences:** 1(1)
GL13463 [Drosophila persimilis]

gi|195341536 **Mass:** 63009 **Score:** 60 **Matches:** 1(1) **Sequences:** 1(1)
GM12882 [Drosophila sechellia]

gi|195391754 **Mass:** 55603 **Score:** 60 **Matches:** 1(1) **Sequences:** 1(1)
GJ24502 [Drosophila virilis]

gi|195452726 **Mass:** 62836 **Score:** 60 **Matches:** 1(1) **Sequences:** 1(1)
GK14136 [Drosophila willistoni]

gi|195505190 **Mass:** 55776 **Score:** 60 **Matches:** 1(1) **Sequences:** 1(1)
GE10881 [Drosophila yakuba]

gi|195575089 **Mass:** 62991 **Score:** 60 **Matches:** 1(1) **Sequences:** 1(1)
GD21521 [Drosophila simulans]

---

9. gi|195385695 **Mass:** 47177 **Score:** 60 **Matches:** 2(2) **Sequences:** 1(1) **emPAI:** 0.07
GJ11603 [Drosophila virilis]

Query	Observed	Mr(expt)	Mr(calc)	Delta	Miss	Score	Expect	Rank	Unique	Peptide
13981	680.0380	1358.0614	1358.7228	-0.6614	1	54	0.0044	1	U	-.MSKQLADLQPTK.F 13982

---

10. gi|195438453 **Mass:** 56787 **Score:** 52 **Matches:** 1(1) **Sequences:** 1(1) **emPAI:** 0.06
GK24169 [Drosophila willistoni]

Query	Observed	Mr(expt)	Mr(calc)	Delta	Miss	Score	Expect	Rank	Unique	Peptide
12945	622.3690	1242.7234	1241.6364	1.0870	0	52	0.018	1	U	K.NQDVAAVQEIR.E

Proteins matching the same set of peptides:

gi|17137450 **Mass:** 55970 **Score:** 52 **Matches:** 1(1) **Sequences:** 1(1)
regulatory particle non-ATPase 3 [Drosophila melanogaster]

gi|19527921 **Mass:** 55992 **Score:** 52 **Matches:** 1(1) **Sequences:** 1(1)
AT15146p [Drosophila melanogaster]

gi|125986955 **Mass:** 56336 **Score:** 52 **Matches:** 1(1) **Sequences:** 1(1)
GA10344 [Drosophila pseudoobscura pseudoobscura]

gi|170280395 **Mass:** 26416 **Score:** 52 **Matches:** 1(1) **Sequences:** 1(1)
diphenol oxidase A2, partial [Drosophila erecta]
gi|170280397 **Mass:** 26437 **Score:** 52 **Matches:** 1(1) **Sequences:** 1(1)
diphenol oxidase A2, partial [Drosophila mauritiana]
gi|170280399 **Mass:** 26398 **Score:** 52 **Matches:** 1(1) **Sequences:** 1(1)
diphenol oxidase A2, partial [Drosophila melanogaster]
gi|170280401 **Mass:** 26430 **Score:** 52 **Matches:** 1(1) **Sequences:** 1(1)
diphenol oxidase A2, partial [Drosophila santomea]
gi|170280403 **Mass:** 26509 **Score:** 52 **Matches:** 1(1) **Sequences:** 1(1)
diphenol oxidase A2, partial [Drosophila sechellia]
gi|170280405 **Mass:** 26414 **Score:** 52 **Matches:** 1(1) **Sequences:** 1(1)
diphenol oxidase A2, partial [Drosophila simulans]
gi|170280407 **Mass:** 26458 **Score:** 52 **Matches:** 1(1) **Sequences:** 1(1)
diphenol oxidase A2, partial [Drosophila teissieri]
gi|194879789 **Mass:** 56011 **Score:** 52 **Matches:** 1(1) **Sequences:** 1(1)
GG21155 [Drosophila erecta]
gi|195156337 **Mass:** 56318 **Score:** 52 **Matches:** 1(1) **Sequences:** 1(1)
GL26159 [Drosophila persimilis]
gi|195345003 **Mass:** 56051 **Score:** 52 **Matches:** 1(1) **Sequences:** 1(1)
GM17319 [Drosophila sechellia]
gi|195484349 **Mass:** 56025 **Score:** 52 **Matches:** 1(1) **Sequences:** 1(1)
GE13227 [Drosophila yakuba]
gi|195580065 **Mass:** 56028 **Score:** 52 **Matches:** 1(1) **Sequences:** 1(1)
GD24178 [Drosophila simulans]

---

11. gi|3108349 **Mass:** 57437 **Score:** 50 **Matches:** 1(1) **Sequences:** 1(1) **emPAI:** 0.06
pyruvate kinase [Drosophila melanogaster]

Query	Observed	Mr(expt)	Mr(calc)	Delta	Miss	Score	Expect	Rank	Unique	Peptide
11674	571.3002	1140.5859	1140.6026	-0.0167	0	50	0.031	1	U	R.GDLGIEIPAEK.V

Proteins matching the same set of peptides:
gi|24648964 **Mass:** 55024 **Score:** 50 **Matches:** 1(1) **Sequences:** 1(1)
pyruvate kinase, isoform B [Drosophila melanogaster]
gi|27819773 **Mass:** 57438 **Score:** 50 **Matches:** 1(1) **Sequences:** 1(1)
RH07636p [Drosophila melanogaster]
gi|28571814 **Mass:** 57404 **Score:** 50 **Matches:** 1(1) **Sequences:** 1(1)
pyruvate kinase, isoform A [Drosophila melanogaster]
gi|38047755 **Mass:** 19755 **Score:** 50 **Matches:** 1(1) **Sequences:** 1(1)

similar to Drosophila melanogaster PyK, partial [Drosophila yakuba]

gi|194744590 **Mass:** 57203 **Score:** 50 **Matches:** 1(1) **Sequences:** 1(1)
GF18439 [Drosophila ananassae]

gi|194911138 **Mass:** 57502 **Score:** 50 **Matches:** 1(1) **Sequences:** 1(1)
GG11123 [Drosophila erecta]

gi|195053328 **Mass:** 57522 **Score:** 50 **Matches:** 1(1) **Sequences:** 1(1)
GH20329 [Drosophila grimshawi]

gi|195056081 **Mass:** 57549 **Score:** 50 **Matches:** 1(1) **Sequences:** 1(1)
GH13278 [Drosophila grimshawi]

gi|195112292 **Mass:** 57581 **Score:** 50 **Matches:** 1(1) **Sequences:** 1(1)
GI22374 [Drosophila mojavensis]

gi|195145138 **Mass:** 57003 **Score:** 50 **Matches:** 1(1) **Sequences:** 1(1)
GL24201 [Drosophila persimilis]

gi|195330979 **Mass:** 57418 **Score:** 50 **Matches:** 1(1) **Sequences:** 1(1)
GM26420 [Drosophila sechellia]

gi|195391442 **Mass:** 102583 **Score:** 50 **Matches:** 1(1) **Sequences:** 1(1)
GJ24408 [Drosophila virilis]

gi|195453308 **Mass:** 57517 **Score:** 50 **Matches:** 1(1) **Sequences:** 1(1)
GK14262 [Drosophila willistoni]

gi|195502545 **Mass:** 57418 **Score:** 50 **Matches:** 1(1) **Sequences:** 1(1)
PyK [Drosophila yakuba]

gi|198452351 **Mass:** 57033 **Score:** 50 **Matches:** 1(1) **Sequences:** 1(1)
GA26534 [Drosophila pseudoobscura pseudoobscura]

---

12. gi|195434210 **Mass:** 21374 **Score:** 49 **Matches:** 1(1) **Sequences:** 1(1) **emPAI:** 0.16
GK19056 [Drosophila willistoni]

Query	Observed	Mr(expt)	Mr(calc)	Delta	Miss	Score	Expect	Rank	Unique	Peptide
12267	**590.4731**	**1178.9317**	**1178.5866**	**0.3451**	**0**	**49**	**0.013**	**1**	**U**	**K.YQLIQMNNR.S**

---

13. gi|21356731 **Mass:** 58448 **Score:** 49 **Matches:** 1(1) **Sequences:** 1(1) **emPAI:** 0.06
CG5112 [Drosophila melanogaster]

Query	Observed	Mr(expt)	Mr(calc)	Delta	Miss	Score	Expect	Rank	Unique	Peptide
11365	**558.2457**	**1114.4769**	**1113.6142**	**0.8627**	**0**	**49**	**0.022**	**1**	**U**	**K.SAVELAQQIR.E**

Proteins matching the same set of peptides:

gi|125776626 **Mass:** 58393 **Score:** 49 **Matches:** 1(1) **Sequences:** 1(1)
GA18668 [Drosophila pseudoobscura pseudoobscura]

gi|194908530 **Mass:** 58327 **Score:** 49 **Matches:** 1(1) **Sequences:** 1(1)

GG11409 [Drosophila erecta]

gi|195152431 **Mass:** 58361 **Score:** 49 **Matches:** 1(1) **Sequences:** 1(1)
GL22143 [Drosophila persimilis]

gi|195349457 **Mass:** 58431 **Score:** 49 **Matches:** 1(1) **Sequences:** 1(1)
GM10248 [Drosophila sechellia]

gi|195453896 **Mass:** 58368 **Score:** 49 **Matches:** 1(1) **Sequences:** 1(1)
GK12847 [Drosophila willistoni]

gi|195504320 **Mass:** 58393 **Score:** 49 **Matches:** 1(1) **Sequences:** 1(1)
GE23605 [Drosophila yakuba]

gi|195573909 **Mass:** 58441 **Score:** 49 **Matches:** 1(1) **Sequences:** 1(1)
GD21221 [Drosophila simulans]

---

14. gi|195395688 **Mass:** 58173 **Score:** 49 **Matches:** 1(1) **Sequences:** 1(1) **emPAI:** 0.06
GJ10965 [Drosophila virilis]

Query	Observed	Mr(expt)	Mr(calc)	Delta	Miss	Score	Expect	Rank	Unique	Peptide
11365	558.2457	1114.4769	1113.6506	0.8263	1	49	0.022	1	U	K.SAVELAKQIR.E

---

15. gi|195434441 **Mass:** 51967 **Score:** 48 **Matches:** 1(1) **Sequences:** 1(1) **emPAI:** 0.06
GK14789 [Drosophila willistoni]

Query	Observed	Mr(expt)	Mr(calc)	Delta	Miss	Score	Expect	Rank	Unique	Peptide
14049	**683.3903**	**1364.7660**	**1364.6295**	**0.1364**	**0**	**48**	**0.041**	**1**	**U**	**K.MDSHFNAFQLR.N**

---

16. gi|194763631 **Score:** 48 **Matches:** 1(1) **Sequences:** 1(1) **emPAI:** 0.03
GF20995 [Drosophila ananassae]

Query	Observed	Mr(expt)	Mr(calc)	Delta	Miss	Score	Expect	Rank	Unique	Peptide
13278	640.6392	1279.2638	1278.7296	0.5342	0	48	0.036	2	U	R.GEGPVALVLAPTR.E

Proteins matching the same set of peptides:

gi|195438679 **Score:** 48 **Matches:** 1(1) **Sequences:** 1(1)

---

17. gi|194901836 **Mass:** 5994 **Score:** 47 **Matches:** 1(1) **Sequences:** 1(1) **emPAI:** 0.60
GG18651 [Drosophila erecta]

Query	Observed	Mr(expt)	Mr(calc)	Delta	Miss	Score	Expect	Rank	Unique	Peptide
13236	**638.0350**	**1274.0554**	**1273.5760**	**0.4794**	**1**	**47**	**0.046**	**1**	**U**	**K.KGGGGGGGGGGGGSGNK.N**

---

18. gi|24648478 **Mass:** 101593 **Score:** 46 **Matches:** 1(1) **Sequences:** 1(1) **emPAI:** 0.03
KaiRIA [Drosophila melanogaster]

Query	Observed	Mr(expt)	Mr(calc)	Delta	Miss	Score	Expect	Rank	Unique	Peptide
13749	668.1045	1334.1944	1334.6790	-0.4845	1	46	0.043	1	U	R.SRNSSQSIESLK.T

Proteins matching the same set of peptides:

gi|61675663 **Mass:** 101564 **Score:** 46 **Matches:** 1(1) **Sequences:** 1(1)
RE24732p [Drosophila melanogaster]

gi|194899712 **Mass:** 101589 **Score:** 46 **Matches:** 1(1) **Sequences:** 1(1)
GG15269 [Drosophila erecta]

gi|195353927 **Mass:** 86610 **Score:** 46 **Matches:** 1(1) **Sequences:** 1(1)
GM23173 [Drosophila sechellia]

gi|195498265 **Mass:** 101563 **Score:** 46 **Matches:** 1(1) **Sequences:** 1(1)
GE25051 [Drosophila yakuba]

gi|195569399 **Mass:** 101626 **Score:** 46 **Matches:** 1(1) **Sequences:** 1(1)
GD20046 [Drosophila simulans]

---

19. gi|195023900 **Mass:** 190030 **Score:** 46 **Matches:** 1(1) **Sequences:** 1(1) **emPAI:** 0.02
GH20904 [Drosophila grimshawi]

Query	Observed	Mr(expt)	Mr(calc)	Delta	Miss	Score	Expect	Rank	Unique	Peptide
12330	593.1379	1184.2612	1184.6302	-0.3690	1	46	0.029	1	U	K.VFHLGGGGDAKK.K

---

20. gi|195488195 **Mass:** 66917 **Score:** 46 **Matches:** 1(1) **Sequences:** 1(1) **emPAI:** 0.05
GE14061 [Drosophila yakuba]

Query	Observed	Mr(expt)	Mr(calc)	Delta	Miss	Score	Expect	Rank	Unique	Peptide
15913	775.3869	1548.7593	1549.7554	-0.9961	1	46	0.039	1	U	R.MEPMKDLMAVLEK.L

---

21. gi|195126431 **Mass:** 66814 **Score:** 46 **Matches:** 1(1) **Sequences:** 1(1) **emPAI:** 0.05
GI13073 [Drosophila mojavensis]

Query	Observed	Mr(expt)	Mr(calc)	Delta	Miss	Score	Expect	Rank	Unique	Peptide
10435	517.1084	1032.2022	1032.5604	-0.3582	0	46	0.035	1	U	K.VIDTGYPIR.V

Proteins matching the same set of peptides:

gi|195442758 **Mass:** 74458 **Score:** 46 **Matches:** 1(1) **Sequences:** 1(1)
GK24170 [Drosophila willistoni]

---

22. gi|16554779 **Mass:** 15812 **Score:** 44 **Matches:** 1(0) **Sequences:** 1(0) **emPAI:** 0.22
no on or off transient A [Drosophila virilis]

Query Observed Mr(expt) Mr(calc) Delta Miss Score Expect Rank Unique Peptide

15089	723.9612	2168.8617	2168.9154	-0.0537	1	44	0.086	1	U	R.ARGGGGGGGGGGGGGGGGGGGGGGGGGGGGGGGR.D

Mascot: http://www.matrixscience.com/
