# Supplementary material for: Evidence for the major role of PH4αEFB in the prolyl 4-hydroxylation of Drosophila collagen IV
Source: bioRxiv. 2025 Aug 7:2025.08.05.668786. Preprint. [Version 2] doi: 10.1101/2025.08.05.668786 (PMC12340787; doi:10.1101/2025.08.05.668786)
Supplement: Supplement 7 [file NIHPP2025.08.05.668786v2-supplement-7.pdf]

bioRxiv preprint doi: https://doi.org/10.1101/2025.08.05.668786; this version posted August 7, 2025. The copyright holder for this preprint (which was not certified by peer review) is the author/funder, who has granted bioRxiv a license to display the preprint in perpetuity. It is made available under aCC-BY 4.0 International license.

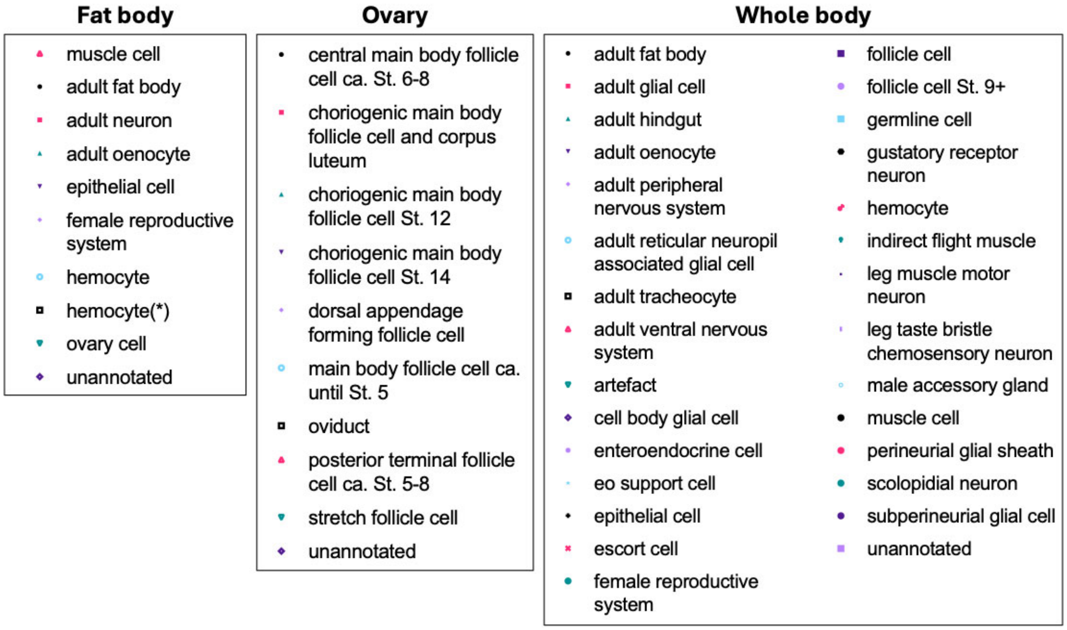

Figure S1. Full legend for the symbols in Figure 3B.

bioRxiv preprint doi: https://doi.org/10.1101/2025.08.05.668786; this version posted August 7, 2025. The copyright holder for this preprint (which was not certified by peer review) is the author/funder, who has granted bioRxiv a license to display the preprint in perpetuity. It is made available under aCC-BY 4.0 International license.

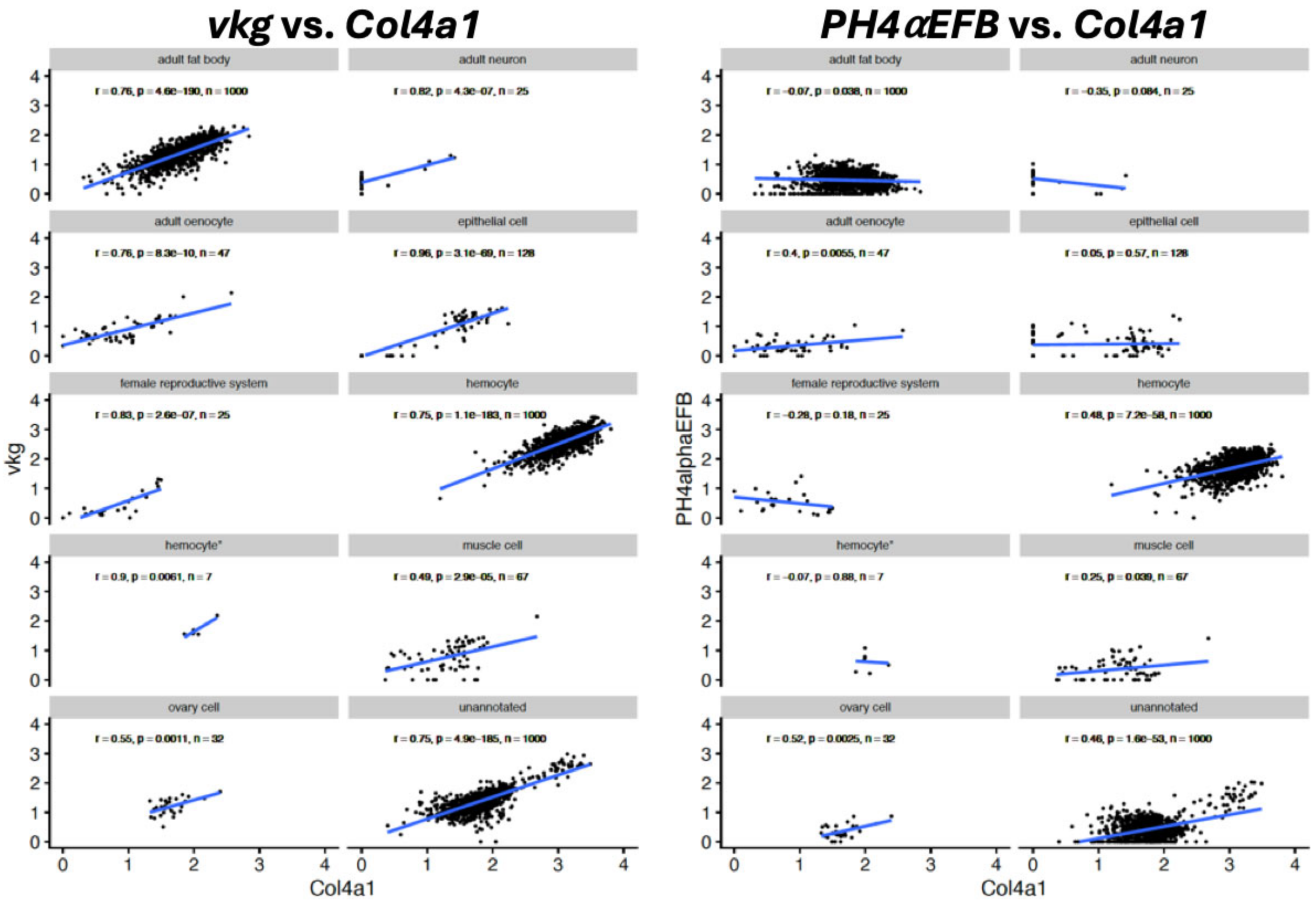

Figure S2. Expression of *Col4a1*, *vkg,* or *PH4αEFB* separately analysed for each different cell type in the fat body dataset.

Regression lines of the plots, correlation coefficients between the two genes plotted (*r*), the *p* values to obtain the results from the null hypothesis that the slope of the regression lines are zero, and the numbers of metacells analysed (n) are shown.

bioRxiv preprint doi: https://doi.org/10.1101/2025.08.05.668786; this version posted August 7, 2025. The copyright holder for this preprint (which was not certified by peer review) is the author/funder, who has granted bioRxiv a license to display the preprint in perpetuity. It is made available under aCC-BY 4.0 International license.

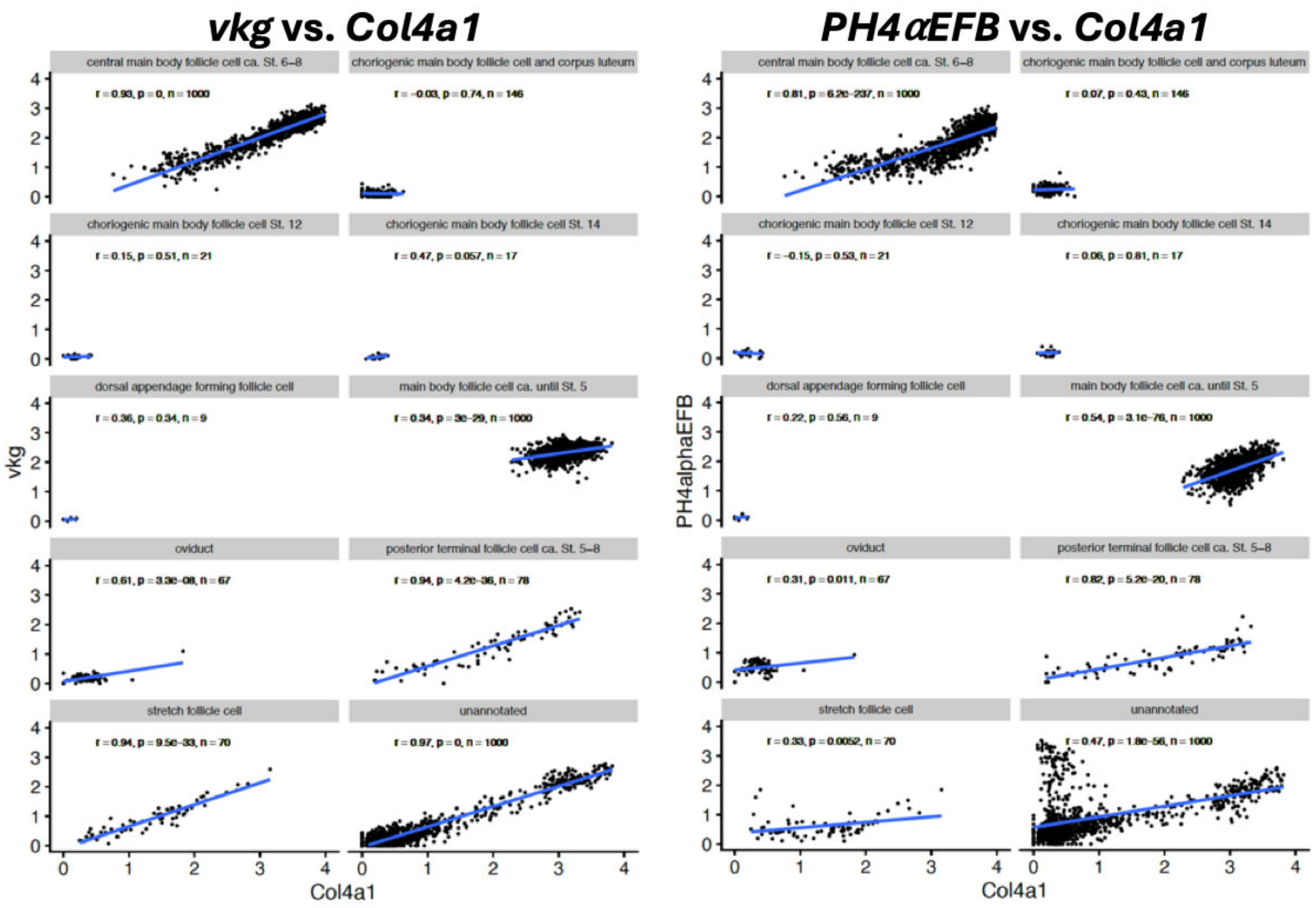

Figure S3. Expression of *Col4a1*, *vkg,* or *PH4αEFB* separately analysed for each different cell type in the ovary dataset.

Regression lines of the plots, correlation coefficients between the two genes plotted (*r*), the *p* values to obtain the results from the null hypothesis that the slope of the regression lines are zero, and the numbers of metacells analysed (n) are shown.

bioRxiv preprint doi: https://doi.org/10.1101/2025.08.05.668786; this version posted August 7, 2025. The copyright holder for this preprint (which was not certified by peer review) is the author/funder, who has granted bioRxiv a license to display the preprint in perpetuity. It is made available under aCC-BY 4.0 International license.

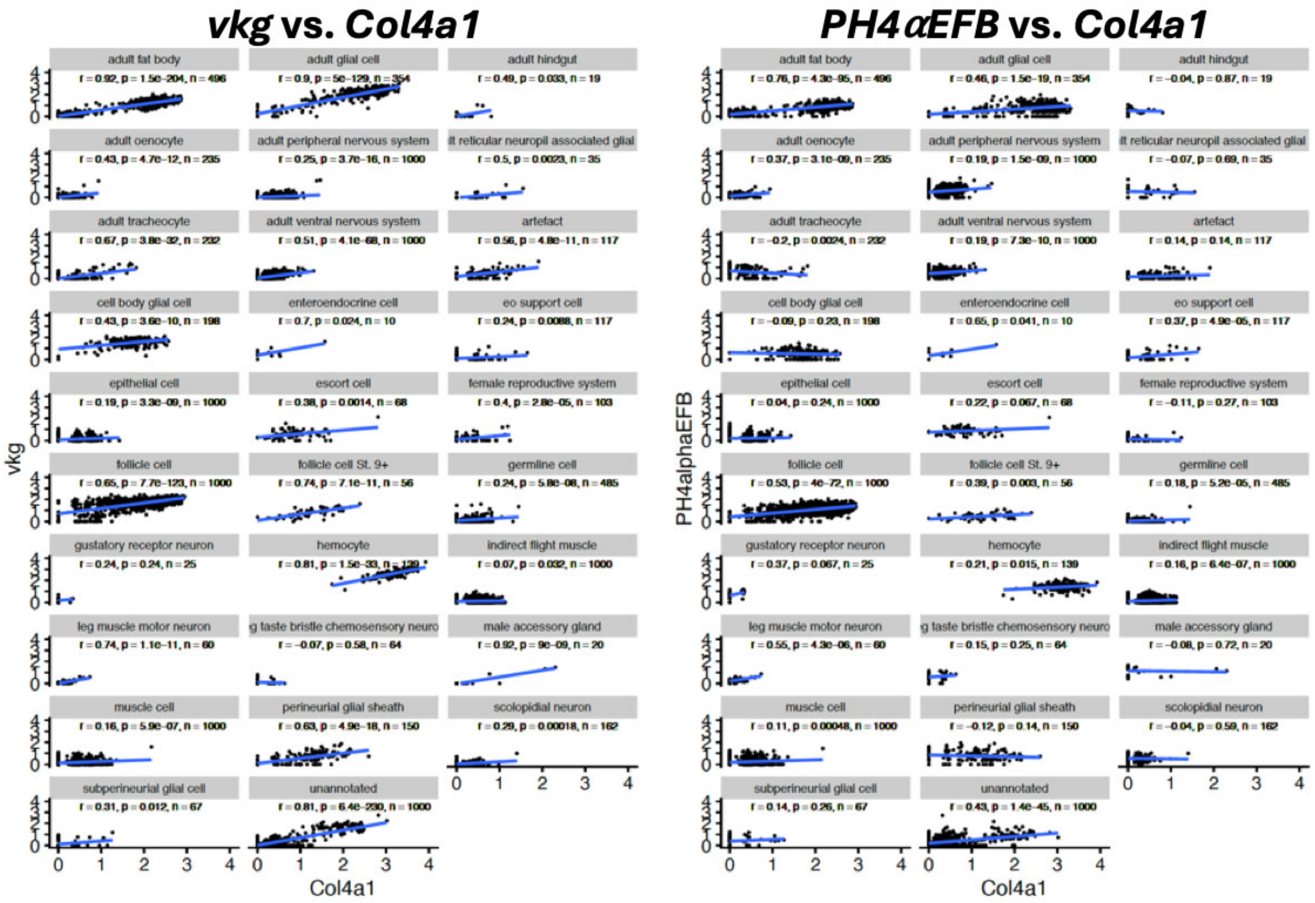

Figure S4. Expression of *Col4a1*, *vkg,* or *PH4αEFB* separately analysed for each different cell type in the whole-body dataset.

Regression lines of the plots, correlation coefficients between the two genes plotted (*r*), the *p* values to obtain the results from the null hypothesis that the slope of the regression lines are zero, and the numbers of metacells analysed (n) are shown.

bioRxiv preprint doi: https://doi.org/10.1101/2025.08.05.668786; this version posted August 7, 2025. The copyright holder for this preprint (which was not certified by peer review) is the author/funder, who has granted bioRxiv a license to display the preprint in perpetuity. It is made available under aCC-BY 4.0 International license.

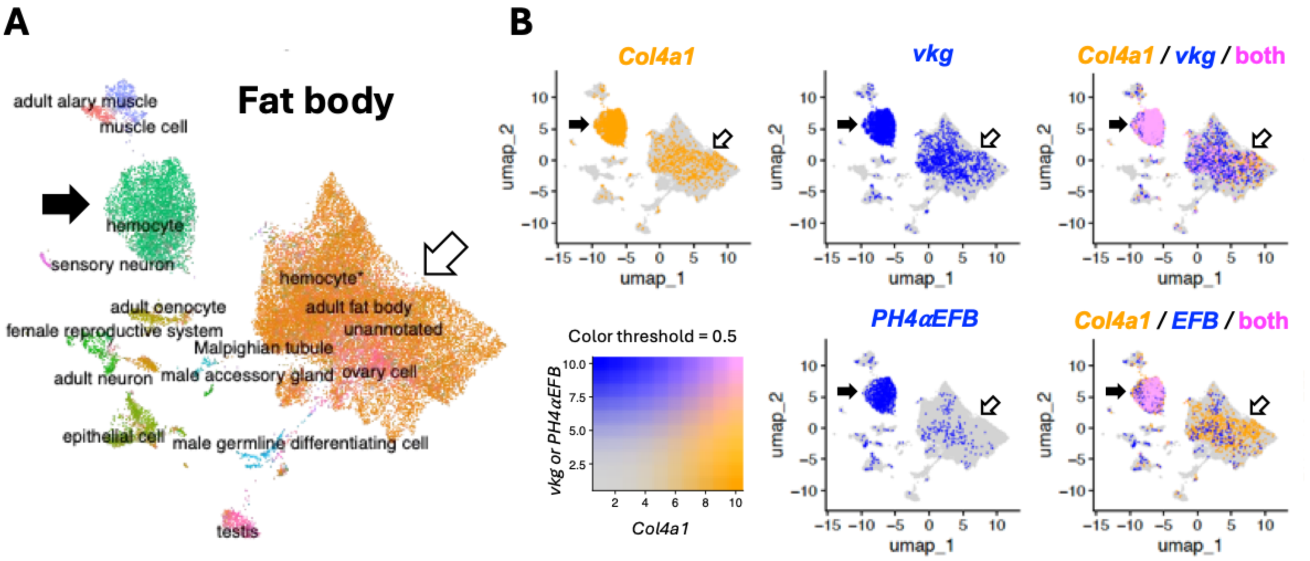

Figure S5. UMAP for the fat body single cell data.
(A) UMAP showing the entire cells with annotations, with different cell types coded by different colour.
(B) Expression of *Col4a1*, *vkg,* and *PH4αEFB (EFB)* colour coded as in the bottom left panel. Top left and middle panels show single gene expression; right panels show overlap. Closed arrows, hemocytes in which the three genes are co-expressed; open arrows, adult fat body cells in which the co-expression of the collagen IV genes and *PH4αEFB* is not as clear as that in the metacell data in Fig. 3B.

bioRxiv preprint doi: https://doi.org/10.1101/2025.08.05.668786; this version posted August 7, 2025. The copyright holder for this preprint (which was not certified by peer review) is the author/funder, who has granted bioRxiv a license to display the preprint in perpetuity. It is made available under aCC-BY 4.0 International license.

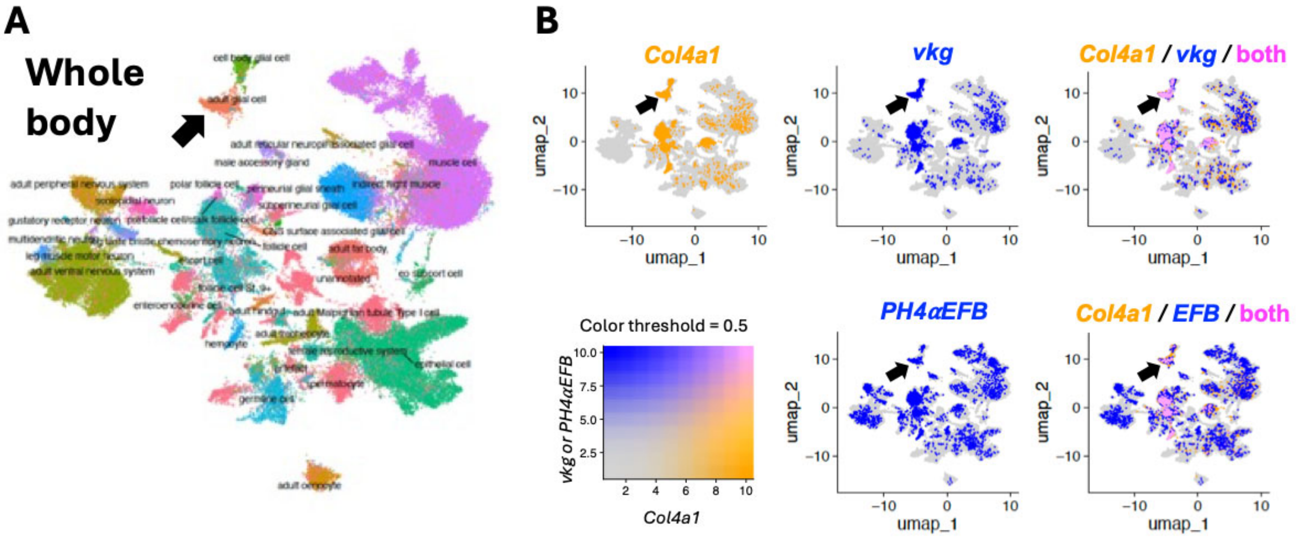

Figure S6. UMAP for the whole-body single cell data.
(A) UMAP showing the entire cells with annotations, with different cell types coded by different colour.
(B) Expression of *Col4a1*, *vkg,* and *PH4αEFB (EFB)* colour coded as in the bottom left panel. Top left and middle panels show single gene expression; right panels show overlap. Closed arrows, glial cells in which the three genes are co-expressed.
